# The role of hydrophobic collapse in cytotoxic and functional amyloid oligomerization

**DOI:** 10.1016/j.bpj.2025.07.042

**Published:** 2025-08-06

**Authors:** Kelsie M. King, Hajar Zaheer, Anne M. Brown

**Affiliations:** 1 Department in Genetics, Bioinformatics, and Computational Biology, Virginia Tech, Blacksburg, Virginia; 2 Department of Biochemistry, Virginia Tech, Blacksburg, Virginia; 3 Data Services, University Libraries, Virginia Tech, Blacksburg, Virginia; 4 Virginia Tech Center for Drug Discovery, Virginia Tech, Blacksburg, Virginia; 5 Academy of Integrated Science, Virginia Tech, Blacksburg, Virginia

## Abstract

“Amyloid” refers to an insoluble, highly organized protein fibril composed of intermolecular *β* sheets, known as a cross-*β* motif. Amyloidogenic proteins are generally driven to aggregate into tightly packed fibrils. Some amyloids are functional, often being utilized as hormone storage reservoirs. The functional, paracrine signaling neuropeptide *β*-endorphin (*β*E) is stored and released to modulate pain responses. Conversely, the function of amyloid-*β* (A*β*), involved in Alzheimer’s disease, is uncertain—but substantial evidence exists of its role in neuronal cell apoptosis. Although both peptides are mechanistically linked in their propensity to adopt fibrillar structures, the biophysical characteristics that drive divergence in cytotoxic potential are not well understood. To probe the conformational dynamics and mechanisms of functional and cytotoxic oligomer formation, we utilized all-atom molecular dynamics to simulate the formation of monomeric and hexameric A*β*_42_ and *β*E_31_. Monomeric A*β*_42_ and *β*E_31_ selectively sampled *β* strand motifs comprising hydrophobic residues, adopting a collapsed state. Cluster analysis indicates that *β*E_31_ hexamers were more conformationally diverse than those sampled by A*β*_42_, suggesting that *β*E_31_ exhibits more signatures of disorder. A*β*_42_ hexamer formation was driven by hydrophobic packing of collapsed *β* strand motifs, where *β*E_31_ hexamer peptide subunits remained structurally plastic and solvent accessible. Mutation of A*β*_42_ disrupting the C-terminal hydrophobic sequence inhibited hydrophobic *β* strand formation, reduced aggregation propensity, and increased solvent accessibility, suggesting that retention of a collapsed state is critical for aberrant oligomer formation. This work provides a preliminary view of cytotoxic and functional oligomer morphologies at atomistic resolution, gaining insights into the biophysical aspects of early aggregation events of amyloids.

## Significance

Amyloidogenic proteins have the propensity to form dense, fibrillar structures. Amyloid fibrils serve a biological function, typically used as a hormonal storage reservoir. However, amyloid proteins are prone to misfolding, aggregating into cytotoxic species that are implicated in several disease states. Understanding the biophysical characteristics of cytotoxic amyloid aggregation is important for understanding amyloid-related pathologies. This work computationally investigates the aggregation of amyloid-*β*_42_ (A*β*_42_)—the cytotoxic species in Alzheimer’s disease—and *β*-endorphin_31_ (*β*E_31_), a noncytotoxic and functional amyloid species. Comparing the aggregation of cytotoxic and functional amyloids highlights the importance of hydrophobic sequences in stabilizing A*β*_42_ aggregates, whereas the charge density distribution of *β*E_31_ reduces aggregation potential. This work provides a basis for understanding the biophysical aspects driving the amyloid cytotoxicity-functionality continuum.

## Introduction

Amyloids, defined as intrinsically disordered proteins that polymerize into a cross-*β* structures (1,2,3), exhibit a unique propensity for modulating secondary and tertiary structures in response to environmental stimuli (4). Amyloid peptides simultaneously exhibit conformational flexibility and the ability to form organized fibrils, conferring them a wide range of functions to amyloidogenic species. Amyloids serve functions such as hormonal signaling and storage (5,6), biofilm formation (7), and antimicrobial activity (8,9) and are hypothesized to have been biomolecular scaffolds in prebiotic environments (4,10). The unique conformational flexibility exhibited by amyloids, however, can give rise to misfolded, cytotoxic oligomeric structures implicated in multiple diseases (11,12,13). Despite multifaceted functionality, low-molecular-weight oligomers formed during amyloid aggregation events can be highly cytotoxic (13,14). Alzheimer’s disease (AD) and type 2 diabetes mellitus (T2D)—both amyloid-related diseases—are the seventh and eighth leading causes of death globally (15). As such, it is critical to understand oligomeric amyloid structure at a fundamental level related to biological function and to elucidate the mechanisms of disease.

Among the functional amyloidogenic hormones are *β*-endorphins (*β*Es), a class of *μ*-opioid receptor agonists (16,17) produced in pituitary glands (18), the hypothalamus (19), and skin tissues (20,21). *β*Es are a component of the endogenous opioid system and function as analgesics (22) and as stress-response modulators (23). *β*Es are cleavage products of pro-opoimelanocortin and exist in several isoforms, ranging from 27 to 31 residues in length (17,24). The 31-residue isoform (*β*E_31_) is the most biologically active isoform (16), exhibiting 18–33 times the potency of morphine (25). Like several protein hormones, *β*Es are stored in highly concentrated, membrane-bound secretory vesicles as amyloid fibrils (5,26,27). Signaling events, such as changes to pH or salt concentrations, trigger fibril disassembly mediated by a buried glutamate residue and granule exocytosis (6,28,29,30). Although *β*Es are amyloidogenic, their fibrillar or oligomeric species are not implicated in the development of amyloid-related disease states (31). In contrast, amyloid-*β* (A*β*) is an amyloidogenic peptide implicated in the progression of AD (32). A*β* is a 37- to 49-residue cleavage product of the transmembrane amyloid precursor protein (APP) (33); the 42-residue isomer (A*β*_42_) and, to a lesser extent, the 40-residue isomer are the predominant species deposited in amyloid plaques (34). A*β* peptides are highly prone to misfolding into oligomeric structures that kill neuronal cells and induce neurological dysfunction (34). Despite the shared propensity of A*β* and *β*Es to adopt fibrillar morphologies, these peptides vary greatly in their cytotoxic potential, warranting investigation into sequence properties that drive amyloidogenic misfolding.

Amyloid aggregation mechanisms typically follow a sigmoidal growth curve with distinct phases: 1) a nucleation phase (or “lag phase”), 2) an elongation and growth phase, and 3) a saturated, inert fibril phase (35,36,37). In the nucleation phase, transient oligomer assemblies form with a cross-*β* structure to which additional monomeric units can be added, acting as seeds for the elongation phase (37,38,39,40). However, the oligomeric assemblies formed in the lag phase can be highly heterogeneous; an impressive range of cytotoxic A*β*_40_/A*β*_42_ oligomeric species have been characterized, including globular/spherical (41,42,43), proto-fibrillar (39,44,45,46), *β*-barrel (47), and flat-*β* sheet/*β* sandwich (48) morphologies. A similarly wide range of oligomeric intermediates have been identified for other amyloids implicated in other diseases, such as *α*-synuclein (49,50,51,52) and human islet amyloid polypeptide (hIAPP) (53,54), which are implicated in Parkinson’s disease and T2D, respectively. Functional amyloids also adopt a variety of structural states and leverage their conformational flexibility to modulate signaling. For instance, the yeast prion Sup35 leverages conformational diversity to control phenotypic expression and fibril seeding conformations (55,56). There is evidence for the tight control of expression and folding for some functional amyloids, minimizing misfolding potential. This has been demonstrated for Pmel17, a mammalian protein critical to melanosome pigmentation (57,58). However, there are functional amyloids that are also involved in disease states, such as hIAPP, which is involved in glucose homeostasis but contributes to amyloid-related *β* cell loss in T2D, worsening disease progression (59). The lack of disease-state-related conformations sampled by amyloids such as *β*Es raises questions as to whether sequence-specific properties of disease-related amyloids, like A*β*, can be identified and thus targeted in therapeutic interventions. Given that A*β* and *β*Es share mechanistically linked aggregation-prone regions (APRs), it is critical to understand how different APRs are more likely to misfold.

**Figure 1:**
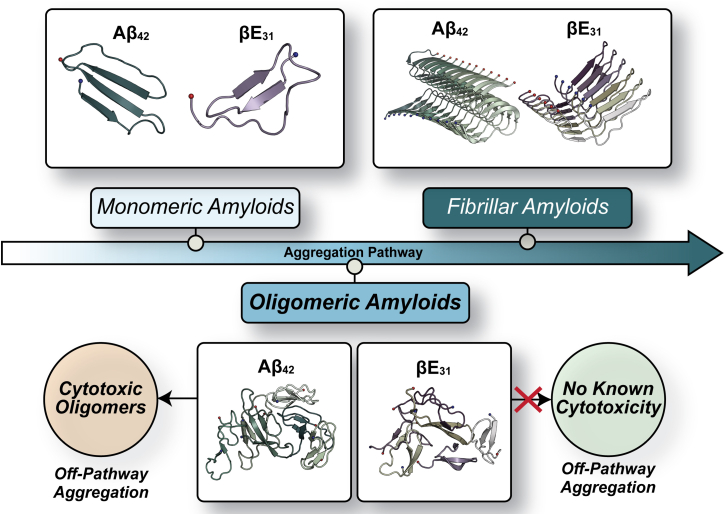
Overview of A*β*_42_ and *β*E_31_ aggregation pathway and propensities for forming off-pathway cytotoxic oligomers.

Computational methods, such as molecular dynamics (MD) simulations, are useful as a complement to experimental techniques in describing the conformational landscape of early oligomeric amyloids at atomistic resolution (60,61,62). Extensive work has been done on the simulation of amyloidogenic fragments, such as residues 16–22 of A*β* (KLVFFAE), the amyloidogenic core (63,64,65,66,67) of A*β*. Simulations of the hexameric 16–22 sequence predict the formation of *β*-barrels (64), much like the cytotoxic cylindrin (68) and the crystallized macrocyclic 16–22 barrels with familial AD mutations (69). Simulations of full-length A*β* peptides and oligomers have recently become more computationally accessible (70,71) and have described a divergence of oligomeric alloforms between compact and extended conformations, the latter of which favor further assembly (71). These alloforms also diverge in their hydrophobic solvent-accessible surface area (SASA), which is postulated in experimental work to give rise to differing cytotoxic potential (72). Given that both cytotoxic and functional amyloids possess hydrophobic APRs (73,74,75), it is currently unclear how oligomerization potential or oligomeric conformations differ among amyloid species across the spectrum of functionality (Fig. 1). To this end, we utilize MD simulations to observe the oligomerization of hexameric A*β*_42_ and *β*E_31_ to explore sequence properties that drive aggregation events and oligomeric architecture. This work seeks to understand morphological differences in cytotoxic and functional oligomer structures to aid our understanding of the genesis of cytotoxic amyloid conformations.

## Materials and methods

### MD Simulations

All MD simulations were constructed with and run using the GROMACS (76,77) software package, v.2016.3 and v.2020.4. The GROMOS96 53a6 (78) force field was employed for all simulations. This force field was chosen due to its superior replication of NMR j-coupling constants in monomeric A*β* simulations (79) and of ordered *β*-barrel structures formed by ^16^KLVFFAE^22^ segments (64). All simulations in this work were employed as follows. Energy minimizations were performed using the steepest descent method. After minimization, a two-step equilibration was performed with position restrains on protein heavy atoms. In the first equilibration step, a 100 ps NVT ensemble was employed using the Berendsen weak coupling method (80,81) at 310 K. In the first equilibration, random velocities were assigned to generate replicate simulation. Subsequently, an isothermal-isobaric NPT ensemble was employed, using the Nosé-Hoover (82) thermostat and the Parrinello-Rahman (83) barostat at 310 K and 1 bar, respectively. After equilibration, production MD runs were performed with all restraints released. Both equilibration and production simulations were performed using a 2 fs integration step. Production simulations utilized the P-LINCS (84) algorithm to constrain bond length. The smooth particle mesh Ewald method (85,86), with cubic interpolation and 0.16 nm Fourier grid spacing, was employed to calculate long-range electrostatic interactions, using a 1.4 nm long-range interaction cutoff. Periodic boundary conditions were employed in all three spatial dimensions.

### System construction

To obtain a monomeric starting structure for insertion into oligomer systems, an initial monomer was simulated for 1 *μ*s to obtain an equilibrated structure in solution. For A*β*42, PDB: 1IYT (87) was utilized, and for *β*E_31_, a single peptide was isolated from the fibril structure in PDB: 6TUB (26). For A*β*_42_^MUT^ (I_31_K/I_32_N, V_36_K/G_37_N), mutations were made to the A*β*_42_ starting structure using PyMOL (88). Starting structures for oligomer simulations were selected by performing root mean-square deviation (RMSD) clustering over the 1 *μ*s trajectories using a 0.3 nm cutoff. To construct the oligomer systems, peptides were placed into a cubic box (12.23 × 12.23 × 12.23 nm for A*β*_42_ and 12.21 × 12.21 × 12.21 nm for A*β*_42_^MUT^ and *β*E_31_) at least 1.4 nm apart to be outside of the long-range interaction cutoff. A minimum 1.0 nm solute-box distance was enforced. Each system was solvated using 150 mM NaCl with additional neutralizing counterions. Monomer simulations were performed in triplicate in the same manner and with the same parameters as described previously. Simulation inputs and starting structures can be found on our lab OSF (89). Monomer simulations for A*β*_42_, *β*E_31_, and A*β*_42_^MUT^ were performed in three replicates, with each replicate simulated for 2 *μ*s. Hexamer simulations for A*β*_42_, *β*E_31_, and A*β*_42_^MUT^ were performed in three replicates, with each replicate simulated for 2 *μ*s. A total of 38 *μ*s of simulation time was performed. Starting structures for the monomeric A*β*_42_, monomeric *β*E_31_, hexameric A*β*_42_, and hexameric *β*E_31_ are shown in Fig. S21. Simulation inputs and starting structures can be found on our lab OSF (90).

### Analysis

Molecular visualization was performed using PyMOL (91). Data collection was performed using both the GROMACS analysis suite and MDtraj (92). Secondary structure was performed using the DSSP algorithm (93). RMSD clustering was performed on protein backbone atoms using the GROMOS method as described by Daura et al. (94), with a 0.3 nm cutoff. Statistical analysis was performed using SciPy (95). For datasets that satisfied assumptions of equal variance and normality, parametric statistical tests were employed. For normal, multilevel datasets (>2), one-way ANOVA was performed in conjunction with Tukey’s HSD for post hoc comparisons, and for two-group comparisons, *t*-tests were used. For datasets that violated assumptions of normality, Kruskal-Wallis in conjunction with Dunn’s multiple comparison test were employed. Statistical difference was defined as p<0.05.

Interaction frequencies were calculated to represent an aggregate of all peptide pairs. When considering intermolecular interactions, interactions for a given residue pair in a hexameric system could be calculated for 15 peptide pairs; similarly, when considering intramolecular interactions, a residue pair interaction could be calculated 6 times in a hexameric system. To simplify the analysis of residue-residue interactions in a multimeric system, we employ a dimensionality reduction technique designed to weight residue-residue interactions based on their frequency (both as an overall occupancy and the frequency across all possible pairs) and the mean distance sampled. For a given residue pair (e.g., Phe4-Phe19), the computation is as follows:

$$Residue-Residue\;Interaction\;Frequency=\frac{{N\;pairs}_{d\leq 0.6}}{\mu_{d\leq 0.6}}\times \frac{N\;frames\;\mu_{d\leq 0.6}}{total\;frames}\text{,}$$

where ${N\;pairs}_{d\leq 0.6}$ equals the number of peptide pairs where the residue pair of interest is within the 0.6 nm interaction cutoff; $\mu_{d\leq 0.6}$ equals the mean of all distances within the interaction cutoff, defined as 0.6 nm; and $N\;frames\;\mu_{d\leq 0.6}$ equals the number of frames for which the residue pair of interest exhibits at least one interaction.

Unless otherwise stated, all analysis and averages presented from hexamer simulations are taken over the entire simulation period. For calculations involving eccentricity or radius of gyration, distributions and averages were taken over the 0.3–2 *μ*s simulation period. This time frame was chosen based on the convergence of the radius of gyration, indicating the formation of a stably compact oligomer structure (Figs. S22 and S23). Boltzmann-weighted 2D histograms and inter/intramolecular interaction heatmaps were computed using concatenated data from all three replicates.

## Results and discussion

Amyloidogenic peptides are expressed in an abundance of species, leveraging their conformational diversity to modify their functional state (96,97,98). The divergence in cytotoxic potential of amyloids may be related to oligomer architecture and dynamics, as oligomeric intermediates are thought to be the primary cytotoxic amyloid species (12,13). However, the influence of sequence on oligomerization dynamics in terms of functional differences is poorly understood. Here, we utilize MD simulations to explore the biophysical properties of monomeric and hexameric A*β*_42_ and *β*E_31_—the former being central to AD pathology. At the time of writing, there are no published computational investigations that use a comparison to functional amyloid dynamics as a methodology to better understand the behavior of cytotoxic oligomer species. Addressing this fundamental knowledge gap is crucial to unraveling the specific biophysical mechanisms that differentiate functional amyloid behavior from cytotoxic processes, as related to amyloid structure-function.

### Monomeric A*β*_42_ and *β*E_31_ exhibit divergence in structural disorder

In their monomeric state, A*β*_42_ and *β*E_31_ are both considered to be intrinsically disordered (99,100,101). However, in its oligomeric form, A*β*_42_ can adopt a wide variety of conformations, including highly organized motifs such as proto-fibrils, *β*-barrels, and *β* sandwiches (39,44,45,46,47,48). These architectures all involve ordered *β* sheet formation, suggesting that monomeric A*β*_42_ can adopt stable, folded states. In the context of comparing cytotoxic and functional amyloid peptides, it could be possible that a more conformationally flexible peptide would be less prone to folding stably, therefore reducing the probability of forming ordered oligomer structures with cytotoxic potential.

**Figure 2:**
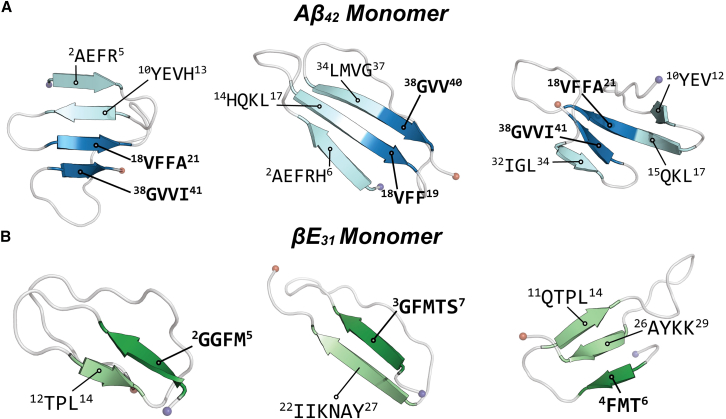
*β* sheet motifs sampled in monomeric amyloid simulations. Snapshots from simulation visualizing predominant *β* strand motifs across replicates for (*A*) A*β*_42_ and (*B*) *β*E_31_ monomers. Most frequent *β* strand residues are colored dark blue or dark green for A*β*_42_ and *β*E_31_, respectively. Variable strands are colored light blue or light green for A*β*_42_ and *β*E_31_, respectively.

To this end, 2 *μ*s simulations of monomeric A*β*_42_ and *β*E_31_ were performed in triplicate to assess their structure and dynamics. Secondary structure calculations indicate that both peptides have a high propensity for adopting *β* strand content (39.7% and 33.2% *β* strand content for A*β*_42_ and *β*E_31_, respectively) (Fig. S1 *A*; Table 1). In all replicates, the A*β*_42_ monomer adopts a *β* sheet involving hydrophobic-core residues ^18^VFF^20^ and hydrophobic C-terminal residues ^38^GVV^40^, engaged in *β* strand content for >90% of all simulation time in aggregate (6 *μ*s) (Fig. 2 *A*). These residues form a core *β* sheet in which other segments can join, typically adopting a sheet comprising 3–4 *β* strands, sampling sheets formed with both N- and C-terminal strands. Principal-component analysis (PCA) of secondary structure content reveals three well-resolved clusters that correspond to each replicate, indicating that any given folded structure is highly stable, changing minimally over the simulation period (Fig. S2 *A*). In contrast, *β*E_31_ exhibits a flatter secondary structure distribution, indicating higher variability in sampled *β* strand content (Fig. S2 *B*). Residues ^3^GFMTS^7^ sample *β* strand content at the highest frequency (∼75% of all simulation time, 6 *μ*s), where adjacent strands forming a sheet or *β*-hairpin with the N-terminus are highly variable (Fig. 2 *B*); residue interaction heatmaps indicate that N-terminal residues ^3^GFMTS^7^ interact uniformly with the rest of the peptide (Fig. S3). Furthermore, PCA of sampled secondary structure content does not reveal well-defined, clustered replicates, indicating increased structural variability (Fig. S2 *B*).

**Table 1:** Secondary structure percentages for A*β*_42_ and *β*E_31_ monomer simulations

Replicate	A*β*_42_ monomer secondary structure (%)			*β*E_31_ monomer secondary structure (%)		
	Coil	β sheet	Helix	Coil	β sheet	Helix
Replicate 1	59.1	40.9	0.02	65.4	34.5	0.07
Replicate 2	55.8	44.1	0.02	61.4	38.5	0.06
Replicate 3	65.8	34.2	0.07	73.4	26.5	0.16
All	60.2	39.7	0.04	66.7	33.2	0.09

**Figure 3:**
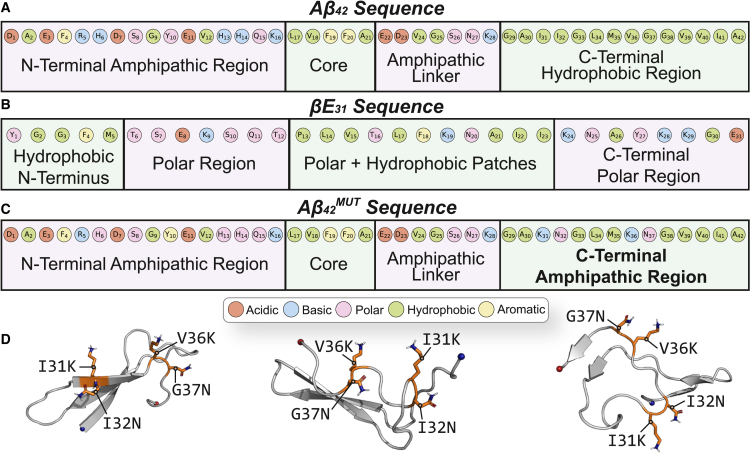
Sequences of A*β*_42_, *β*E_31_, and A*β*_42_^MUT^. (*A–C*) Sequences of (*A*) A*β*_42_, (*B*) *β*E_31_, and (*C*) A*β*_42_^MUT^, colored by residue type (acidic, *red*; basic, *blue*; polar, *pink*; hydrophobic, *green*; aromatic, *yellow*). (*D*) Snapshots of A*β*_42_^MUT^ monomer simulations visualizing mutation disruption to C-terminal secondary structure. Mutated residues are colored as orange sticks.

Although *β*E_31_ and A*β*_42_ both exhibit signatures of disorder, these data imply that *β*Es may exhibit more inherent disorder than A*β*. The A*β*_42_ sequence is distinctly partitioned into four segments, alternating in charge density and hydrophobicity, whereas the *β*E_31_ sequence instead consists of short hydrophobic patches interspersed by polar or charged segments (Fig. 3, *A* and *B*). The hydrophobic segmentation present in the A*β*_42_ sequence likely favors collapsed conformations to minimize hydrophobic solvent exposure. To probe the role of hydrophobic segmentation on monomeric folding, a monomeric A*β*_42_ mutant (I_31_K/I_32_N, V_36_K/G_37_N) (A*β*_42_^MUT^) was simulated in triplicate for 2 *μ*s. The hydrophobic C-terminus was disrupted with KN residue motifs, which appear twice in the *β*E_31_ sequence, interspersed between short hydrophobic patches (Fig. 3 *C*). The A*β*_42_^MUT^ monomer, much like *β*E_31_, exhibits reduced secondary structure continuity relative to A*β*_42_ (Fig. S1 *C*), where PCA of secondary structure is not well resolved by the replicate (Fig. S2 *C*). A*β*_42_^MUT^ monomers exhibited a 35% reduction in hydrophobic core residues (^17^LVFFA^21^ and ^37^GGVVI^41^) relative to wild-type (WT) A*β*_42_ (Figs. 3 *D* and S4). This indicates that the inclusion of bulky, polar residues prevents the hydrophobic collapse of the core and C-terminal residues. Furthermore, the A*β*_42_^MUT^ C-terminal amphipathic segment (^22^EDVGSNK^28^) sampled virtually no *β* strand content in WT A*β*_42_, where it primarily functioned as a disordered linker between the hydrophobic core and C-terminal *β* sheet. In A*β*_42_^MUT^, this segment sampled both *β* strand and helical content (Fig. S1 *C*). The loss of hydrophobic collapse, characteristic of WT A*β*_42_, supports the notion that hydrophobic segmentation favors continuous, *β* sheet-containing monomeric structures. As such, we postulate that the distinct hydrophobic segmentation of the A*β*_42_ is critical to stabilizing monomeric folding, thus stabilizing ordered oligomeric species and contributing to nucleation events.

### *β*E_31_ hexamers are more polymorphic than A*β*_42_

**Figure 4:**
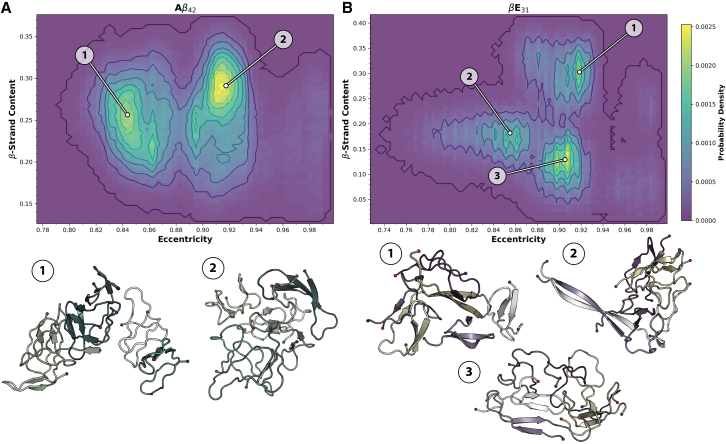
Boltzmann-weighted 2D histograms of eccentricity and *β* strand content. Boltzmann-weighted 2D histograms of eccentricity and *β* strand content for (*A*) A*β*_42_ and (*B*) *β*E_31_ hexamers, taken over the entire 2 *μ*s simulation period across all replicates. Structures below plots correspond to the labeled points on the histograms in terms of *β* strand content and eccentricity. (*A*) A*β*_42_ hexamers are shown as blue/green cartoons, and (*B*) *β*E_31_ hexamers are shown as yellow/purple cartoons.

We simulated the formation of hexameric A*β*_42_ and *β*E_31_ to examine both multiple potential oligomer architectures and the interactions present in each structural state sampled. To examine overall oligomer architectures, RMSD clustering was performed, and dominant morphologies were visualized (Fig. S5). Visually, the A*β*_42_ hexamer structures sampled can be described as a continuum between 1) a globular, spherical, and relatively disordered hexamer and 2) an elongated, *β* strand-rich hexamer. 2D probability distributions of sampled eccentricity and *β* strand content indicate two regions of high probability, consistent with the dichotomy of observed conformational states corresponding to a relatively spherical/disordered state (globular) and a rod-shaped/folded state (elongated) (Fig. 4 *A*; Table 2). Replicates 1 and 2 primarily sampled the elongated and globular states, respectively, whereas replicate 3 transitioned from elongated to globular over the simulation period (Figs. 4 *A* and S5). Elongated and globular conformations sampled in this work are compatible with experimental characterizations; experimental characterizations of A*β*_42_ oligomer morphologies have reported several conformational states, including globular (41,42,43), proto-fibrillar (39,44,45,46), *β* sandwich (48), and *β*-barrel species (47). Sampling of highly organized, experimentally reported conformations, such as *β*-barrels, was absent; achieving sampling of such organized conformational states would likely require enhanced sampling techniques. In contrast, *β*E_31_ hexamers were highly diverse, exhibiting little heterogeneity across replicates in terms of *β* strand content and eccentricity (Figs. 4 *B* and S6). The 2D probability distribution of eccentricity and *β* strand content indicate three regions of high probability, each corresponding to a given replicate that sampled a distinct structure: 1) a roughly circular arrangement of *β* strands, 2) a *β* strand dimer and a tetramer of mixed secondary structure character, and 3) a globular, largely disordered hexamer with transient α-helical content (Fig. 4 *B*; Table 2). The relative diversity of *β*E_31_ hexamer structures suggests a rougher, more diverse free energy landscape in terms of oligomer architectures. It should be noted that in this study, we have performed MD with a conventional NPT ensemble. As such, the architectures sampled in this work are by no means exhaustive. Advanced sampling techniques, like replica exchange (102,103) and Gaussian accelerated MD (104,105) are more robust methods for sampling a wide conformational space.

**Table 2:** Centroids of Boltzmann-weighted 2D histograms of eccentricity and *β* strand content for A*β*_42_ and *β*E_31_ hexamers

Centroid	A*β*_42_ hexamer		*β*E_31_ hexamer	
	Eccentricity	β strand (proportion)	Eccentricity	β strand (proportion)
1	0.84	0.25	0.90	0.31
2	0.91	0.29	0.83	0.23
3	–	–	0.91	0.19

Centroids were determined by K-means clustering of eccentricity and *β* strand content, taken over the entire 2 *μ*s simulation period across all replicates.

**Figure 5:**
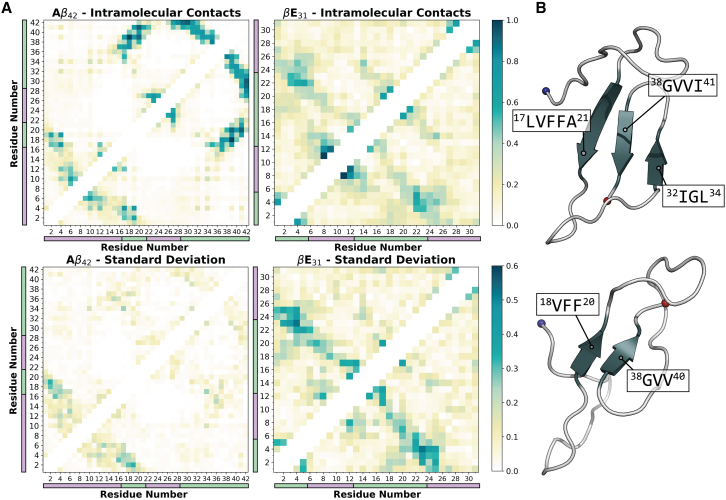
Intramolecular interaction probability heatmaps. (*A*) Intramolecular interaction probability heatmaps for (*top*) A*β*_42_ and (*bottom*) *β*E_31_ hexamer simulations. Purple and green bars correspond to residue regions as labeled in Fig. 3, *A* and *B*, for A*β*_42_ and *β*E_31_, respectively. Heatmaps represent a composite probability of all three replicates over the 2 *μ*s simulation period. (*B*) Visualizations of predominant A*β*_42_ secondary structure motifs. Coils are shown as gray cartoon, and *β* strands are shown as dark blue cartoon. The N- and C-termini are shown as blue and red spheres, respectively.

Examination of intramolecular interactions reveal mechanistic insights into the relative homogeneity of A*β*_42_ hexamers with respect to *β*E_31_. Intramolecular interaction probabilities between hydrophobic core residues (^16^KLVFFAE^21^) and hydrophobic C-terminal residues for A*β*_42_ peptides are sampled consistently and uniformly across replicates, reflecting the preservation of stable *β* strand motifs sampled in monomeric simulations (Figs. 5 *A* and S7). The peptide subunits of the hexamer exhibit a strong preference for adopting *β* sheet motifs, including a C-terminal β-turn-β motif comprising residues ^32^IIGLM^35^ and ^38^GVVI^41^ and a *β* sheet comprising ^17^LVFFA^21^, ^38^GVVI^41^, and ^32^IIGLM^35^ (Figs. 5 *B*, S8, and S9), consistent with NMR observations (106,107). Previous studies have found that the A*β*_42_ intramolecular *β*-hairpin structures are involved in the formation of intermolecular *β* sheets (108,109,110). Counts of intermolecular *β* strand content indicate that across replicates, hydrophobic C-terminal residues—particularly ^32^IIGLM^35^ and ^38^GVVI^41^—consistently engage in intermolecular *β* sheets in all replicates, consistent with previous observations (Fig. S10). Intramolecular interactions in the amphipathic A*β*_42_ N-terminus exhibit more variability across replicates. Indeed, for A*β*_42_, interactions between residues with polar or charged side chains are associated with higher variance in interaction propensity across replicates and peptides (p≤0.5) (Fig. S11).

Intramolecular interactions sampled by *β*E_31_ exhibit high variance across peptides and replicates, indicating a higher degree of disorder relative to A*β*_42_ (Fig. 5 *A*). The *β*E_31_ sequence is charge dense relative to A*β*_42_, whose well-defined hydrophobic regions likely favor a collapsed state, as observed in monomeric simulations. R_*g*_ calculations on peptide subunits indicate that oligomerized A*β*_42_ peptides are more compact relative to *β*E_31_ peptides (A*β*_42_ mean = 1.09 ± 0.01 nm, *β*E_31_ mean = 1.24 ± 0.03 nm) (Fig. S12). A*β*_42_ *β*-hairpin motifs in tandem with an unstructured N-terminus have been resolved via native ion mobility-mass spectrometry, consistent with the observed conformations (47). This supports the concept that A*β*_42_ hydrophobic segmentation leading to collapsed *β* strand motifs contributes to the stabilization of oligomer conformations.

The intermolecular interactions present in the simulated hexamers were examined by calculating interaction probabilities (Fig. S13). Despite sampling two distinct architectures in terms of *β* strand content and eccentricity, each of the three A*β*_42_ replicates displayed a unique interaction profile. Replicate 1 exhibited well-distributed contacts across all regions of the peptide. The highest-frequency interactions in replicate 1 were present between 1) hydrophobic core residues (^17^LVFFA^21^) and the C-terminus (^29^GAIIGL^34^) and 2) N-terminal residues on adjacent chains (^5^RHDSGY^10^). In replicate 2, prevalent interactions included both C-terminal/C-terminal interactions (^29^GAI^31^ with ^34^LMV^36^) and interactions between the amphipathic linker region (^22^EDVG^25^) and the polar N-terminus (^13^HHQK^16^). In replicate 3, interactions between the 16–23 segment (^16^KLVFFAED^23^) and the N-terminus (^1^DAEFRGDS^8^) were the most prominent. In terms of *π*-stacking, Tyr10 exhibited a relatively high propensity for interactions with other aromatic residues, although its interactions were nonspecific (Phe4, His13, Phe19, and Phe20) (Fig. S14). Stacking between Phe4/Phe20, Phe4/Tyr10, and Phe4/Phe20 was commonly sampled, suggesting a role of Phe4 and Tyr10 in coordinating the interaction between the amphipathic N-terminus and the hydrophobic core. Interestingly, mutating Tyr10 to a synthetic residue (para-amino-phenylalanine) impairs the aggregation propensity of A*β*_42_, suggesting that the hydroxyl group is important for aggregating stability in addition to aromaticity (111). However, analysis of intermolecular hydrogen bonds over the simulation indicates that hydrogen bonds involving Tyr10 were not sampled (Fig. S15). Salt bridge formation was also assessed (Table S2). The residue with the highest probability to engage in a salt bridge (∼33%–75% of the aggregated simulation period) was the N-terminal Asp1, which was involved in almost all high-occupancy salt bridges. These salt bridges formed via both the amino and carboxylic groups. The most probable salt bridge partners for Asp1 include Asp1, Asp7, Lys16, and Lys28.

*β*E_31_ hexamers, like A*β*_42_, each display a unique intermolecular interaction profile (Fig. S13). Replicate 1 exhibits interactions between the N-terminal polar region (^6^TSEKSQT^12^) and the C-terminal polar region (^26^KNAYKKGE^31^). This interaction is likely mediated by opposing charges present in either region. A salt bridge between Lys9 and Glu31 is sampled by two separate peptide chains, present for 12.7%–16.5% of the aggregated period (Table S3). This replicate also exhibited interactions between the N-terminus (^1^YGGFM^5^) and the 16–20 segment (^17^TLFKN^20^), mediated primarily by the stacking of Phe4 and Phe18 (Fig. S14). For replicate 2, interaction probabilities reflect the formation of a dimeric, parallel *β* strand along most of the peptide (Fig. 4 *B*). The four remaining peptides display interactions primarily between N-terminal segments, ^1^YGGFM^5^, and ^8^EKSQTP^13^. Replicate 3 displayed interactions between adjacent ^19^KNAIIK^24^ regions, between adjacent ^12^TPLVT^16^ regions, and between 19KNAIIK^24^ and ^12^TPLVT^16^ regions. The propensity for *π*-stacking in replicate 1 and the high probability of interactions between small hydrophobic patches suggest that hydrophobic packing is also critical to the association of *β*E_31_ peptides. The hydrophobic packing present in both A*β*_42_ and *β*E_31_ oligomer formation is consistent with the burial of hydrophobic APRs.

To further assess the role of hydrophobic packing on hexamer formation, we assessed the SASA of each residue, normalized by residue size, over the oligomerized simulation period (0.3–2 *μ*s) (Fig. S16 *A*). A*β*_42_ oligomers form such that solvent exposure of hydrophobic residues is minimized; among all replicates, Phe4, Leu17, Phe19, Val39, and Ile41 are the least solvent-exposed residues in all replicates (range: 0.015 ± 0.012–0.17 ± 0.05 ${\text{nm}}^{2}\;n_{\text{atoms}}^{-1}$), indicating that the packing of these residues is involved in hexamer formation. Like A*β*_42_, *β*E_31_ also exhibited hydrophobic packing, where the SASAs of Gly3, Phe4, Thr6, Thr12, Val15, Ala21, and Gly30 were minimized (Fig. S16 *B*). The least solvent-exposed residue in all *β*E_31_ hexamer simulations—Phe4 in replicate 2—exhibited a normalized SASA of 0.13 ± 0.05 ${\text{nm}}^{2}\;n_{\text{atoms}}^{-1}$, indicating that *β*E_31_ hexamers were overall more solvent accessible than A*β*_42_ hexamers, despite their smaller size. This suggests that hydrophobic packing exhibited by *β*E_31_ hexamers is less efficient. To assess the stability of intermolecular interactions in the hexameric structures, interaction occupancies were calculated for every residue pair. A*β*_42_ appears to have a higher proportion of interaction occupancies that persist for >60% of the simulation, suggesting that intermolecular contacts formed by A*β*_42_ peptides are, to some degree, more stable (Fig. S17). Interestingly, experimental studies of coaggregated A*β*_42_ and *β*E_31_ indicate that *β*E_31_ has a higher propensity for interacting with A*β*_42_ than with other *β*E_31_ peptides, thus reducing A*β*_42_ aggregation and mitigating cytotoxicity (112). This suggests that the hydrophobicity of *β*E_31_ APRs is not significant enough to drive disease-related oligomerization. Additionally, the aggregation of *β*E_31_ is dependent on heparan sulfate (26). Together, these data support the role of hydrophobic APRs in A*β*_42_ oligomerization events (113,114) and may indicate that peptides lacking defined hydrophobic regions are less prone to aberrant aggregation events.

### C-terminal mutations destabilize A*β*_42_ hexameric stability

Previous computational work has identified the hydrophobic core/C-terminal *β*-hairpin to be critical in the formation of A*β* oligomers (107,108,109,110,115). Furthermore, *β*-hairpins are the constituents of cytotoxic *β*-barrels formed from amyloidogenic segments (68,69,116). To further explore the impact of A*β*_42_ hydrophobic segmentation on oligomeric conformational dynamics, the formation of A*β*_42_^MUT^ hexamers was simulated for 2 *μ*s in triplicate. Interestingly, A*β*_42_^MUT^ oligomers sampled higher *β* strand content overall, but ^18^VF^19^/^38^GVV^40^ *β* strand content was significantly reduced in A*β*_42_^MUT^ oligomers relative to A*β*_42_ (A*β*_42_^WT^) oligomers (Fig. S18, *A* and *B*). Global increases to *β* strand content probabilities were observed for N-terminal residues 4–17 and amphipathic linker residues 21–24. In A*β*_42_^WT^ oligomers, residues ^35^MVG^37^ have a high probability of serving as a linker between ^32^IGL^34^ and ^38^GVV^40^ *β* strand segments; these residues have an increased probability of participating in *β* sheets in A*β*_42_^MUT^ oligomers (Fig. S18 *C*). To probe the stability of *β* strand content sampled by the C-terminus in aggregate, the pairwise Hamming distance of secondary structure content in residues 28–42 was calculated over all frames and replicates for A*β*_42_^WT^ and A*β*_42_^MUT^ hexamers. On average, A*β*_42_^MUT^ exhibited higher variation relative to A*β*_42_^WT^ (4.7 ± 2.5 vs. 3.4 ± 1.9, respectively), suggesting that the amphipathic *β* sheets formed are less stable than those formed by hydrophobic sequences (Table S4).

**Figure 6:**
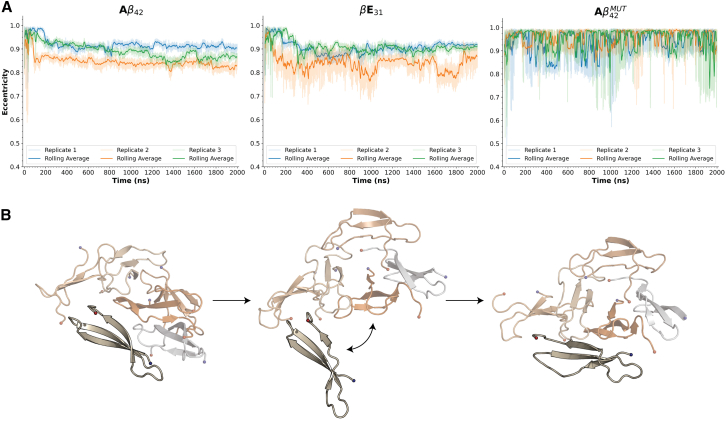
Eccentricity over time from hexamer simulations. (*A*) Eccentricity over time for A*β*_42_, *β*E_31_, and A*β*_42_^MUT^. Rolling average (window = 1000 frames) is shown as a dark line, and the raw eccentricity data are shown as a lighter fill. (*B*) Snapshots from the simulation of A*β*_42_^MUT^ demonstrating repeated association and disassociation of peptides from the core oligomer.

WT A*β*_42_ oligomer morphologies primarily sampled two modes of eccentricity corresponding to globular and elongated morphologies, exhibiting relatively low variance across the simulation period (Figs. 6 *A* and S19). Similarly, *β*E_31_ oligomers exhibited both high- and low-eccentricity states, albeit with slightly more variation, on average, relative to A*β*_42_. In contrast, A*β*_42_^MUT^ exhibited rapid interconversions between globular and extended conformations. Upon examination of dominant morphologies from RMSD clustering, the large fluctuations in eccentricity can be attributed to repeating associations and disassociations of individual peptides or larger units (Figs. 6 *B* and S19). Hydrophobic SASA is significantly increased in A*β*_42_^MUT^ oligomers with respect to WT oligomers (61.42 ± 3.24 vs. 56.25 ± 2.56 nm^2^*,* respectively), despite the overall maximum hydrophobic SASA having been reduced because of mutations (Fig. S20). This reflects a reduced propensity for hydrophobic packing, and suggests that the intermolecular contacts formed, which largely shifted to favor polar contacts, are generally less favorable than those in A*β*_42_ and *β*E_31_ oligomers. Hexamers losing their structural integrity when polar mutations are inserted into the C-terminus further supports the role of C-terminal hydrophobicity in stabilizing oligomeric structures and the role of charge distribution in destabilizing amyloid oligomeric structures.

## Conclusions

We have demonstrated that A*β*_42_ hexamers adopt highly stable intramolecular contacts and, although polymorphic, adopt predictable global architectures, partly influenced by the hydrophobic collapse of individual peptides. The functional amyloid *β*E_31_, and the theoretical A*β*_42_^MUT^, in contrast, exhibit more varied conformational landscapes. Based on these findings, we propose potential connections to how these biophysical properties may influence the broader cytotoxic potential of A*β*_42_ oligomers. First, the A*β*_42_ sequence contains hydrophobic APRs. The ^16^KLVFFAE^22^ segment of A*β*, a hydrophobic sequence flanked by charged residues, readily self-assembles into fibrils (117). Other small amyloidogenic segments, like ^20^SNNFGAILSS^29^ of hIAPP, are capable of self-assembly, aggregating primarily via hydrophobic packing of Phe23 residues (64,118). These APRs could theoretically drive uncontrolled aggregation characteristic of disease states, whereas *β*E_31_ aggregation in vivo is assisted by glycosaminoglycans present in secretory granules (26).

A second important observation is that disease-related amyloids, like A*β*_42_, can exert their cytotoxic effects via membrane perturbations (48,119,120,121,122). Two commonly proposed mechanisms of membrane perturbation include the formation of *β*-barrel pores (121,123), and via lipid extraction (122). The formation of *β*-barrel pores may be a thermodynamically spontaneous conformational state to reduce unfavorable hydrophobic interactions with solvent. Theoretical studies of computationally derived A*β*_42_ *β*-barrels consist of hydrophobic residues building the barrel core and N-terminal and amphipathic linker residues remaining disordered in solution (47). Predictive models (91) for transmembrane protein sequences have been developed using hydrophobicity scales (90,124) as parameters. Although hydrophobicity is not the only feature that controls transmembrane regions or membrane insertion, it is an important property that the A*β*_42_ satisfies. Finally, the hydrophobic collapse driven by the A*β*_42_ sequence is theoretically suited to act as a detergent or otherwise bind and sequester lipids in the environment. The partitioning of polar and hydrophobic regions in folded A*β*_42_ structures could accommodate simultaneous binding with hydrophobic lipid tails and zwitterionic polar heads. Experimental work has determined that free lipids present at critical concentrations alter amyloid oligomer morphologies and mechanisms of cytotoxicity(123), and lipids have been resolved as components of matured A*β* fibrils in vivo (125,126). The sequences of functional amyloids, like that of *β*E_31_, may be less amenable to barrel formation or lipid binding due to more evenly dispersed charge density, lacking the hydrophobic surface area necessary for such conformations to be feasible.

In this work, we have identified the hydrophobic segmentation of A*β*_42_ as a critical component in stabilizing the oligomeric structures sampled in this work. Monomeric and hexameric A*β*_42_ adopt a *β* strand motif driven by hydrophobic collapse of the hydrophobic core and C-terminus (Figs. 2 *B* and 3 *B*). The charge-dense, functional *β*E_31_ exhibits a broader landscape of hexameric conformations relative to A*β*_42_ hexamers (Fig. 3 *A*). The mutation of A*β*_42_ introducing charged and polar residues to the hydrophobic C-terminus destabilizes hexamer formation, inhibits hydrophobic packing (Fig. 4 *B*), and creates a broader free energy landscape for oligomeric conformations (Fig. 3 *A*). As such, we postulate that hydrophobic collapse is a determining factor in the continuum of amyloid functionality and cytotoxicity. An important limitation of this study to note is the scope; only including A*β*_42_ and *β*E_31_ does not consider the vast, diverse amyloidogenic landscape beyond these two peptides, ranging in cytotoxicity and functionality. Furthermore, amyloidogenic peptides can be both functional and cytotoxic, depending on aggregation environments, such as the hormone-peptide hIAPP (127). A larger-scale study including more peptides across the spectrum of functionality would be more robust for gleaning understanding of how sequence effects simulated oligomer structure dynamics and architectures. However, we believe that this work provides an argument to further probe the sequence effects that govern amyloid oligomer cytotoxicity.

## Acknowledgments

The authors thank Advanced Research Computing at Virginia Tech for access to computing resources. This work was supported by the 10.13039/100000001National Science Foundation CAREER Award (2237521) to A.M.B.: “CAREER: Resolving the Influence of Biologically Relevant Microenvironments on Amyloid Aggregation.”

## Author contributions

A.M.B. and K.M.K. designed the research protocol. K.M.K. and H.Z. performed simulations. K.M.K., H.Z., and A.M.B. analyzed data. K.M.K. and A.M.B. prepared the manuscript.

## Declaration of interests

The authors declare no competing interests.

## Supporting material

Document S1. Figures S1–S23 and Tables S1–S4

Document S2. Article plus supporting material
