# Supplementary material for: The role of hydrophobic collapse in cytotoxic and functional amyloid oligomerization
Source: Biophys J. 2025 Aug 6;124(18):2995–3007. doi: 10.1016/j.bpj.2025.07.042 (PMC12709261; doi:10.1016/j.bpj.2025.07.042)
Supplement: Document S1. Figures S1–S23 and Tables S1–S4 [file mmc1.pdf]

Biophysical Journal, Volume *124*

Supplemental information

The role of hydrophobic collapse in cytotoxic and functional amyloid oligomerization

Kelsie M. King, Hajar Zaheer, and Anne M. Brown

Table S1. Secondary structure percentages for Aβ_{42} and βE_{31} monomer simulations.

	Aβ_{42} Monomer Secondary Structure (%)			βE_{31} Monomer Secondary Structure (%)		
Replicate	***Coil***	***β-Sheet***	***Helix***	***Coil***	***β-Sheet***	***Helix***
Replicate 1	59.1	40.9	0.02	65.4	34.5	0.07
Replicate 2	55.8	44.1	0.02	61.4	38.5	0.06
Replicate 3	65.8	34.2	0.07	73.4	26.5	0.16
All	60.2	39.7	0.04	66.7	33.2	0.09

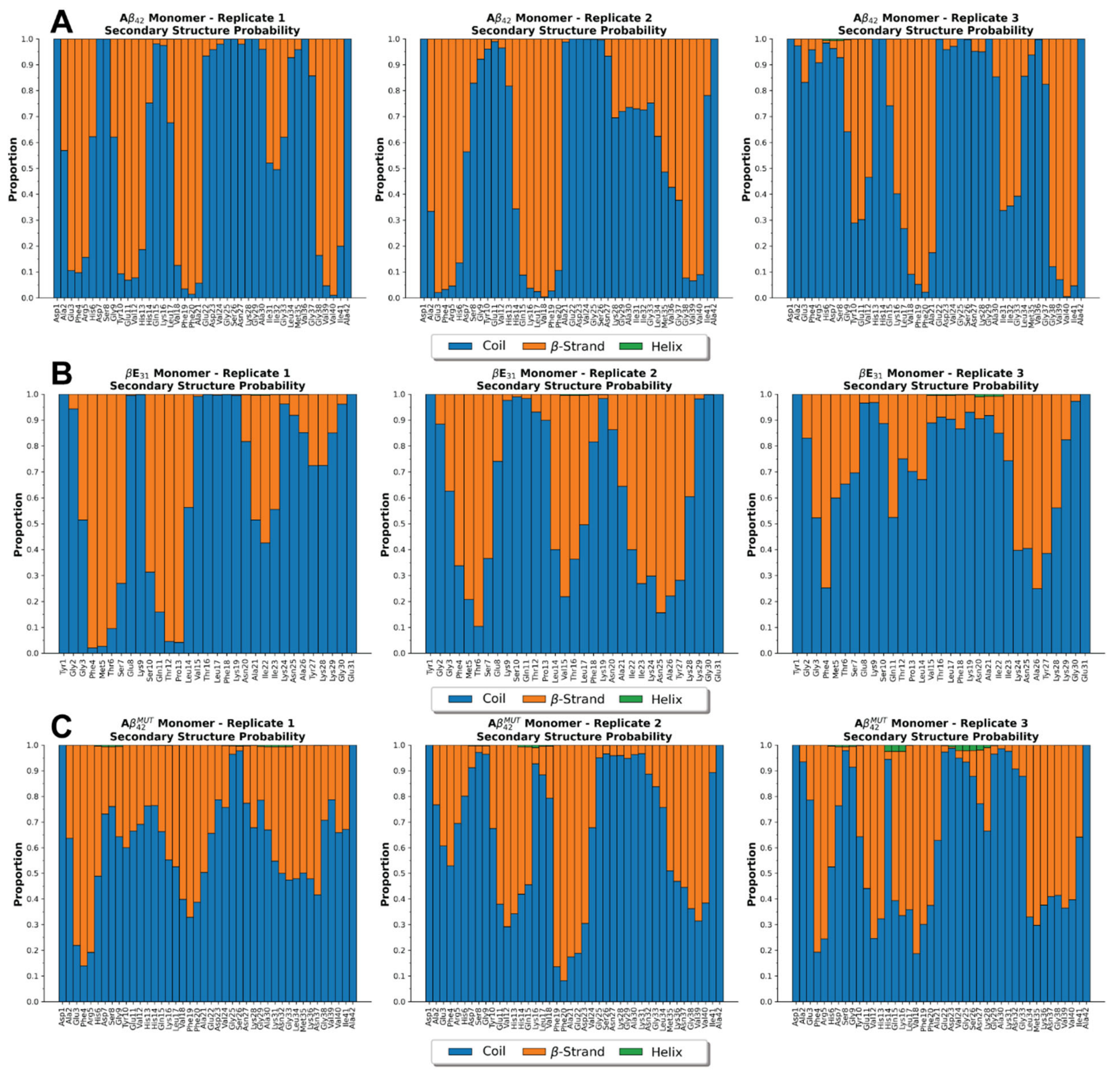

Figure S1. Secondary structure probability per residue from monomer simulations. Secondary structure probabilities for (**A**) Aβ_{42}, (**B**) βE_{31}, and (**C**) Aβ_{42}^{MUT}. Probabilities are colored as follows: coil (blue), β-strand (orange), helix (green). Probabilities calculated as percentage of frames a given residue adopts either β-strand, coil, or helical structure throughout the 2 μs simulation period for each replicate.

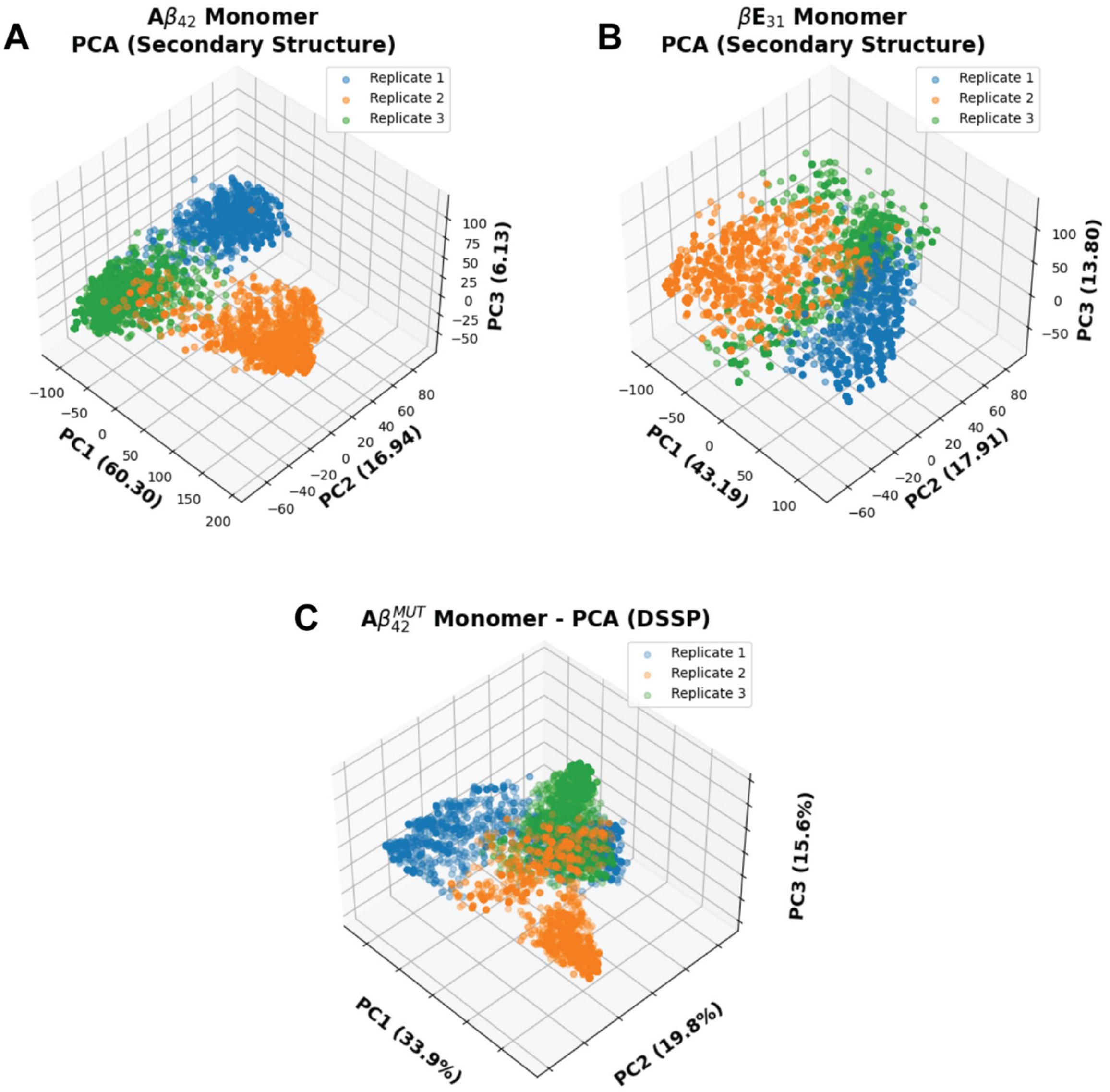

Figure S2. Principal-component analysis (PCA) of sampled secondary structure content from monomer simulations. DSSP data was down-sampled to include 2,000 frames/replicate (every 1 ns), for a total of 6,000 datapoints across all replicates, for (**A**) $A\beta_{42}$, (**B**) βE_{31}, and (**C**) $A\beta_{42}^{MUT}$.

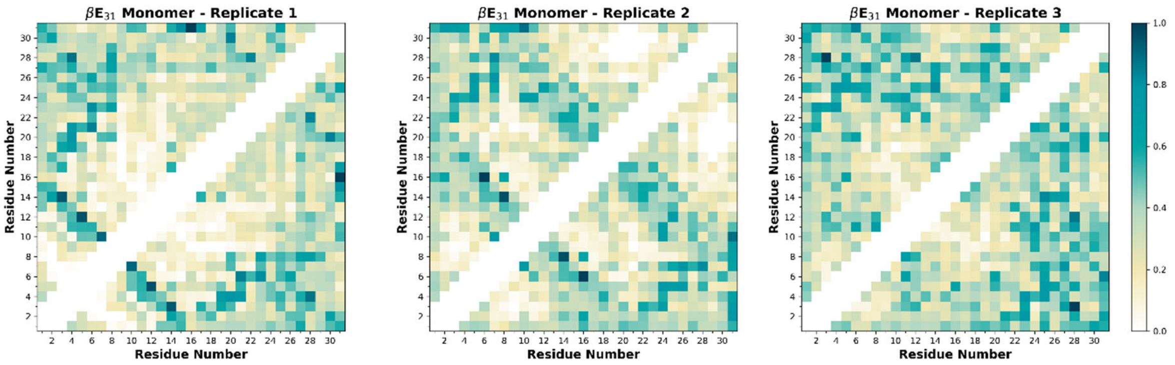

Figure S3. Intra-residue interaction probability heatmaps for βE$_{31}$ monomer simulations. Probabilities calculated based on residue pair distance and fraction of frames where pair distance ≤ 0.6 nm, scaled between 0 and 1.

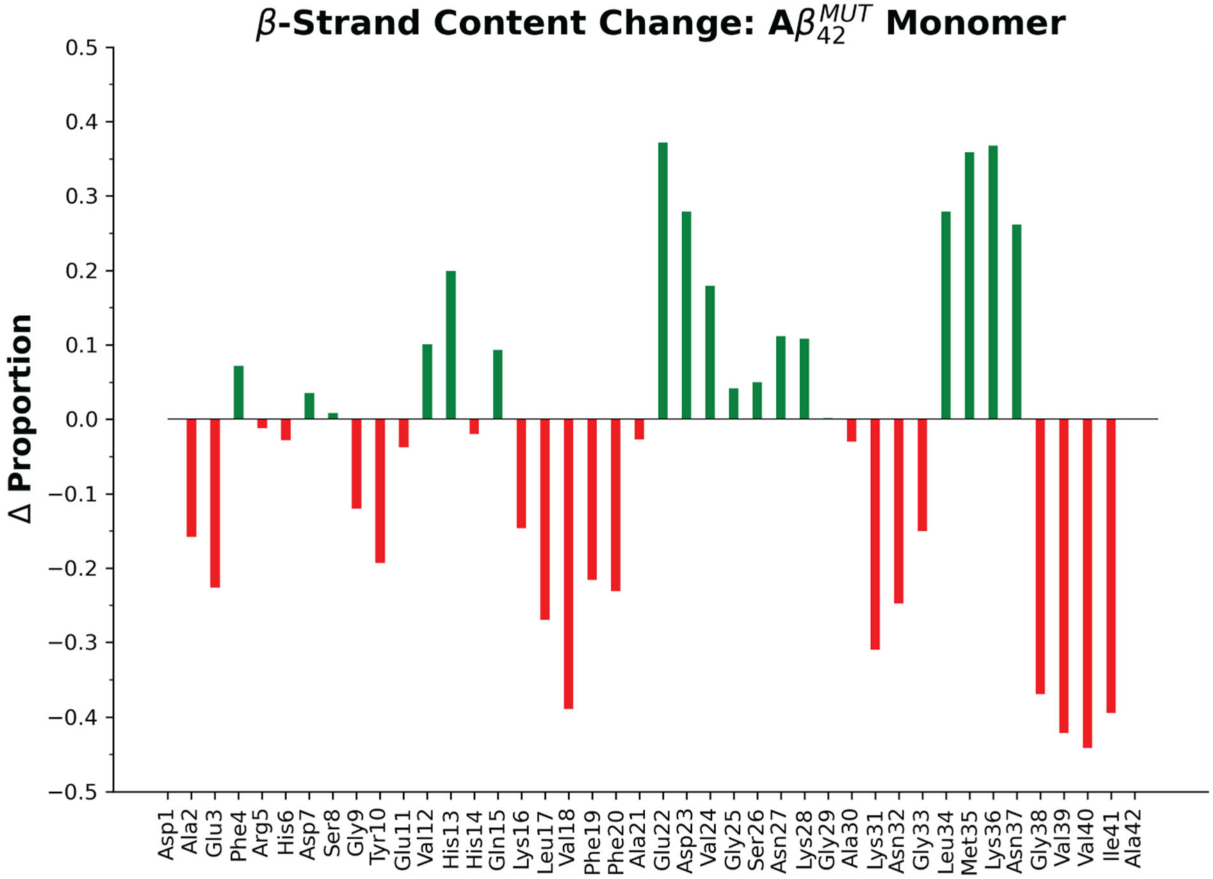

Figure S4. Change in β-strand content propensity from in monomeric $A\beta_{42}^{MUT}$ relative to monomeric $A\beta^{42}$. Green indicates an increase in β-strand probability in $A\beta_{42}^{MUT}$ relative to $A\beta_{42}$. red indicates a decrease in β-strand probability in $A\beta_{42}^{MUT}$ relative to $A\beta_{42}$. Initial probabilities calculated as percentage of frames a given residue adopts either β-strand, coil, or helical structure throughout the 2 μs simulation period over all replicates.

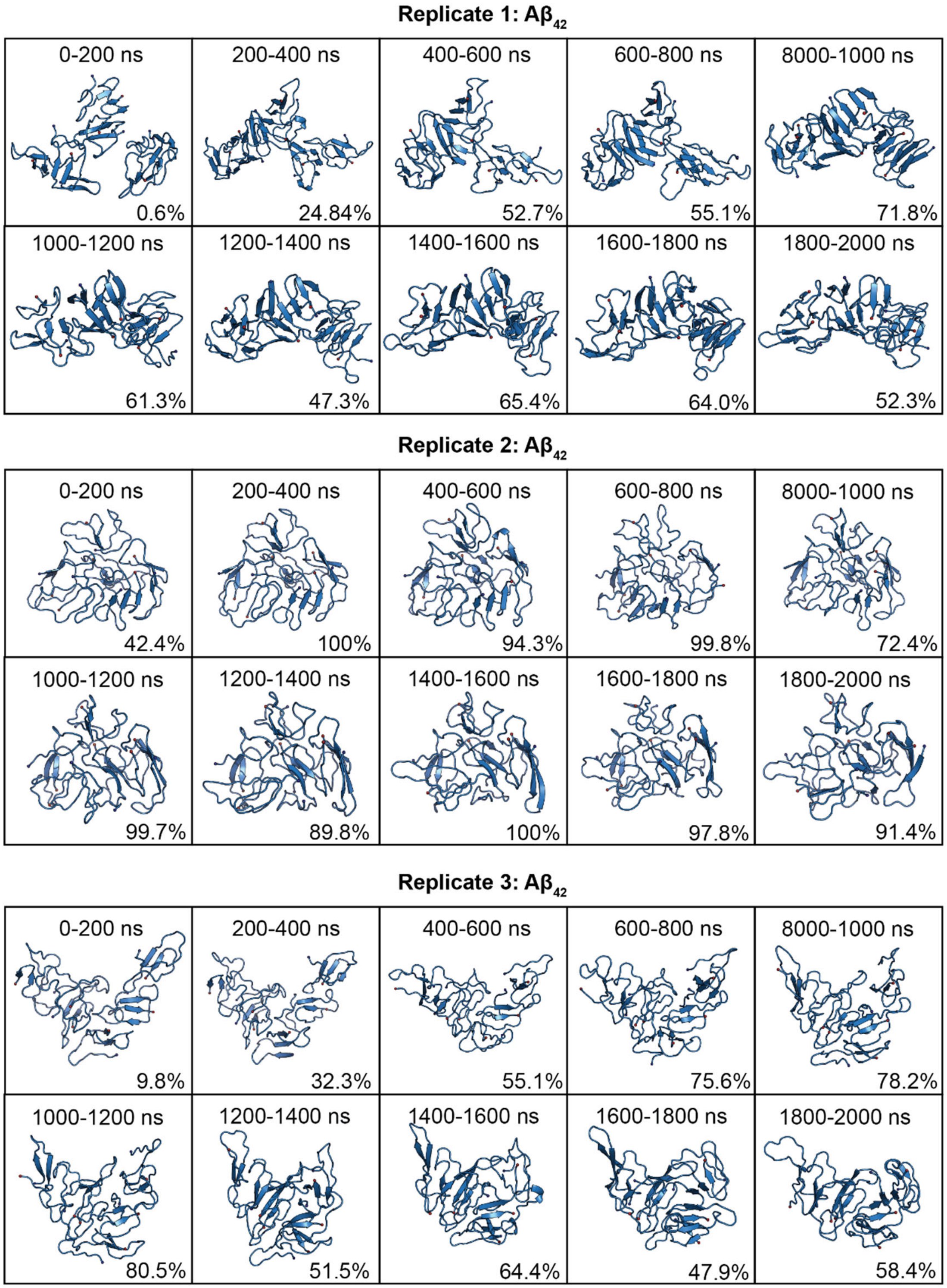

Figure S5. Representative structures of $A\beta_{42}$ hexamers from RMSD clustering. RMSD clustering was performed over 200 ns intervals of the trajectory. Protein shown as blue cartoon. Percentages indicate the percentage of frames the structure represents over the indicated timeframe.

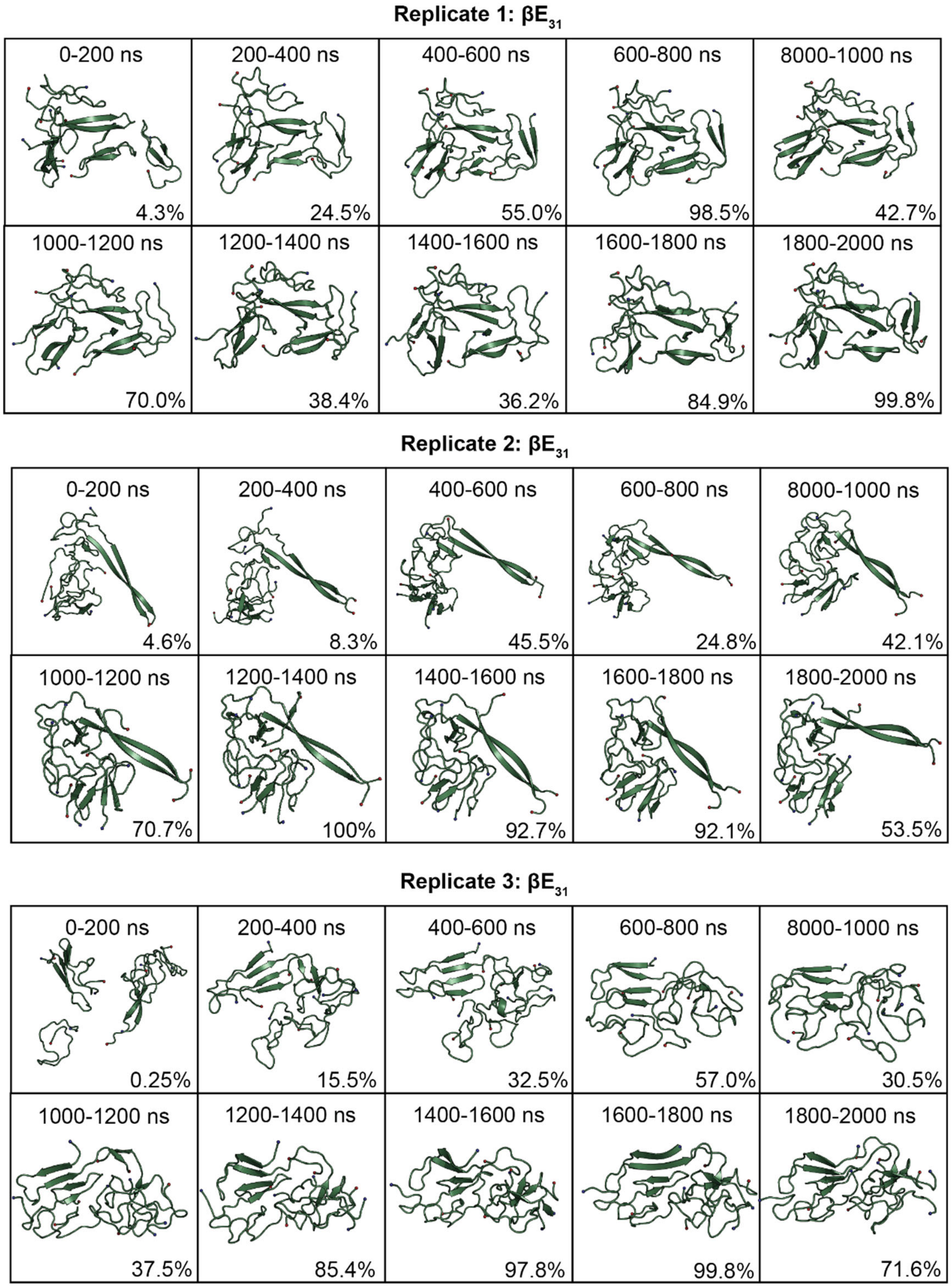

Figure S6. Representative structures of βE_{31} hexamers from RMSD clustering. RMSD clustering was performed over 200 ns intervals of the trajectory. Protein shown as green cartoon. Percentages indicate the percentage of frames the structure represents over the indicated timeframe.

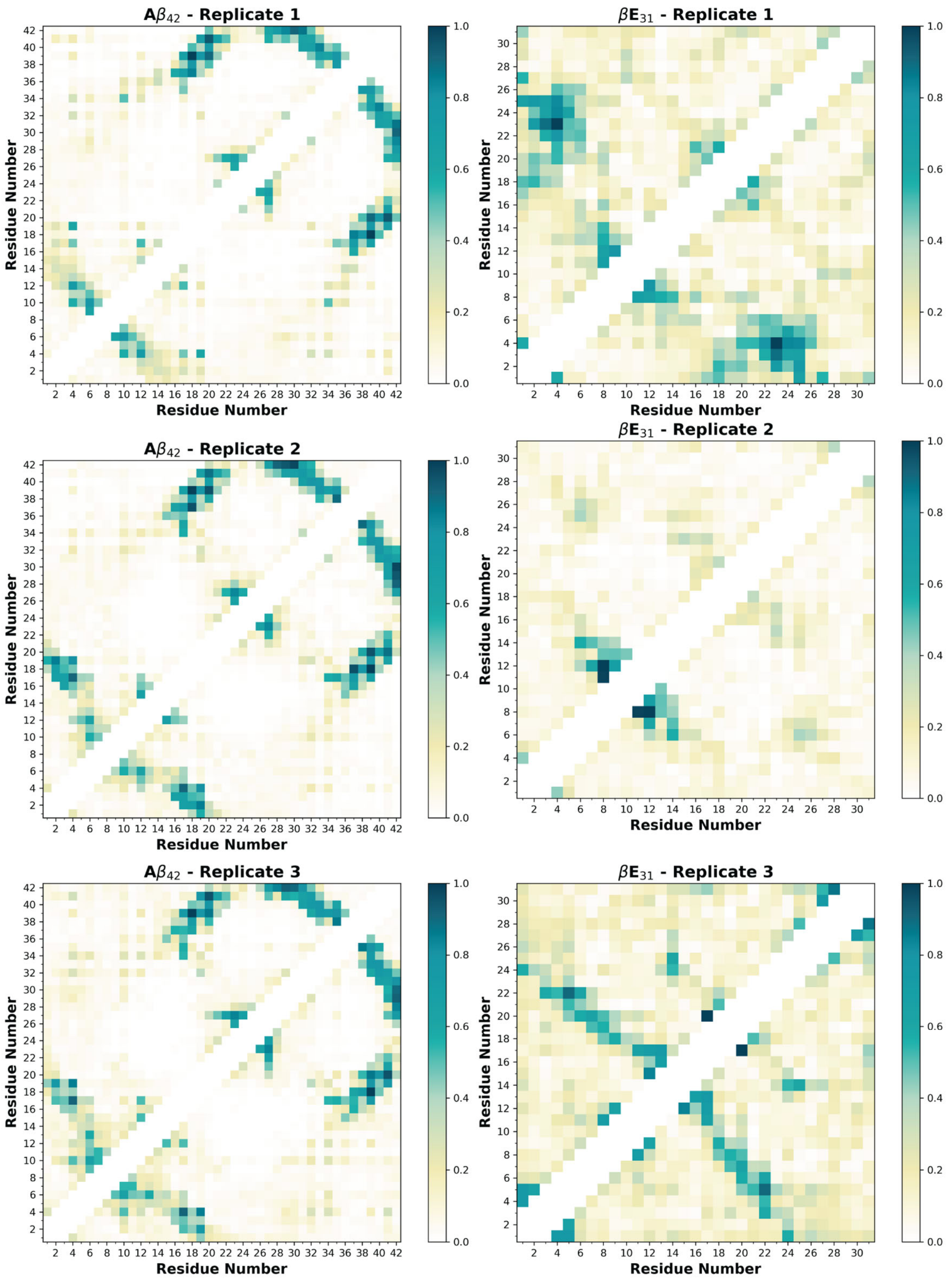

Figure S7. Intra-molecular interaction probability heatmaps by replicate Aβ42 and βE31 hexamer simulations. Represents weighted frequency of interaction over the 2 μs simulation period for residues on the same chain. Residue pairs within 2 residues are excluded.

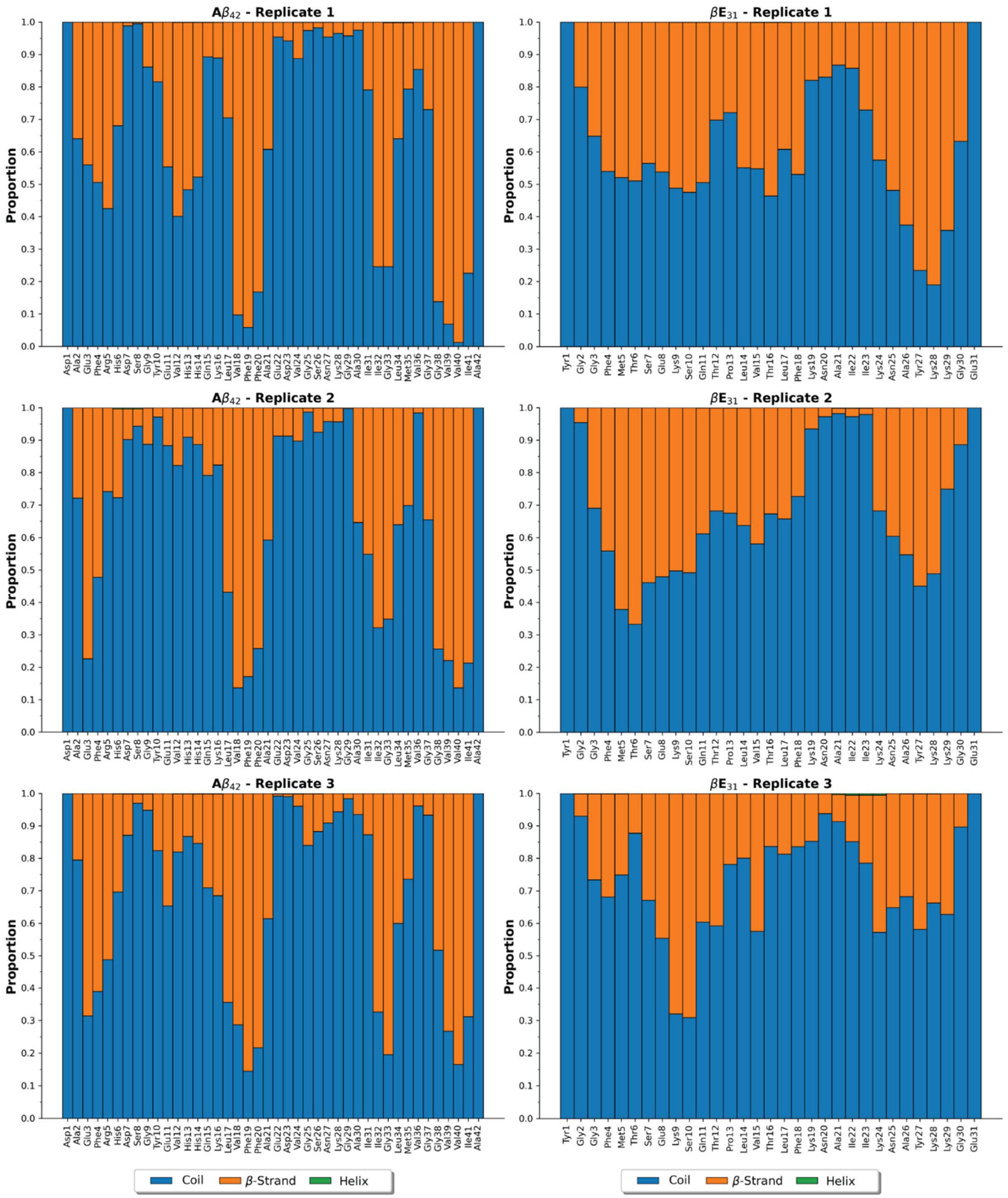

Figure S8. Secondary structure probability per residue from hexamer simulations. Secondary structure probabilities for (**left**) $A\beta_{42}$ and (**right**) βE_{31}. Probabilities are colored as follows: coil (blue), β-strand (orange), helix (green). Probabilities calculated as percentage of frames a given residue adopts either β-strand, coil, or helical structure throughout the 2 μs simulation period for each replicate

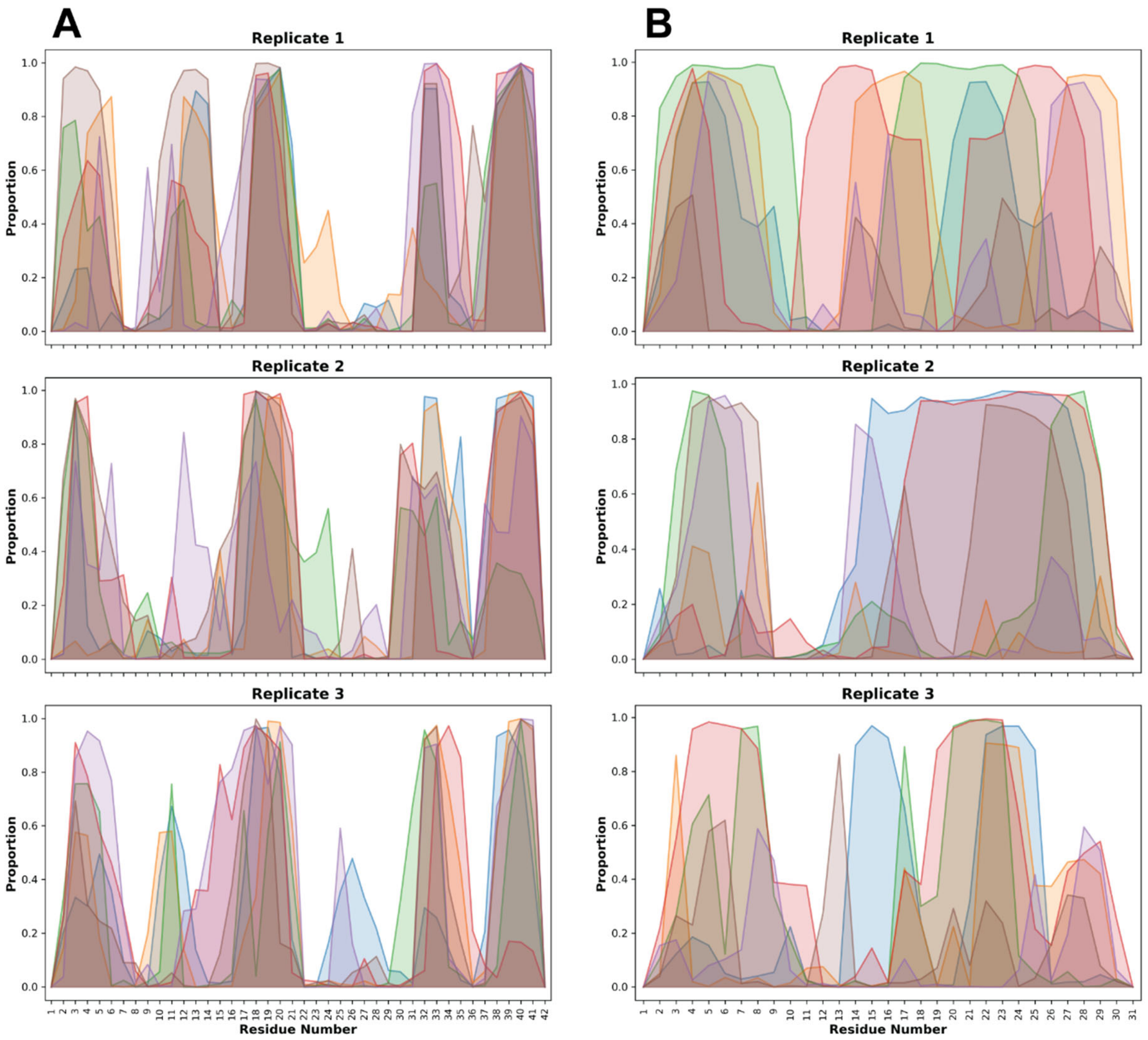

Figure S9. Proportion of frames β-strand is sampled per-residue and per-chain, for (A) Aβ$_{42}$ and (B) βE$_{31}$ hexamer simulations.

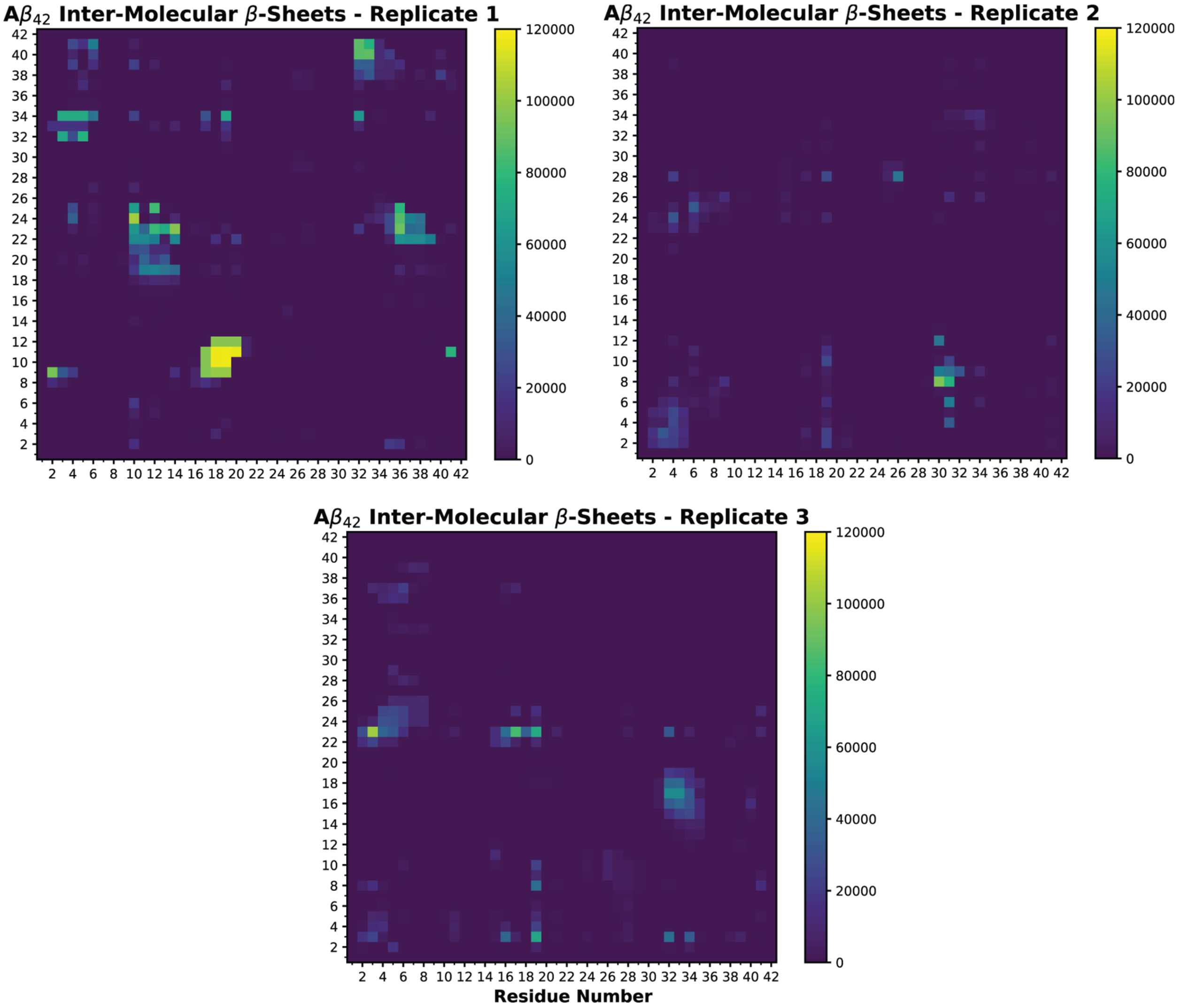

Figure S10. Intermolecular β-strand counts per residue for Aβ42 hexamer simulations. For each frame (downsampled every 10 ps), and for each residue pair, if an interaction was present ($d \leq 0.6$ nm), and both residues adopted β-strand content at that frame, the count was increased.

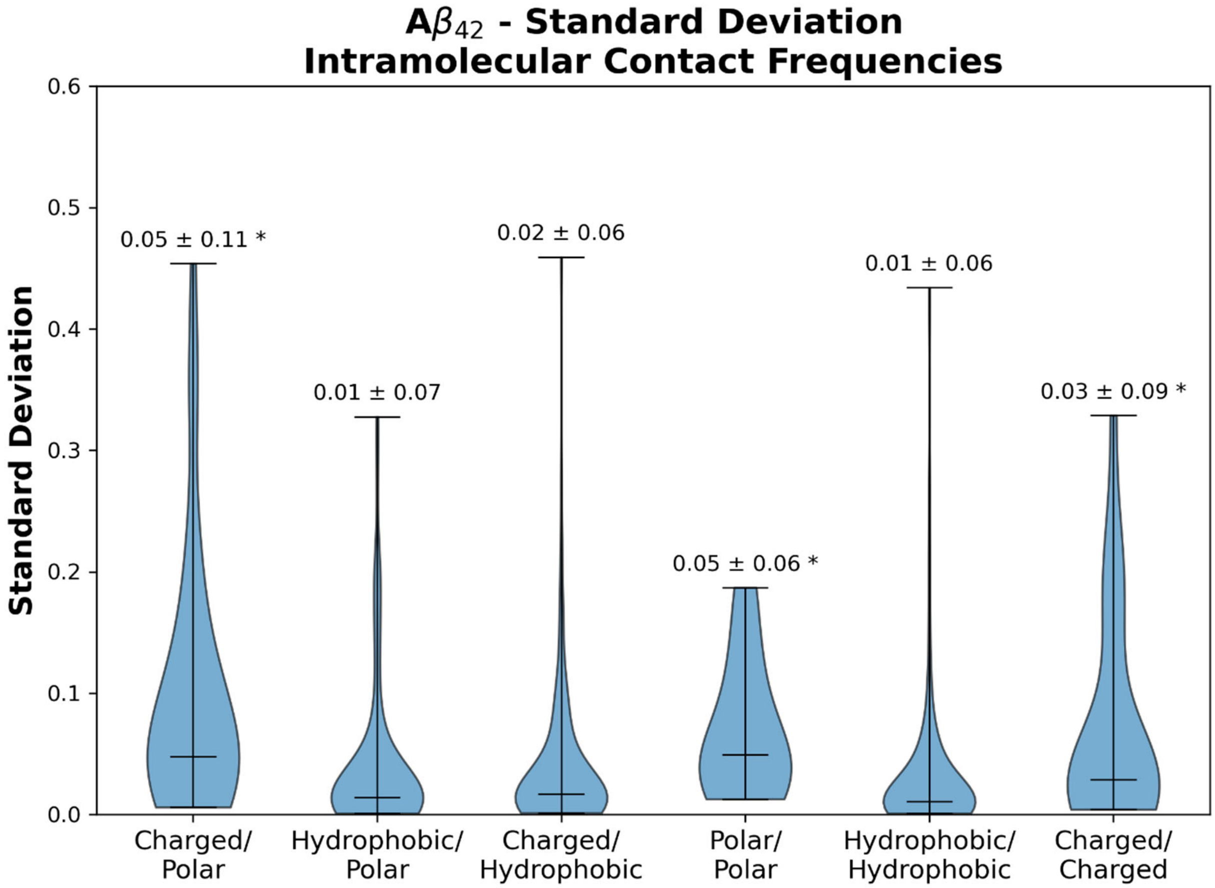

Figure S11. Standard deviations of intramolecular contact frequencies for Aβ$_{42}$ by residue sidechain property. Asterisk (*) indicates statistically significant difference in mean to other groups ($p \leq 0.5$).

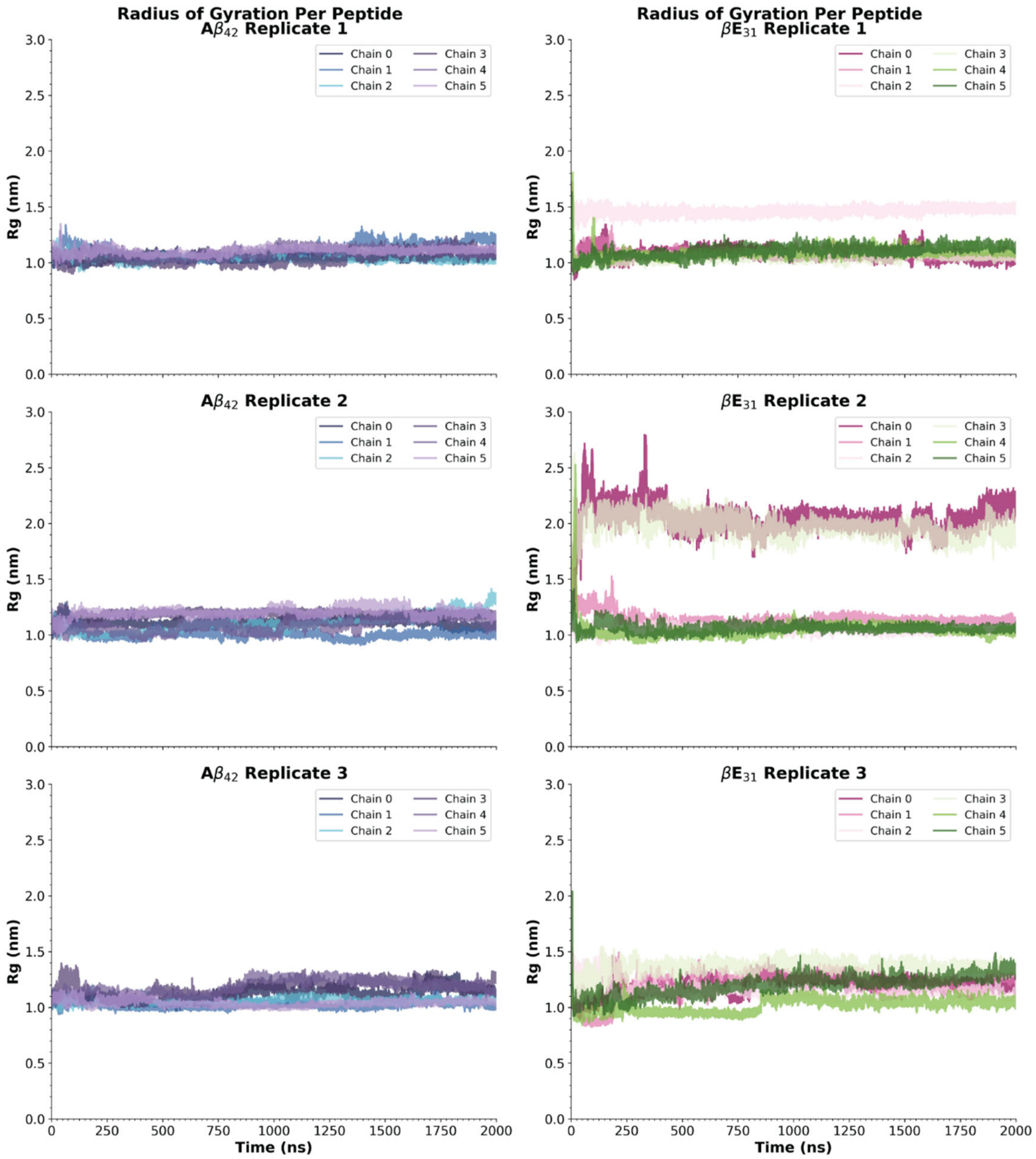

Figure S12. Radius of gyration over time per peptide for Aβ_{42} and βE_{31} hexamers.

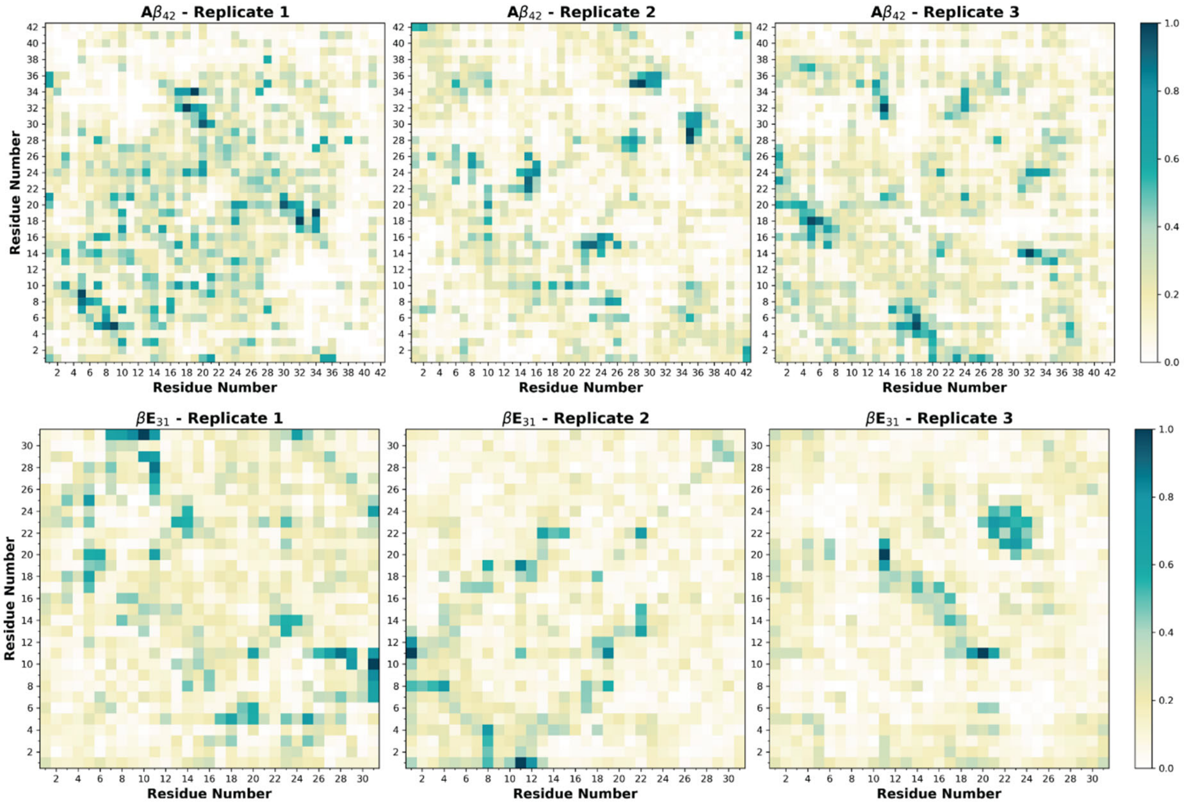

Figure S13. Inter-molecular interaction probability heatmaps by replicate Aβ_{42} and βE_{31} hexamer simulations. Represents weighted frequency of interaction over the 2 μs simulation period for residues that are not located on the same chain.

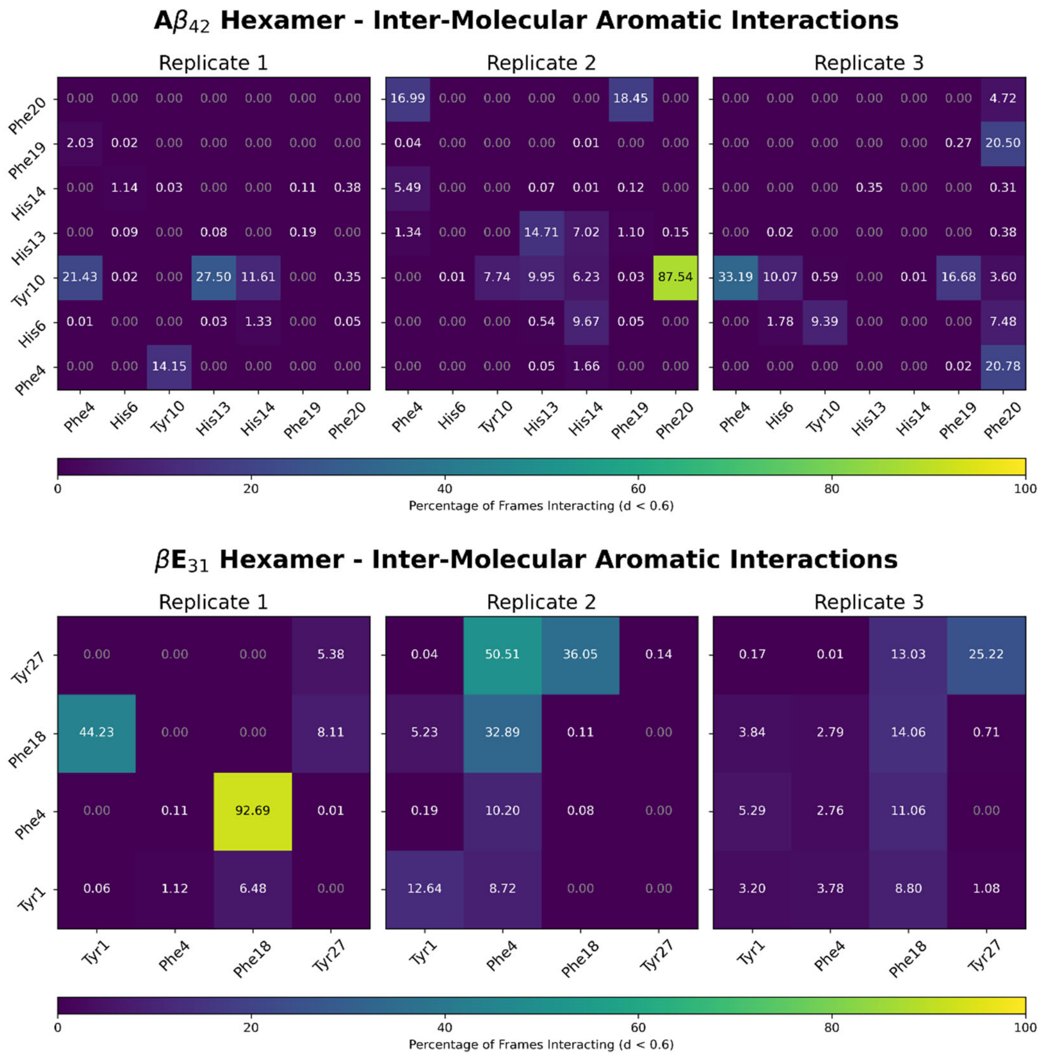

Figure S14. Inter-molecular π-stacking interaction heatmaps by replicate for (top) $A\beta_{42}$ and (bottom) βE_{31} hexamer simulations. Represents the maximum percentage of interaction between aromatic residues not located on the same chain.

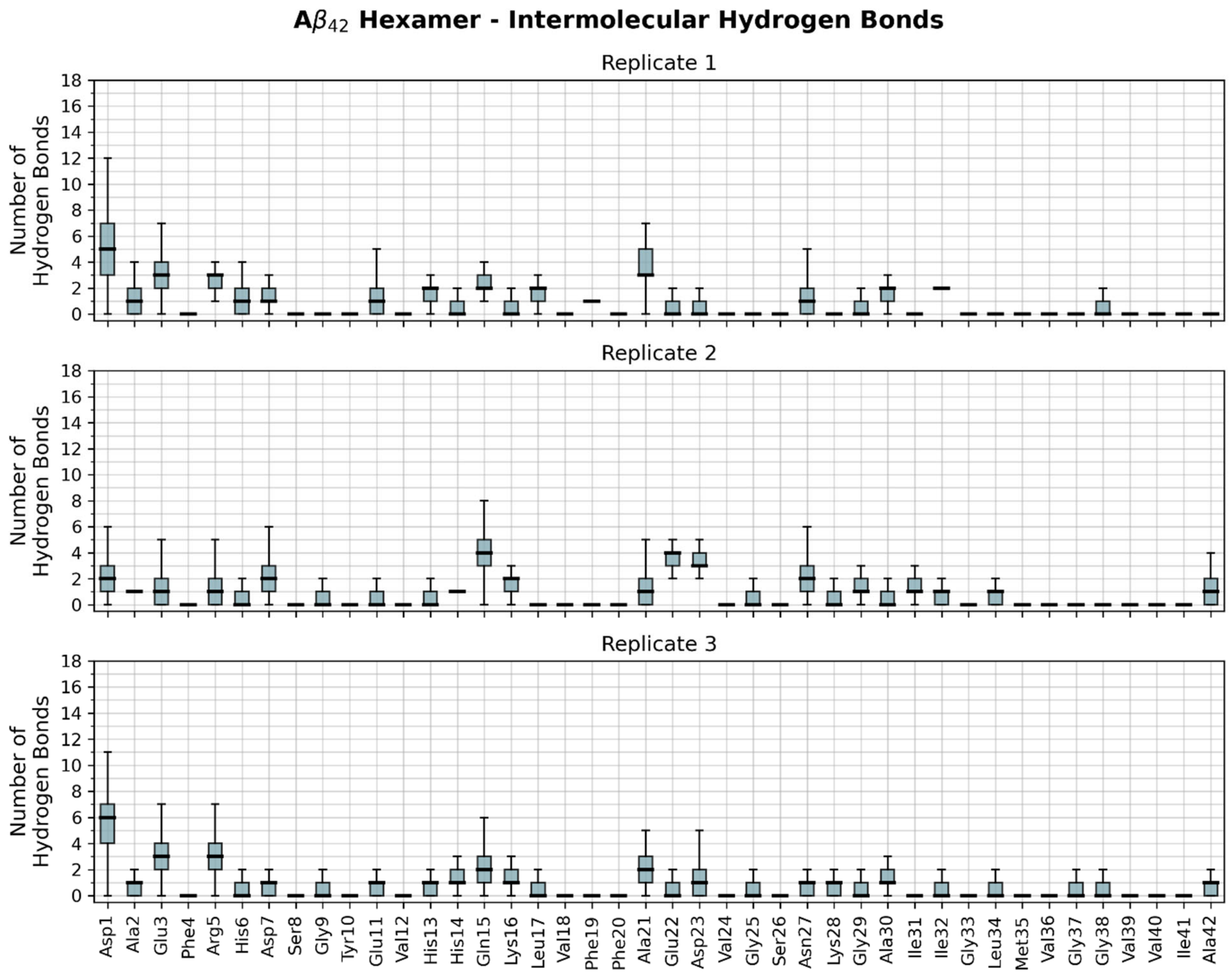

Figure S15. Inter-molecular hydrogen bonds by replicate for Aβ42 hexamer simulations. Boxplots represent the number of hydrogens bonds each residue participates in over the 2 μs simulation period. Outliers not shown for clarity.

Table S2. Inter-molecular salt bridge interactions present in Aβ_{42} hexamer simulations.

Replicate	*Negative Residue*	*Positive Residue*	*Occupancy (%, from 0.3–2 μs)*
**1**	Asp7	Asp1 (N-term)	33.3
	Asp1	Asp1 (N-term)	32.8
**2**	Asp1	Lys28	38.5
	Ala42 (C-term)	Lys16	33.5
	Glu3	Asp1 (N-term)	15.4
	Asp7	Asp1 (N-term)	11.9
	Glu22	Asp1 (N-term)	10.2
**3**	Asp23	Asp1 (N-term)	74.3
	Asp1	Lys16	34.3
	Glu22	Asp1 (N-term)	17.8
	Glu22	Lys16	14.3
	Asp7	Lys16	12.1

Salt bridge interaction was defined as a distance ≤ 0.3 between charged atoms of charged residues not located on the same chain. Occupancy calculated as a percentage of frames present in the aggregated period of simulation (0.3-2 μs). Only salt bridges present for ≥10% of the sampled period are shown.

Table S3. Inter-molecular salt bridge interactions present in βE_{31} hexamer simulations.

Replicate	*Negative Residue*	*Positive Residue*	*Occupancy (%, from 0.3–2 μs)*
**1**	Glu31	Lys9	12.75, 16.5
	Glu31	Lys24	14.8
	Glu31	Lys28	10.7
	Glu8	Lys9	10.0
**2**	Glu8	Lys19	34.0, 44.2
	Glu31	Lys28	25.8
	Glu31	Lys29	19.2
**3**	Glu8	Lys19	19.8
	Glu31	Lys28	19.6
	Glu31	Lys24	16.5

Salt bridge interaction was defined as a distance ≤ 0.3 between charged atoms of charged residues not located on the same chain. Occupancy calculated as a percentage of frames present in the aggregated period of simulation (0.3-2 μs). Only salt bridges present for ≥10% of the sampled period are shown.

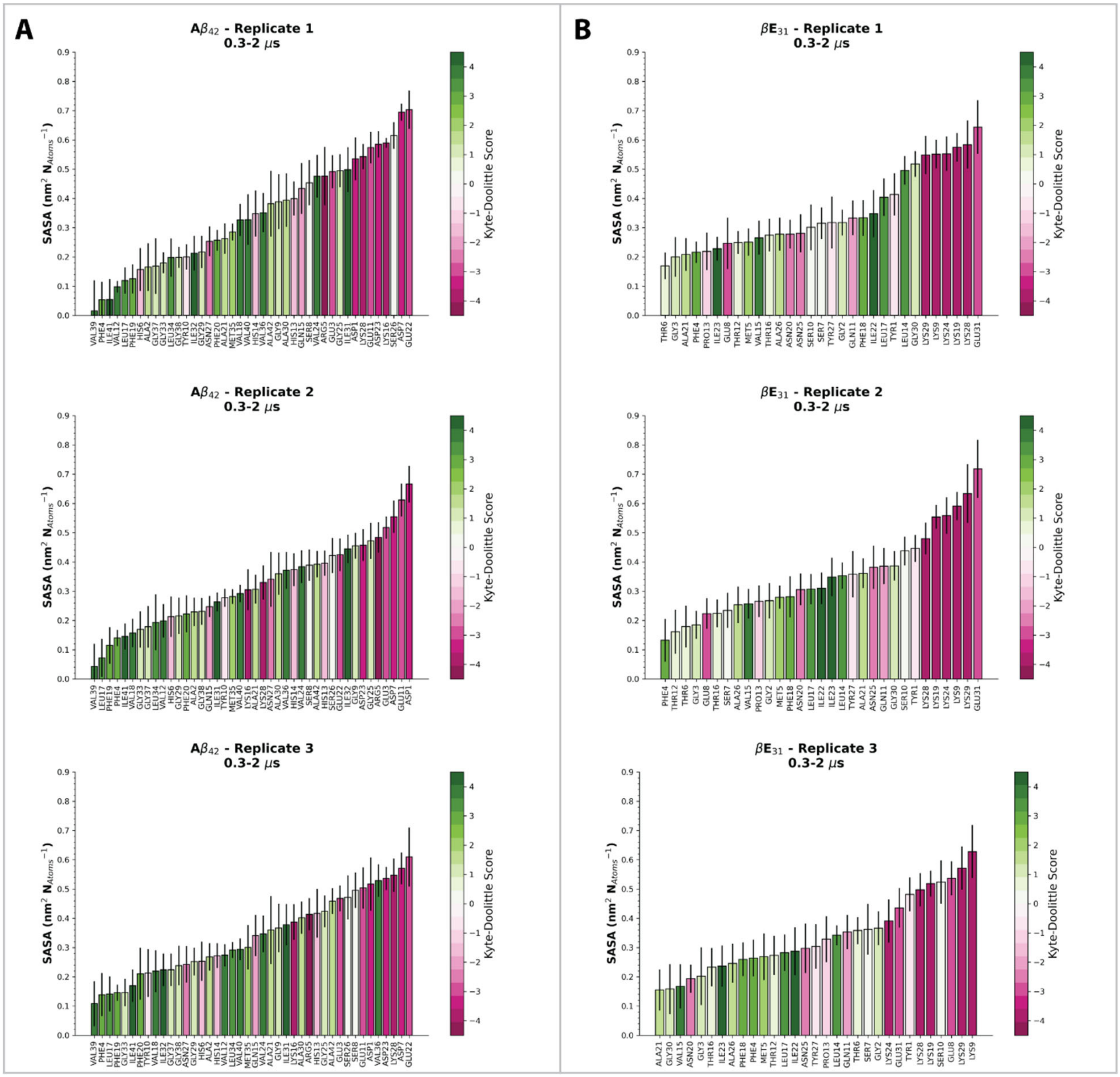

Figure S16. Inter-residue interaction occupancies for (A) Aβ_{42} and (B) βE_{31} hexamers. Distances between every possible residue pair were calculated and occupancy was defined as the fraction of frames a residue pair was within 0.6 nm cutoff. Left shows interaction occupancies greater than 10% of simulation time. This is shown separately to highlight differences, as low occupancies are common (right).

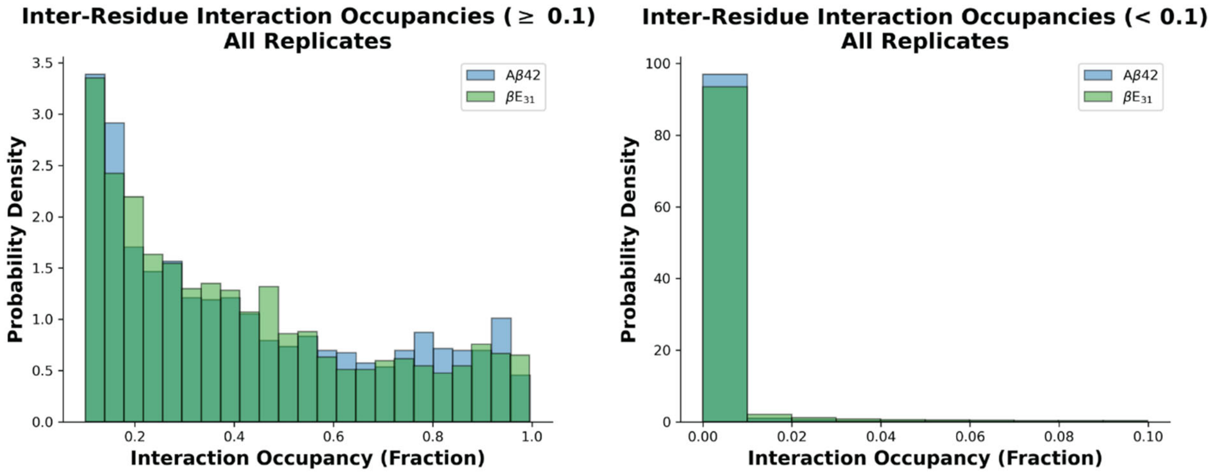

Figure S17. Inter-residue interaction occupancies for Aβ_{42} and βE_{31}. Distances between every possible residue pair were calculated and occupancy was defined as the fraction of frames a residue pair was within 0.6 nm cutoff. Left shows interaction occupancies greater than 10% of simulation time. This is shown separately to highlight differences, as low occupancies are common (right).

Table S4. The mean of pairwise Hamming distances of secondary structure content of residues 28-42 in $A\beta_{42}$ and $A\beta_{42}^{MUT}$ hexamer simulations.

	**$A\beta_{42}$**	**$A\beta_{42}^{MUT}$**
Replicate 1	2.7 ± 1.5	5.9 ± 3.0
Replicate 2	4.7 ± 2.6	4.1 ± 2.2
Replicate 3	2.7 ± 1.5	4.2 ± 2.4
**Mean**	**3.4 ± 1.9**	**4.7 ± 2.5**

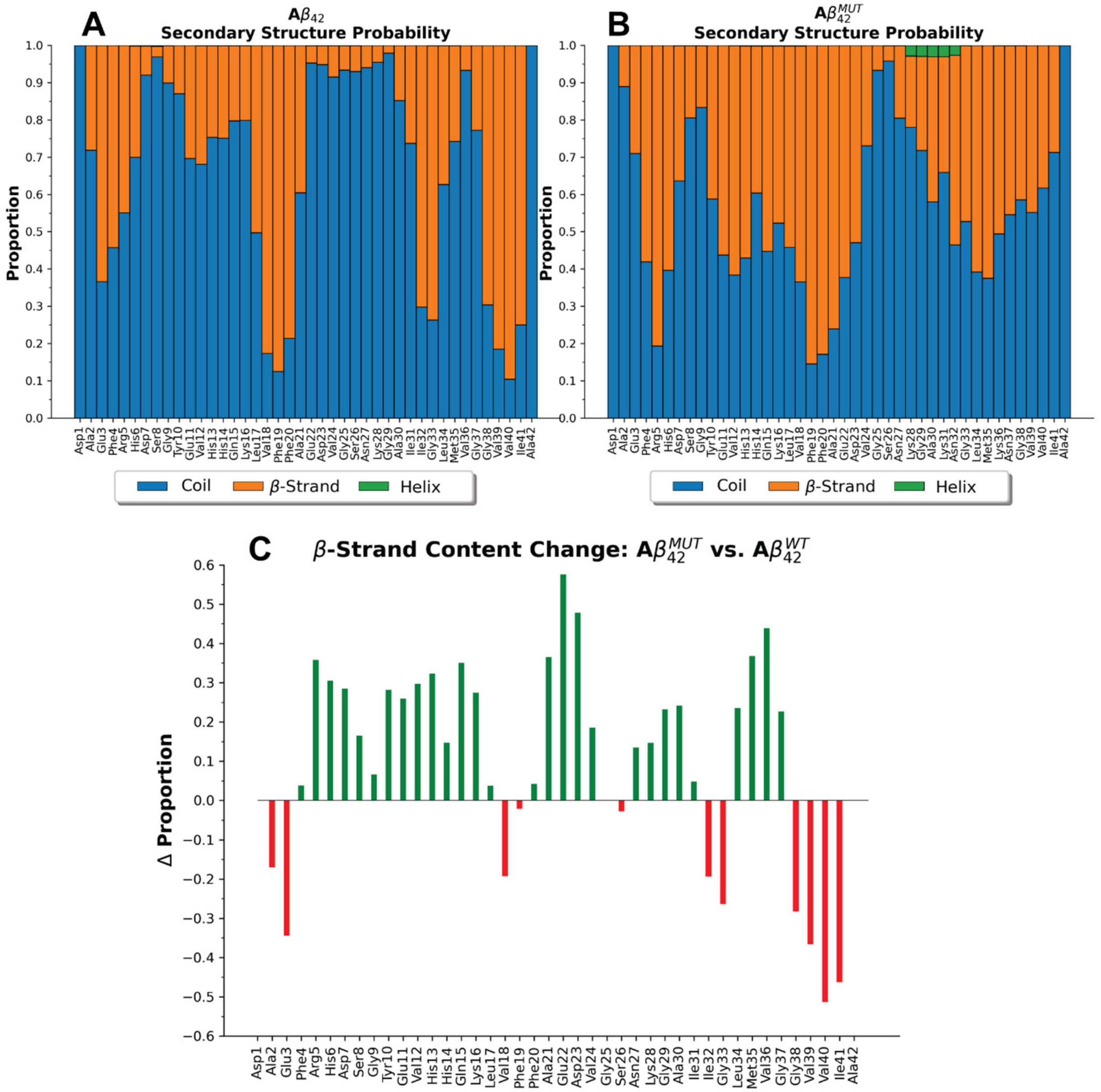

Figure S18. Secondary structure probabilities and changes for $A\beta_{42}$ and $A\beta_{42}^{MUT}$. Secondary structure probabilities over all replicates and over the entire 2 μs simulation for **(A)** $A\beta_{42}$ and **(B)** $A\beta_{42}^{MUT}$ hexamers. **(C)** Change in β-strand content propensity from in hexameric $A\beta_{42}^{MUT}$ relative to hexameric $A\beta_{42}$. Green indicates an increase in β-strand probability in hexameric $A\beta_{42}^{MUT}$ relative to hexameric $A\beta_{42}$, and red indicates a decrease.

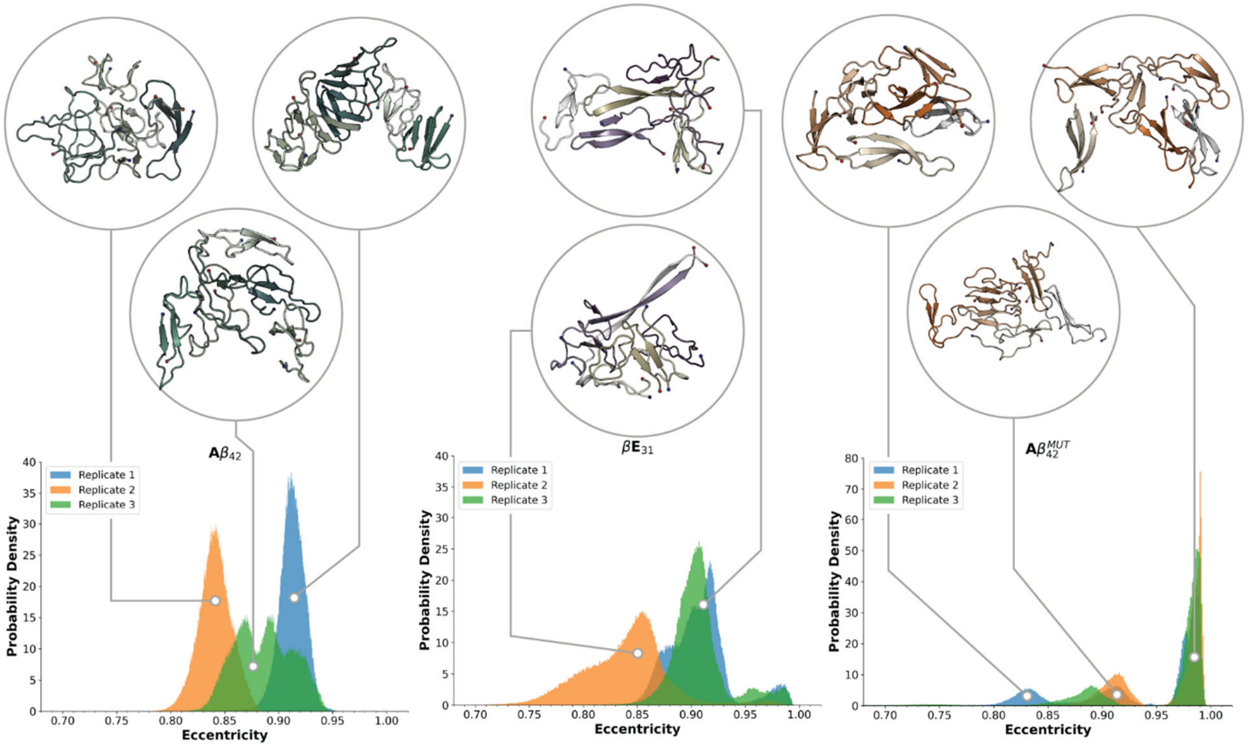

Figure S19. Distributions of eccentricity for (left) Aβ42, (middle) βE31, and (right) Aβ42^{MUT} hexamer simulations. Distributions represent eccentricity values sampled in the aggregated simulation period (0.3-2 μs) Snapshots show cluster structures corresponding to the area of the distribution.

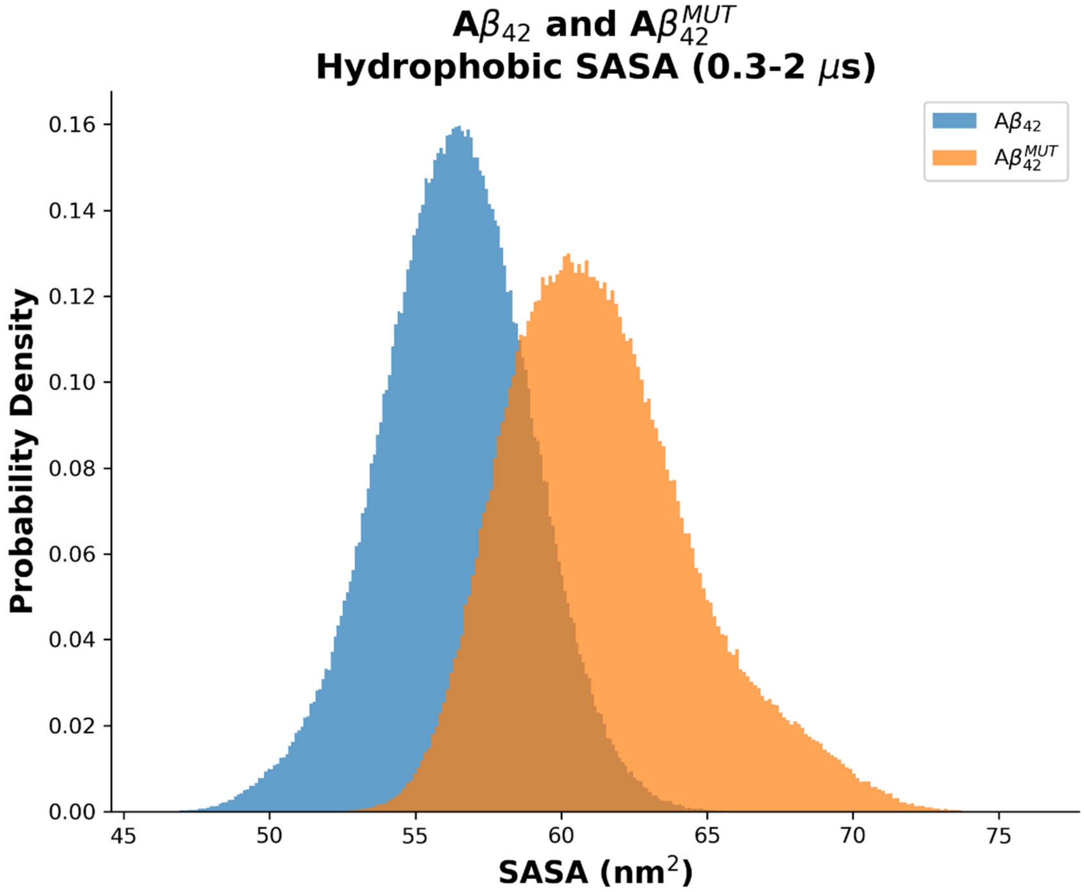

Figure 20. Distribution of hydrophobic solvent-accessible surface area (SASA) sampled for $A\beta_{42}$ and $A\beta_{42}^{MUT}$ hexamers. Distributions show SASA over the aggregated simulation period (0.3-2 μs). Distributions include data from all replicates.

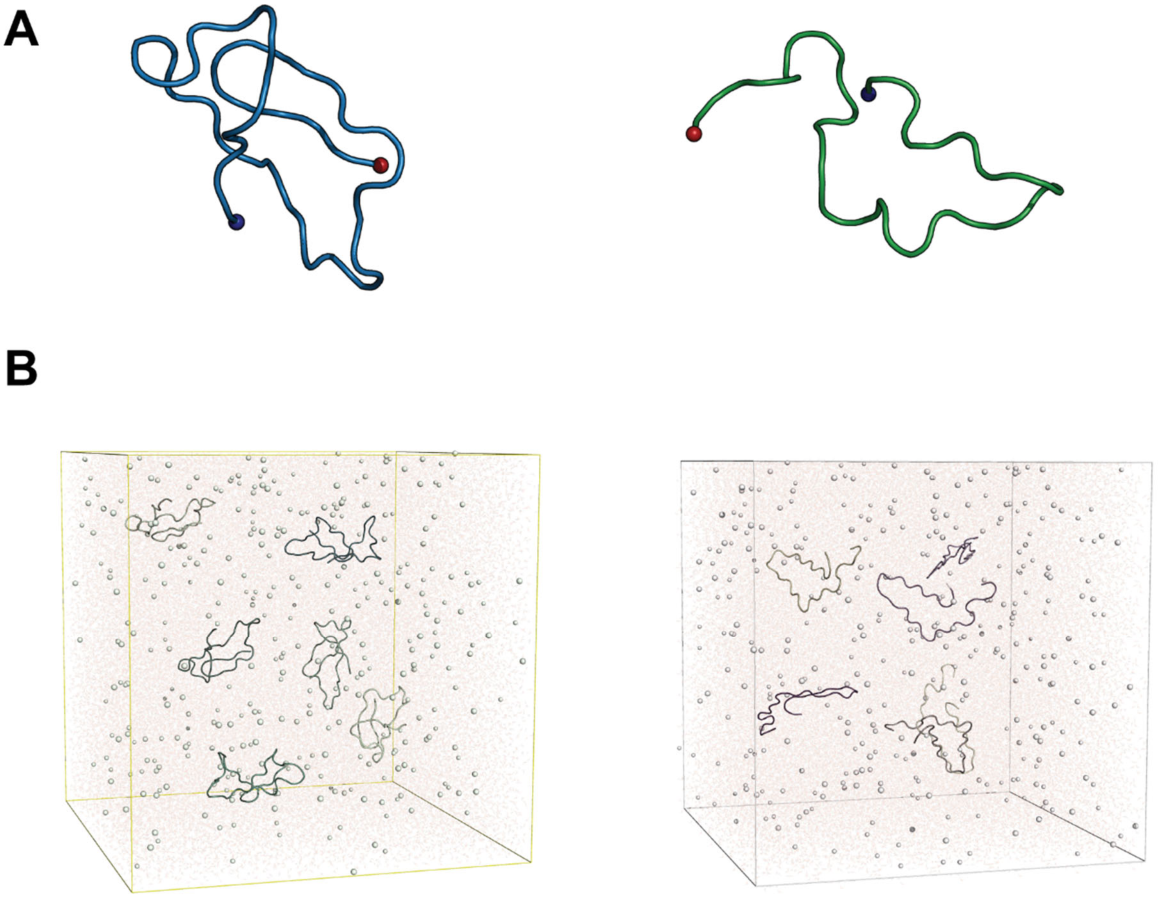

Figure S21. Starting structures for Aβ$_{42}$ and βE$_{31}$ simulations. (A) monomeric simulation starting structures, **(B)** hexameric simulation starting structures.

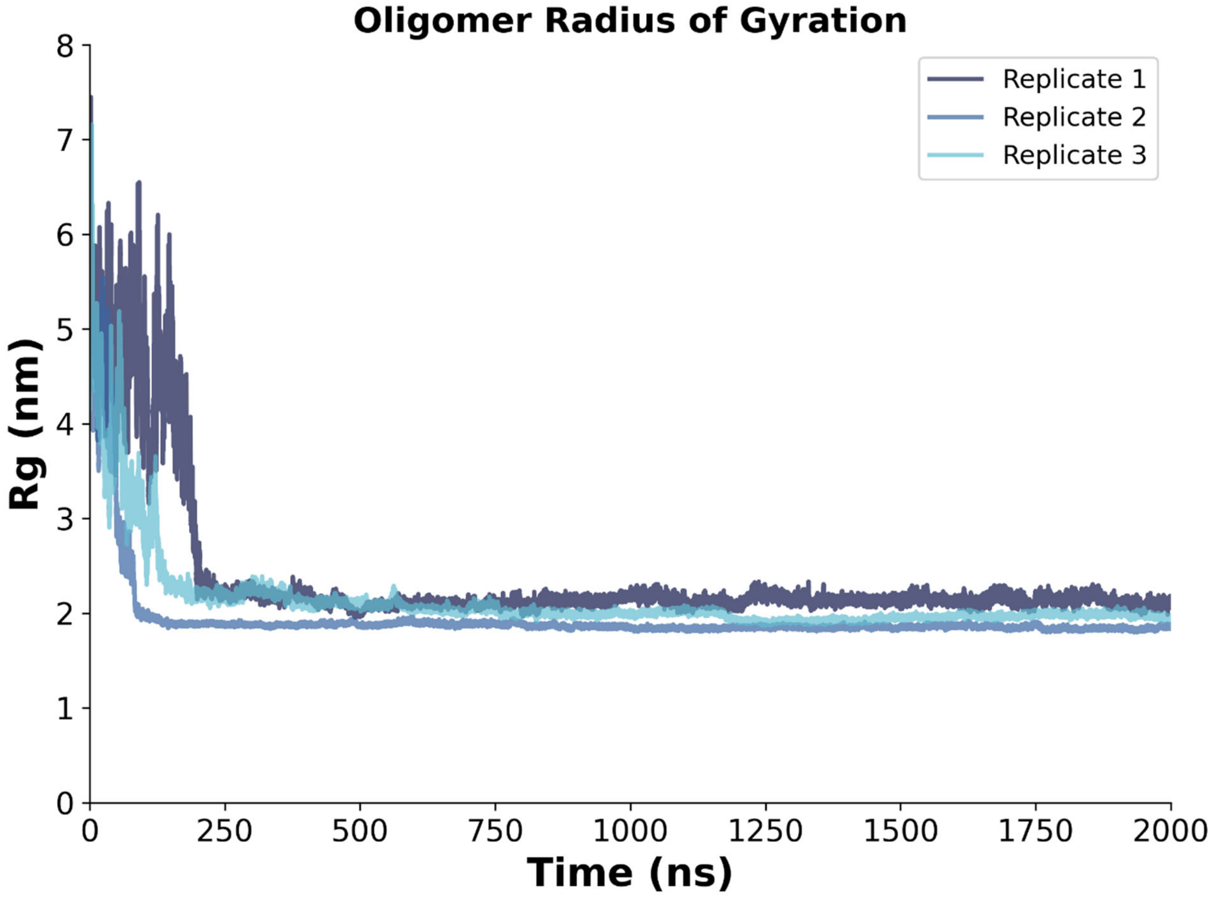

Figure S22. Radius of gyration over time for the Aβ$_{42}$ hexamer, by replicate.

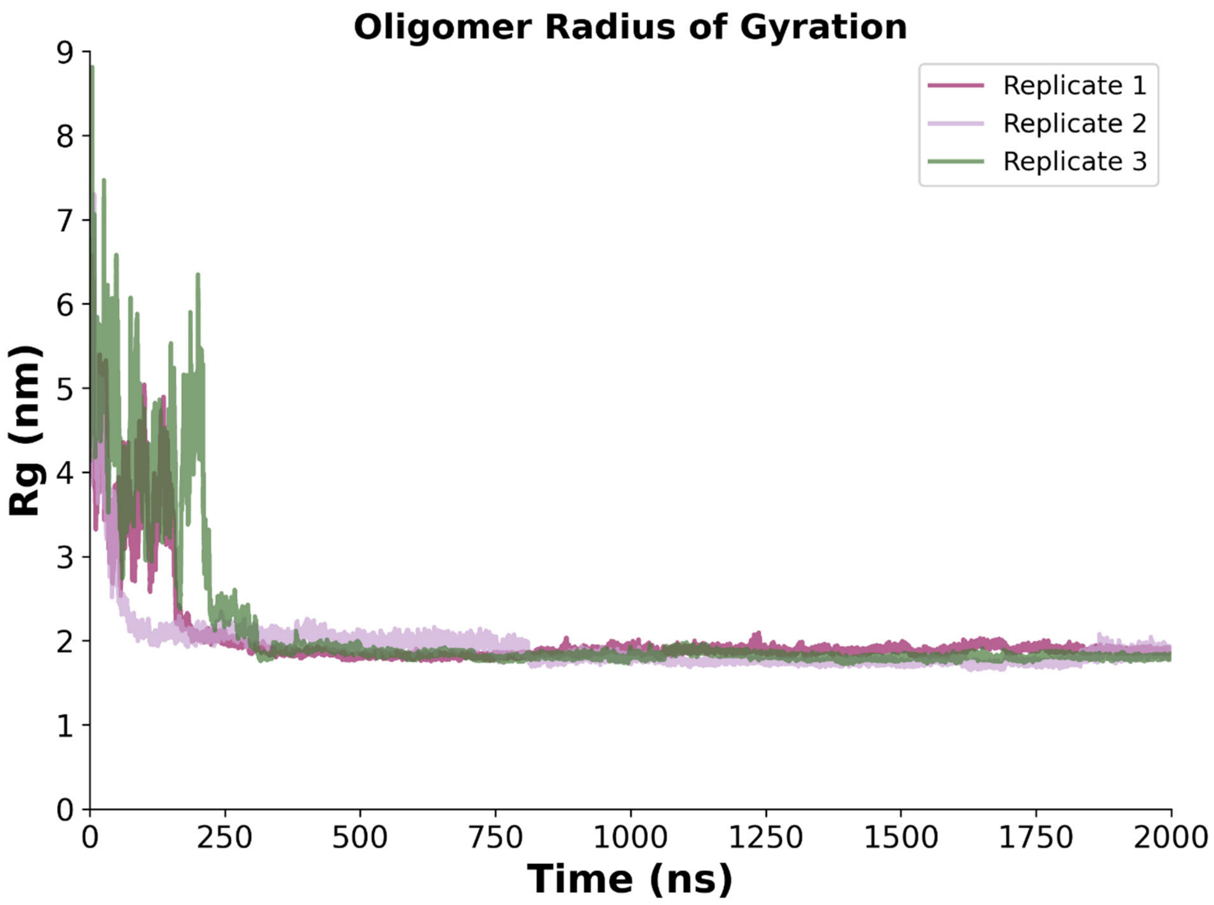

Figure S23. Radius of gyration over time for the βE_{31} hexamer, by replicate.
