# Supplementary material for: The role of hydrophobic collapse in cytotoxic and functional amyloid oligomerization
Source: Biophys J. 2025 Aug 6;124(18):2995–3007. doi: 10.1016/j.bpj.2025.07.042 (PMC12709261; doi:10.1016/j.bpj.2025.07.042)
Supplement: Document S2. Article plus supporting material [file mmc2.pdf]

Biophysical Journal
Article

Biophysical Society

The role of hydrophobic collapse in cytotoxic and functional amyloid oligomerization

Kelsie M. King,[1] Hajar Zaheer,[2] and Anne M. Brown[1,2,3,4,5,*]
[1]Department in Genetics, Bioinformatics, and Computational Biology, Virginia Tech, Blacksburg, Virginia; [2]Department of Biochemistry, Virginia Tech, Blacksburg, Virginia; [3]Data Services, University Libraries, Virginia Tech, Blacksburg, Virginia; [4]Virginia Tech Center for Drug Discovery, Virginia Tech, Blacksburg, Virginia; and [5]Academy of Integrated Science, Virginia Tech, Blacksburg, Virginia

ABSTRACT "Amyloid" refers to an insoluble, highly organized protein fibril composed of intermolecular β sheets, known as a cross-β motif. Amyloidogenic proteins are generally driven to aggregate into tightly packed fibrils. Some amyloids are functional, often being utilized as hormone storage reservoirs. The functional, paracrine signaling neuropeptide β-endorphin (βE) is stored and released to modulate pain responses. Conversely, the function of amyloid-β (Aβ), involved in Alzheimer's disease, is uncertain—but substantial evidence exists of its role in neuronal cell apoptosis. Although both peptides are mechanistically linked in their propensity to adopt fibrillar structures, the biophysical characteristics that drive divergence in cytotoxic potential are not well understood. To probe the conformational dynamics and mechanisms of functional and cytotoxic oligomer formation, we utilized all-atom molecular dynamics to simulate the formation of monomeric and hexameric Aβ_{42} and βE$_{31}$. Monomeric Aβ_{42} and βE$_{31}$ selectively sampled β strand motifs comprising hydrophobic residues, adopting a collapsed state. Cluster analysis indicates that βE$_{31}$ hexamers were more conformationally diverse than those sampled by Aβ_{42}, suggesting that βE$_{31}$ exhibits more signatures of disorder. Aβ_{42} hexamer formation was driven by hydrophobic packing of collapsed β strand motifs, where βE$_{31}$ hexamer peptide subunits remained structurally plastic and solvent accessible. Mutation of Aβ_{42} disrupting the C-terminal hydrophobic sequence inhibited hydrophobic β strand formation, reduced aggregation propensity, and increased solvent accessibility, suggesting that retention of a collapsed state is critical for aberrant oligomer formation. This work provides a preliminary view of cytotoxic and functional oligomer morphologies at atomistic resolution, gaining insights into the biophysical aspects of early aggregation events of amyloids.

SIGNIFICANCE Amyloidogenic proteins have the propensity to form dense, fibrillar structures. Amyloid fibrils serve a biological function, typically used as a hormonal storage reservoir. However, amyloid proteins are prone to misfolding, aggregating into cytotoxic species that are implicated in several disease states. Understanding the biophysical characteristics of cytotoxic amyloid aggregation is important for understanding amyloid-related pathologies. This work computationally investigates the aggregation of amyloid-β_{42} (Aβ_{42})—the cytotoxic species in Alzheimer's disease—and β-endorphin$_{31}$ (βE$_{31}$), a noncytotoxic and functional amyloid species. Comparing the aggregation of cytotoxic and functional amyloids highlights the importance of hydrophobic sequences in stabilizing Aβ_{42} aggregates, whereas the charge density distribution of βE$_{31}$ reduces aggregation potential. This work provides a basis for understanding the biophysical aspects driving the amyloid cytotoxicity-functionality continuum.

INTRODUCTION

Amyloids, defined as intrinsically disordered proteins that polymerize into a cross-β structures (1–3), exhibit a unique propensity for modulating secondary and tertiary structures in response to environmental stimuli (4). Amyloid peptides simultaneously exhibit conformational flexibility and the ability to form organized fibrils, conferring them a wide range of functions to amyloidogenic species. Amyloids serve functions such as hormonal signaling and storage (5,6), biofilm formation (7), and antimicrobial activity (8,9) and are hypothesized to have been biomolecular scaffolds in prebiotic environments (4,10). The unique conformational flexibility exhibited by amyloids, however, can give rise to misfolded, cytotoxic oligomeric structures implicated in multiple diseases (11–13). Despite multifaceted functionality, low-molecular-weight oligomers formed during amyloid aggregation events can be highly cytotoxic

Submitted March 14, 2025, and accepted for publication July 31, 2025.
*Correspondence: ambrown7@vt.edu
Editor: Gregory Bowman.
https://doi.org/10.1016/j.bpj.2025.07.042
Published by Elsevier Inc. on behalf of Biophysical Society.
This is an open access article under the CC BY license (http://creativecommons.org/licenses/by/4.0/).

(13,14). Alzheimer's disease (AD) and type 2 diabetes mellitus (T2D)—both amyloid-related diseases—are the seventh and eighth leading causes of death globally (15). As such, it is critical to understand oligomeric amyloid structure at a fundamental level related to biological function and to elucidate the mechanisms of disease.

Among the functional amyloidogenic hormones are β-endorphins (βEs), a class of μ-opioid receptor agonists (16,17) produced in pituitary glands (18), the hypothalamus (19), and skin tissues (20,21). βEs are a component of the endogenous opioid system and function as analgesics (22) and as stress-response modulators (23). βEs are cleavage products of pro-opoimelanocortin and exist in several isoforms, ranging from 27 to 31 residues in length (17,24). The 31-residue isoform (βE_{31}) is the most biologically active isoform (16), exhibiting 18–33 times the potency of morphine (25). Like several protein hormones, βEs are stored in highly concentrated, membrane-bound secretory vesicles as amyloid fibrils (5,26,27). Signaling events, such as changes to pH or salt concentrations, trigger fibril disassembly mediated by a buried glutamate residue and granule exocytosis (6,28–30). Although βEs are amyloidogenic, their fibrillar or oligomeric species are not implicated in the development of amyloid-related disease states (31). In contrast, amyloid-β (Aβ) is an amyloidogenic peptide implicated in the progression of AD (32). Aβ is a 37- to 49-residue cleavage product of the transmembrane amyloid precursor protein (APP) (33); the 42-residue isomer ($A\beta_{42}$) and, to a lesser extent, the 40-residue isomer are the predominant species deposited in amyloid plaques (34). Aβ peptides are highly prone to misfolding into oligomeric structures that kill neuronal cells and induce neurological dysfunction (34). Despite the shared propensity of Aβ and βEs to adopt fibrillar morphologies, these peptides vary greatly in their cytotoxic potential, warranting investigation into sequence properties that drive amyloidogenic misfolding.

Amyloid aggregation mechanisms typically follow a sigmoidal growth curve with distinct phases: 1) a nucleation phase (or "lag phase"), 2) an elongation and growth phase, and 3) a saturated, inert fibril phase (35–37). In the nucleation phase, transient oligomer assemblies form with a cross-β structure to which additional monomeric units can be added, acting as seeds for the elongation phase (37–40). However, the oligomeric assemblies formed in the lag phase can be highly heterogeneous; an impressive range of cytotoxic $A\beta_{40}$/$A\beta_{42}$ oligomeric species have been characterized, including globular/spherical (41–43), protofibrillar (39,44–46), β-barrel (47), and flat-β sheet/β sandwich (48) morphologies. A similarly wide range of oligomeric intermediates have been identified for other amyloids implicated in other diseases, such as α-synuclein (49–52) and human islet amyloid polypeptide (hIAPP) (53,54), which are implicated in Parkinson's disease and T2D, respectively. Functional amyloids also adopt a variety of structural states and leverage their conformational flexibility to modulate signaling. For instance, the yeast prion Sup35 leverages conformational diversity to control phenotypic expression and fibril seeding conformations (55,56). There is evidence for the tight control of expression and folding for some functional amyloids, minimizing misfolding potential. This has been demonstrated for Pmel17, a mammalian protein critical to melanosome pigmentation (57,58). However, there are functional amyloids that are also involved in disease states, such as hIAPP, which is involved in glucose homeostasis but contributes to amyloid-related β cell loss in T2D, worsening disease progression (59). The lack of disease-state-related conformations sampled by amyloids such as βEs raises questions as to whether sequence-specific properties of disease-related amyloids, like Aβ, can be identified and thus targeted in therapeutic interventions. Given that Aβ and βEs share mechanistically linked aggregation-prone regions (APRs), it is critical to understand how different APRs are more likely to misfold.

Computational methods, such as molecular dynamics (MD) simulations, are useful as a complement to experimental techniques in describing the conformational landscape of early oligomeric amyloids at atomistic resolution (60–62). Extensive work has been done on the simulation of amyloidogenic fragments, such as residues 16–22 of Aβ (KLVFFAE), the amyloidogenic core (63–67) of Aβ. Simulations of the hexameric 16–22 sequence predict the formation of β-barrels (64), much like the cytotoxic cylindrin (68) and the crystallized macrocyclic 16–22 barrels with familial AD mutations (69). Simulations of full-length Aβ peptides and oligomers have recently become more computationally accessible (70,71) and have described a divergence of oligomeric alloforms between compact and extended conformations, the latter of which favor further assembly (71). These alloforms also diverge in their hydrophobic solvent-accessible surface area (SASA), which is postulated in experimental work to give rise to differing cytotoxic potential (72). Given that both cytotoxic and functional amyloids possess hydrophobic APRs (73–75), it is currently unclear how oligomerization potential or oligomeric conformations differ among amyloid species across the spectrum of functionality (Fig. 1). To this end, we utilize MD simulations to observe the oligomerization of hexameric $A\beta_{42}$ and βE_{31} to explore sequence properties that drive aggregation events and oligomeric architecture. This work seeks to understand morphological differences in cytotoxic and functional oligomer structures to aid our understanding of the genesis of cytotoxic amyloid conformations.

MATERIALS AND METHODS

MD Simulations

All MD simulations were constructed with and run using the GROMACS (76,77) software package, v.2016.3 and v.2020.4. The GROMOS96 53a6 (78) force field was employed for all simulations. This force field was chosen due to its superior replication of NMR j-coupling constants in

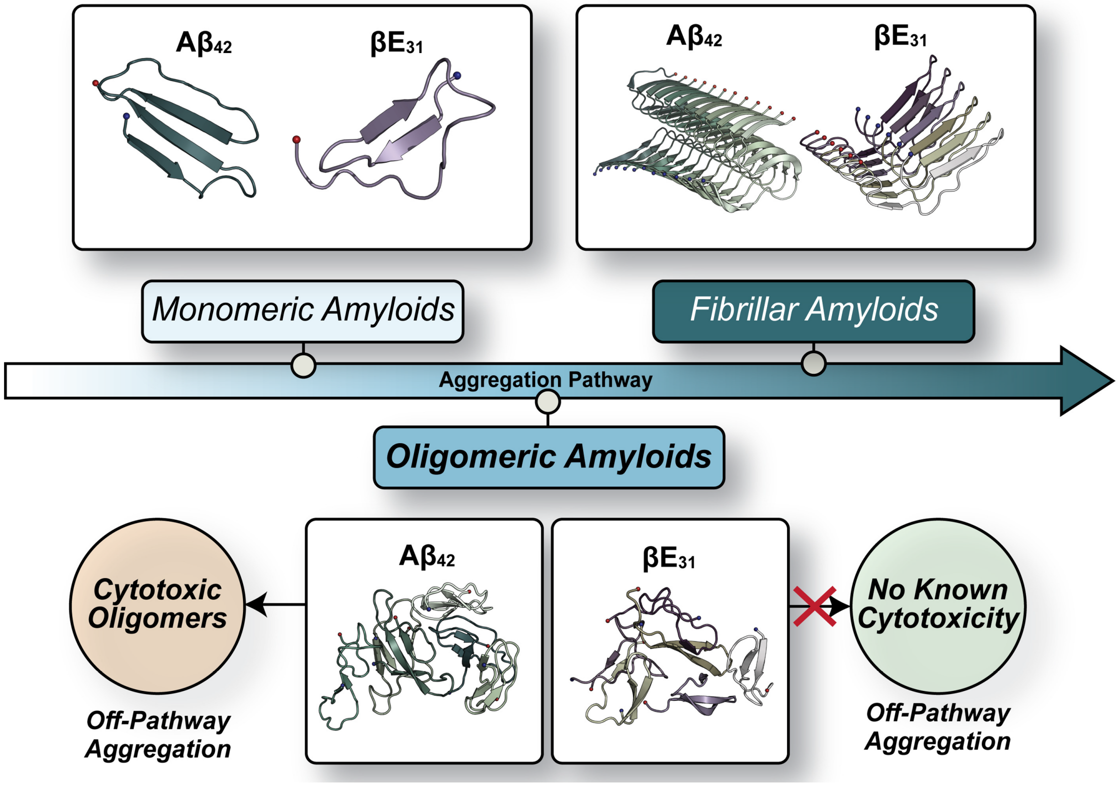

FIGURE 1 Overview of Aβ_{42} and βE_{31} aggregation pathway and propensities for forming off-pathway cytotoxic oligomers.

monomeric Aβ simulations (79) and of ordered β-barrel structures formed by [16]KLVFFAE[22] segments (64). All simulations in this work were employed as follows. Energy minimizations were performed using the steepest descent method. After minimization, a two-step equilibration was performed with position restrains on protein heavy atoms. In the first equilibration step, a 100 ps NVT ensemble was employed using the Berendsen weak coupling method (80,81) at 310 K. In the first equilibration, random velocities were assigned to generate replicate simulation. Subsequently, an isothermal-isobaric NPT ensemble was employed, using the Nosé-Hoover (82) thermostat and the Parrinello-Rahman (83) barostat at 310 K and 1 bar, respectively. After equilibration, production MD runs were performed with all restraints released. Both equilibration and production simulations were performed using a 2 fs integration step. Production simulations utilized the P-LINCS (84) algorithm to constrain bond length. The smooth particle mesh Ewald method (85,86), with cubic interpolation and 0.16 nm Fourier grid spacing, was employed to calculate long-range electrostatic interactions, using a 1.4 nm long-range interaction cutoff. Periodic boundary conditions were employed in all three spatial dimensions.

System construction

To obtain a monomeric starting structure for insertion into oligomer systems, an initial monomer was simulated for 1 μs to obtain an equilibrated structure in solution. For Aβ42, PDB: 1IYT (87) was utilized, and for βE_{31}, a single peptide was isolated from the fibril structure in PDB: 6TUB (26). For Aβ_{42}^{MUT} ($I_{31}K/I_{32}N$, $V_{36}K/G_{37}N$), mutations were made to the Aβ_{42} starting structure using PyMOL (88). Starting structures for oligomer simulations were selected by performing root mean-square deviation (RMSD) clustering over the 1 μs trajectories using a 0.3 nm cutoff. To construct the oligomer systems, peptides were placed into a cubic box (12.23 $\times$ 12.23 $\times$ 12.23 nm for Aβ_{42} and 12.21 $\times$ 12.21 $\times$ 12.21 nm for Aβ_{42}^{MUT} and βE_{31}) at least 1.4 nm apart to be outside of the long-range interaction cutoff. A minimum 1.0 nm solute-box distance was enforced. Each system was solvated using 150 mM NaCl with additional neutralizing counterions. Monomer simulations were performed in triplicate in the same manner and with the same parameters as described previously. Simulation inputs and starting structures can be found on our lab OSF (89). Monomer simulations for Aβ_{42}, βE_{31}, and Aβ_{42}^{MUT} were performed in three replicates, with each replicate simulated for 2 μs. Hexamer simulations for Aβ_{42}, βE_{31}, and Aβ_{42}^{MUT} were performed in three replicates, with each replicate simulated for 2 μs. A total of 38 μs of simulation time was performed. Starting structures for the monomeric Aβ_{42}, monomeric βE_{31}, hexameric Aβ_{42}, and hexameric βE_{31} are shown in Fig. S21. Simulation inputs and starting structures can be found on our lab OSF (90).

Analysis

Molecular visualization was performed using PyMOL (91). Data collection was performed using both the GROMACS analysis suite and MDtraj (92). Secondary structure was performed using the DSSP algorithm (93). RMSD clustering was performed on protein backbone atoms using the GROMOS method as described by Daura et al. (94), with a 0.3 nm cutoff. Statistical analysis was performed using SciPy (95). For datasets that satisfied assumptions of equal variance and normality, parametric statistical tests were employed. For normal, multilevel datasets (>2), one-way ANOVA was performed in conjunction with Tukey's HSD for post hoc comparisons, and for two-group comparisons, *t*-tests were used. For datasets that violated assumptions of normality, Kruskal-Wallis in conjunction with Dunn's

multiple comparison test were employed. Statistical difference was defined as $p < 0.05$.

Interaction frequencies were calculated to represent an aggregate of all peptide pairs. When considering intermolecular interactions, interactions for a given residue pair in a hexameric system could be calculated for 15 peptide pairs; similarly, when considering intramolecular interactions, a residue pair interaction could be calculated 6 times in a hexameric system. To simplify the analysis of residue-residue interactions in a multimeric system, we employ a dimensionality reduction technique designed to weight residue-residue interactions based on their frequency (both as an overall occupancy and the frequency across all possible pairs) and the mean distance sampled. For a given residue pair (e.g., Phe4-Phe19), the computation is as follows:

$$Residue - Residue\ Interaction\ Frequency = \frac{N\ pairs_{d \leq 0.6}}{\mu_{d \leq 0.6}} \times \frac{N\ frames\ \mu_{d \leq 0.6}}{total\ frames},$$

where $N\ pairs_{d \leq 0.6}$ equals the number of peptide pairs where the residue pair of interest is within the 0.6 nm interaction cutoff; $\mu_{d \leq 0.6}$ equals the mean of all distances within the interaction cutoff, defined as 0.6 nm; and $N\ frames\ \mu_{d \leq 0.6}$ equals the number of frames for which the residue pair of interest exhibits at least one interaction.

Unless otherwise stated, all analysis and averages presented from hexamer simulations are taken over the entire simulation period. For calculations involving eccentricity or radius of gyration, distributions and averages were taken over the 0.3–2 μs simulation period. This time frame was chosen based on the convergence of the radius of gyration, indicating the formation of a stably compact oligomer structure (Figs. S22 and S23). Boltzmann-weighted 2D histograms and inter/intramolecular interaction heatmaps were computed using concatenated data from all three replicates.

RESULTS AND DISCUSSION

Amyloidogenic peptides are expressed in an abundance of species, leveraging their conformational diversity to modify their functional state (96–98). The divergence in cytotoxic potential of amyloids may be related to oligomer architecture and dynamics, as oligomeric intermediates are thought to be the primary cytotoxic amyloid species (12,13). However, the influence of sequence on oligomerization dynamics in terms of functional differences is poorly understood. Here, we utilize MD simulations to explore the biophysical properties of monomeric and hexameric Aβ_{42} and βE$_{31}$—the former being central to AD pathology. At the time of writing, there are no published computational investigations that use a comparison to functional amyloid dynamics as a methodology to better understand the behavior of cytotoxic oligomer species. Addressing this fundamental knowledge gap is crucial to unraveling the specific biophysical mechanisms that differentiate functional amyloid behavior from cytotoxic processes, as related to amyloid structure-function.

Monomeric Aβ_{42} and βE$_{31}$ exhibit divergence in structural disorder

In their monomeric state, Aβ_{42} and βE$_{31}$ are both considered to be intrinsically disordered (99–101). However, in its oligomeric form, Aβ_{42} can adopt a wide variety of conformations, including highly organized motifs such as proto-fibrils, β-barrels, and β sandwiches (39,44–48). These architectures all involve ordered β sheet formation, suggesting that monomeric Aβ_{42} can adopt stable, folded states. In the context of comparing cytotoxic and functional amyloid peptides, it could be possible that a more conformationally flexible peptide would be less prone to folding stably, therefore reducing the probability of forming ordered oligomer structures with cytotoxic potential.

To this end, 2 μs simulations of monomeric Aβ_{42} and βE$_{31}$ were performed in triplicate to assess their structure and dynamics. Secondary structure calculations indicate that both peptides have a high propensity for adopting β strand content (39.7% and 33.2% β strand content for Aβ_{42} and βE$_{31}$, respectively) (Fig. S1 *A*; Table 1). In all replicates, the Aβ_{42} monomer adopts a β sheet involving hydrophobic-core residues ^{18}VFF^{20} and hydrophobic C-terminal residues ^{38}GVV^{40}, engaged in β strand content for >90% of all simulation time in aggregate (6 μs) (Fig. 2 *A*). These residues form a core β sheet in which other segments can join, typically adopting a sheet comprising 3–4 β strands, sampling sheets formed with both N- and C-terminal strands. Principal-component analysis (PCA) of secondary structure content reveals three well-resolved clusters that correspond to each replicate, indicating that any given folded structure is highly stable, changing minimally over the simulation period (Fig. S2 *A*). In contrast, βE$_{31}$ exhibits a flatter secondary structure distribution, indicating higher variability in sampled β strand content (Fig. S2 *B*). Residues ^{3}GFMTS^{7} sample β strand content at the highest frequency (~75% of all simulation time, 6 μs), where adjacent strands forming a sheet or β-hairpin with the N-terminus are highly variable (Fig. 2 *B*); residue interaction heatmaps indicate that N-terminal residues ^{3}GFMTS^{7} interact uniformly with the rest of the peptide (Fig. S3). Furthermore, PCA of sampled secondary structure content does not reveal well-defined, clustered replicates, indicating increased structural variability (Fig. S2 *B*).

Although βE$_{31}$ and Aβ_{42} both exhibit signatures of disorder, these data imply that βEs may exhibit more inherent disorder than Aβ. The Aβ_{42} sequence is distinctly partitioned into four segments, alternating in charge density and hydrophobicity, whereas the βE$_{31}$ sequence instead consists of short hydrophobic patches interspersed by polar or

TABLE 1 Secondary structure percentages for Aβ_{42} and βE$_{31}$ monomer simulations

	Aβ_{42} monomer secondary structure (%)			βE$_{31}$ monomer secondary structure (%)		
Replicate	Coil	β sheet	Helix	Coil	β sheet	Helix
Replicate 1	59.1	40.9	0.02	65.4	34.5	0.07
Replicate 2	55.8	44.1	0.02	61.4	38.5	0.06
Replicate 3	65.8	34.2	0.07	73.4	26.5	0.16
All	60.2	39.7	0.04	66.7	33.2	0.09

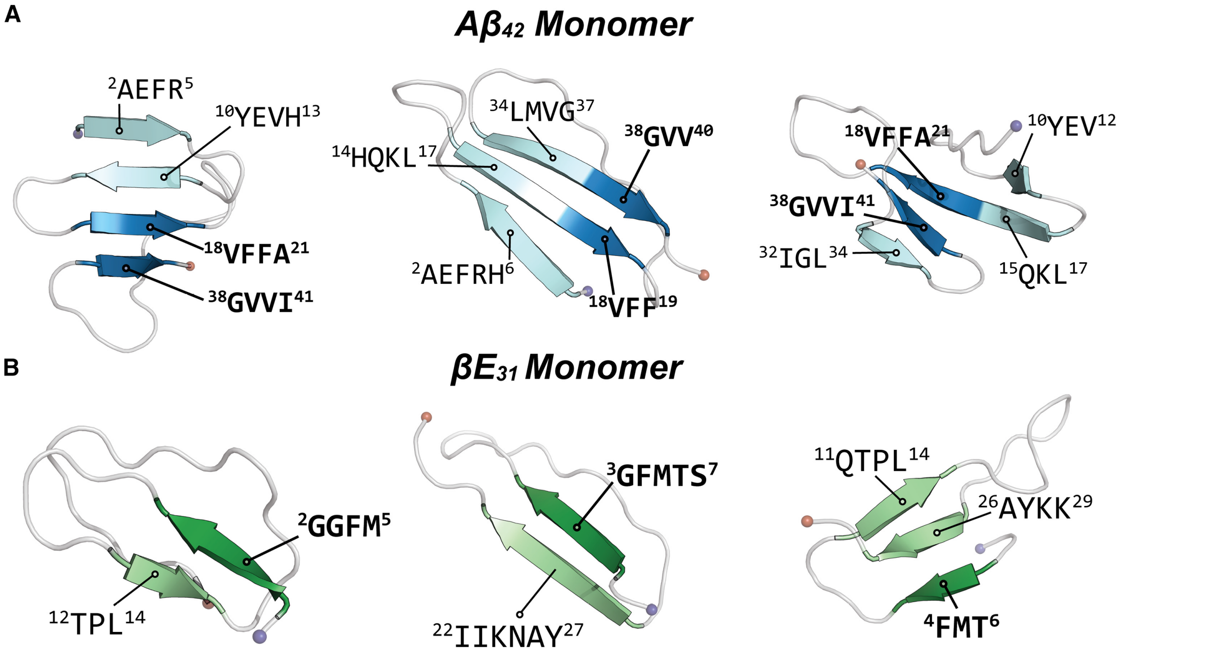

FIGURE 2 β sheet motifs sampled in monomeric amyloid simulations. Snapshots from simulation visualizing predominant β strand motifs across replicates for (*A*) Aβ_{42} and (*B*) βE$_{31}$ monomers. Most frequent β strand residues are colored dark blue or dark green for Aβ_{42} and βE$_{31}$, respectively. Variable strands are colored light blue or light green for Aβ_{42} and βE$_{31}$, respectively.

charged segments (Fig. 3, *A* and *B*). The hydrophobic segmentation present in the Aβ_{42} sequence likely favors collapsed conformations to minimize hydrophobic solvent exposure. To probe the role of hydrophobic segmentation on monomeric folding, a monomeric Aβ_{42} mutant ($I_{31}K/I_{32}N$, $V_{36}K/G_{37}N$) (Aβ_{42}^{MUT}) was simulated in triplicate for 2 μs. The hydrophobic C-terminus was disrupted with KN residue motifs, which appear twice in the βE$_{31}$ sequence, interspersed between short hydrophobic patches (Fig. 3 *C*). The Aβ_{42}^{MUT} monomer, much like βE$_{31}$, exhibits reduced secondary structure continuity relative to Aβ_{42} (Fig. S1 *C*), where PCA of secondary structure is not well resolved by the replicate (Fig. S2 *C*). Aβ_{42}^{MUT} monomers exhibited a 35% reduction in hydrophobic core residues (^{17}LVFFA^{21} and ^{37}GGVVI^{41}) relative to wild-type (WT) Aβ_{42} (Figs. 3 *D* and S4). This indicates that the inclusion of bulky, polar residues prevents the hydrophobic collapse of the core and C-terminal residues. Furthermore, the Aβ_{42}^{MUT} C-terminal amphipathic segment (^{22}EDVGSNK^{28}) sampled virtually no β strand content in WT Aβ_{42}, where it primarily functioned as a disordered linker between the hydrophobic core and C-terminal β sheet. In Aβ_{42}^{MUT}, this segment sampled both β strand and helical content (Fig. S1 *C*). The loss of hydrophobic collapse, characteristic of WT Aβ_{42}, supports the notion that hydrophobic segmentation favors continuous, β sheet-containing monomeric structures. As such, we postulate that the distinct hydrophobic segmentation of the Aβ_{42} is critical to stabilizing monomeric folding, thus stabilizing ordered oligomeric species and contributing to nucleation events.

βE$_{31}$ hexamers are more polymorphic than Aβ_{42}

We simulated the formation of hexameric Aβ_{42} and βE$_{31}$ to examine both multiple potential oligomer architectures and the interactions present in each structural state sampled. To examine overall oligomer architectures, RMSD clustering was performed, and dominant morphologies were visualized (Fig. S5). Visually, the Aβ_{42} hexamer structures sampled can be described as a continuum between 1) a globular, spherical, and relatively disordered hexamer and 2) an elongated, β strand-rich hexamer. 2D probability distributions of sampled eccentricity and β strand content indicate two regions of high probability, consistent with the dichotomy of observed conformational states corresponding to a relatively spherical/disordered state (globular) and a rod-shaped/folded state (elongated) (Fig. 4 *A*; Table 2). Replicates 1 and 2 primarily sampled the elongated and globular states, respectively, whereas replicate 3 transitioned from elongated to globular over the simulation period (Figs. 4 *A* and S5). Elongated and globular conformations sampled in this work are compatible with experimental characterizations; experimental characterizations of Aβ_{42} oligomer morphologies have reported several conformational states, including globular (41–43), proto-fibrillar (39,44–46), β sandwich (48), and β-barrel species (47). Sampling of highly organized, experimentally reported conformations, such as β-barrels, was absent; achieving sampling of such organized conformational states would likely require enhanced sampling techniques. In contrast, βE$_{31}$ hexamers were highly diverse, exhibiting little heterogeneity across replicates in terms of β strand content

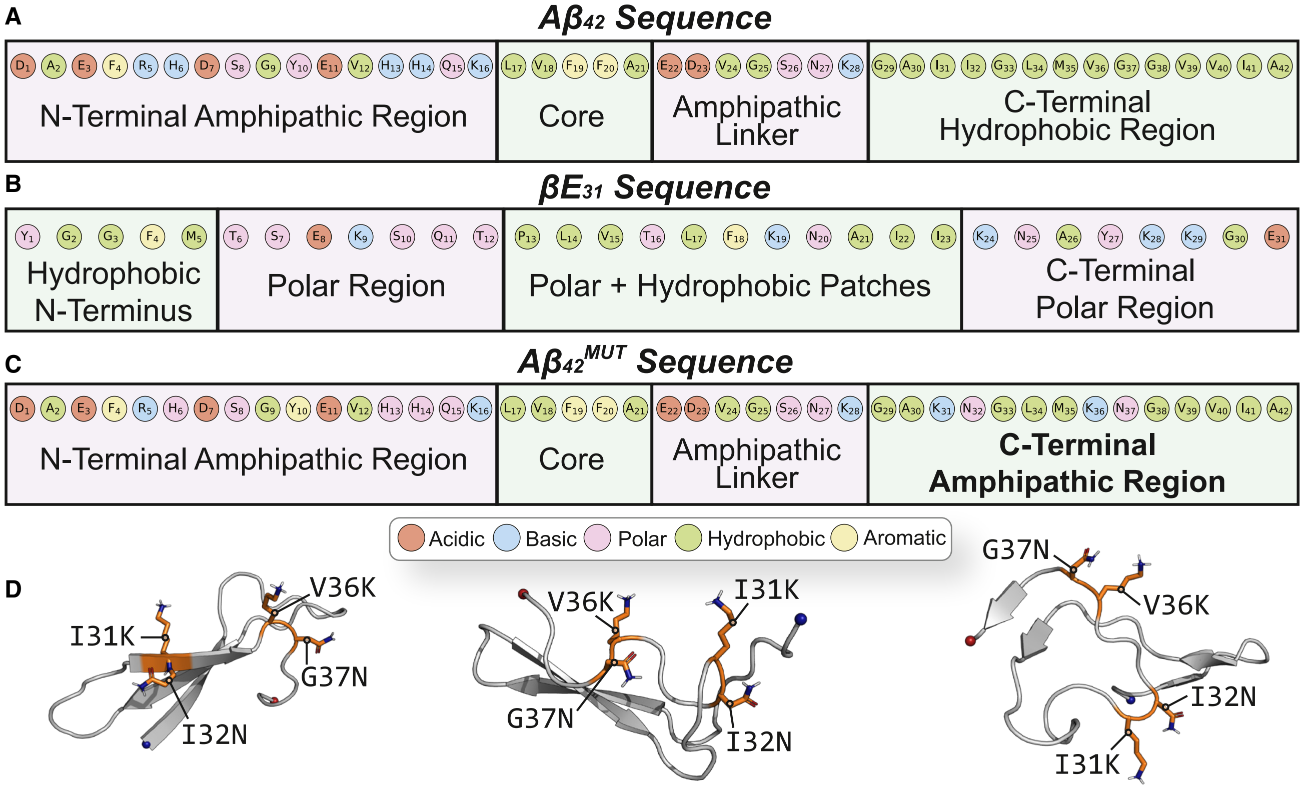

FIGURE 3 Sequences of Aβ_{42}, βE$_{31}$, and Aβ_{42}^{MUT}. (*A–C*) Sequences of (*A*) Aβ_{42}, (*B*) βE$_{31}$, and (*C*) Aβ_{42}^{MUT}, colored by residue type (acidic, *red*; basic, *blue*; polar, *pink*; hydrophobic, *green*; aromatic, *yellow*). (*D*) Snapshots of Aβ_{42}^{MUT} monomer simulations visualizing mutation disruption to C-terminal secondary structure. Mutated residues are colored as orange sticks.

and eccentricity (Figs. 4 *B* and S6). The 2D probability distribution of eccentricity and β strand content indicate three regions of high probability, each corresponding to a given replicate that sampled a distinct structure: 1) a roughly circular arrangement of β strands, 2) a β strand dimer and a tetramer of mixed secondary structure character, and 3) a globular, largely disordered hexamer with transient α-helical content (Fig. 4 *B*; Table 2). The relative diversity of βE$_{31}$ hexamer structures suggests a rougher, more diverse free energy landscape in terms of oligomer architectures. It should be noted that in this study, we have performed MD with a conventional NPT ensemble. As such, the architectures sampled in this work are by no means exhaustive. Advanced sampling techniques, like replica exchange (102,103) and Gaussian accelerated MD (104,105) are more robust methods for sampling a wide conformational space.

Examination of intramolecular interactions reveal mechanistic insights into the relative homogeneity of Aβ_{42} hexamers with respect to βE$_{31}$. Intramolecular interaction probabilities between hydrophobic core residues (^{16}KLVFFAE^{21}) and hydrophobic C-terminal residues for Aβ_{42} peptides are sampled consistently and uniformly across replicates, reflecting the preservation of stable β strand motifs sampled in monomeric simulations (Figs. 5 *A* and S7). The peptide subunits of the hexamer exhibit a strong preference for adopting β sheet motifs, including a C-terminal β-turn-β motif comprising residues ^{32}IIGLM^{35} and ^{38}GVVI^{41} and a β sheet comprising ^{17}LVFFA^{21}, ^{38}GVVI^{41}, and ^{32}IIGLM^{35} (Figs. 5 *B*, S8, and S9), consistent with NMR observations (106,107). Previous studies have found that the Aβ_{42} intramolecular β-hairpin structures are involved in the formation of intermolecular β sheets (108–110). Counts of intermolecular β strand content indicate that across replicates, hydrophobic C-terminal residues—particularly ^{32}IIGLM^{35} and ^{38}GVVI^{41}—consistently engage in intermolecular β sheets in all replicates, consistent with previous observations (Fig. S10). Intramolecular interactions in the amphipathic Aβ_{42} N-terminus exhibit more variability across replicates. Indeed, for Aβ_{42}, interactions between residues with polar or charged side chains are associated with higher variance in interaction propensity across replicates and peptides ($p \leq 0.5$) (Fig. S11).

Intramolecular interactions sampled by βE$_{31}$ exhibit high variance across peptides and replicates, indicating a higher degree of disorder relative to Aβ_{42} (Fig. 5 *A*). The βE$_{31}$ sequence is charge dense relative to Aβ_{42}, whose well-defined hydrophobic regions likely favor a collapsed state, as observed in monomeric simulations. R$_g$ calculations on peptide subunits indicate that oligomerized Aβ_{42} peptides are more compact relative to βE$_{31}$ peptides (Aβ_{42} mean = 1.09 ± 0.01 nm, βE$_{31}$ mean = 1.24 ± 0.03 nm) (Fig. S12). Aβ_{42} β-hairpin motifs in tandem

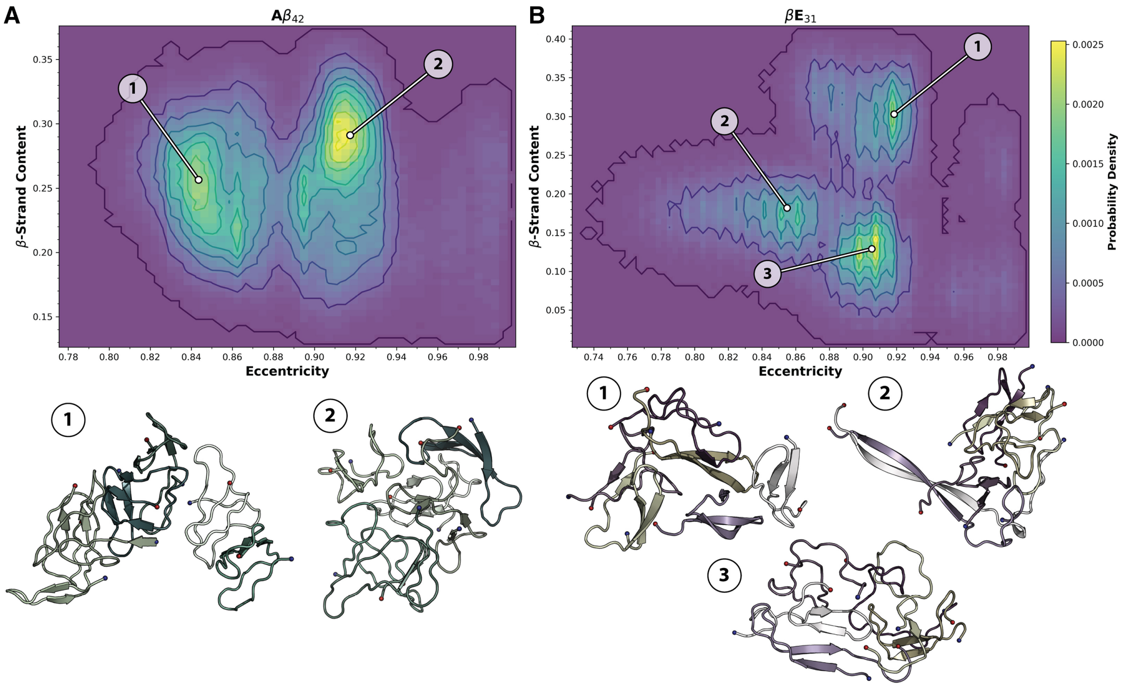

FIGURE 4 Boltzmann-weighted 2D histograms of eccentricity and β strand content. Boltzmann-weighted 2D histograms of eccentricity and β strand content for (*A*) Aβ_{42} and (*B*) βE$_{31}$ hexamers, taken over the entire 2 μs simulation period across all replicates. Structures below plots correspond to the labeled points on the histograms in terms of β strand content and eccentricity. (*A*) Aβ_{42} hexamers are shown as blue/green cartoons, and (*B*) βE$_{31}$ hexamers are shown as yellow/purple cartoons.

with an unstructured N-terminus have been resolved via native ion mobility-mass spectrometry, consistent with the observed conformations (47). This supports the concept that Aβ_{42} hydrophobic segmentation leading to collapsed β strand motifs contributes to the stabilization of oligomer conformations.

The intermolecular interactions present in the simulated hexamers were examined by calculating interaction probabilities (Fig. S13). Despite sampling two distinct architectures in terms of β strand content and eccentricity, each of the three Aβ_{42} replicates displayed a unique interaction profile. Replicate 1 exhibited well-distributed contacts across all regions of the peptide. The highest-frequency interactions in replicate 1 were present between 1) hydrophobic core residues (^{17}LVFFA^{21}) and the C-terminus (^{29}GAIIGL^{34}) and 2) N-terminal residues on adjacent chains (^{5}RHDSGY^{10}). In replicate 2, prevalent interactions included both C-terminal/C-terminal interactions (^{29}GAI^{31} with ^{34}LMV^{36}) and interactions between the amphipathic linker region (^{22}EDVG^{25}) and the polar N-terminus (^{13}HHQK^{16}). In replicate 3, interactions between the 16–23 segment (^{16}KLVFFAED^{23}) and the N-terminus (^{1}DAEFRGDS^{8}) were the most prominent. In terms of π-stacking, Tyr10 exhibited a relatively high propensity for interactions with other aromatic residues, although its interactions were nonspecific (Phe4, His13, Phe19, and Phe20) (Fig. S14). Stacking between Phe4/Phe20, Phe4/Tyr10, and Phe4/Phe20 was commonly sampled, suggesting a role of Phe4 and Tyr10 in coordinating the interaction between the amphipathic N-terminus and the hydrophobic core. Interestingly, mutating Tyr10 to a synthetic residue (para-amino-phenylalanine) impairs the aggregation propensity of Aβ_{42}, suggesting that the hydroxyl group is important for aggregating stability in addition to aromaticity (111). However, analysis of intermolecular hydrogen bonds over the simulation indicates that hydrogen bonds involving Tyr10 were not sampled (Fig. S15). Salt bridge formation was also assessed (Table S2). The residue with the highest probability to engage in a salt bridge (~33%–75% of the aggregated simulation period) was the N-terminal Asp1, which was involved in almost all high-occupancy salt bridges. These salt bridges formed via both the amino and carboxylic groups. The most probable salt bridge partners for Asp1 include Asp1, Asp7, Lys16, and Lys28.

TABLE 2 Centroids of Boltzmann-weighted 2D histograms of eccentricity and β strand content for Aβ_{42} and βE$_{31}$ hexamers

	Aβ_{42} hexamer		βE$_{31}$ hexamer	
Centroid	Eccentricity	β strand (proportion)	Eccentricity	β strand (proportion)
1	0.84	0.25	0.90	0.31
2	0.91	0.29	0.83	0.23
3	–	–	0.91	0.19

Centroids were determined by K-means clustering of eccentricity and β strand content, taken over the entire 2 μs simulation period across all replicates.

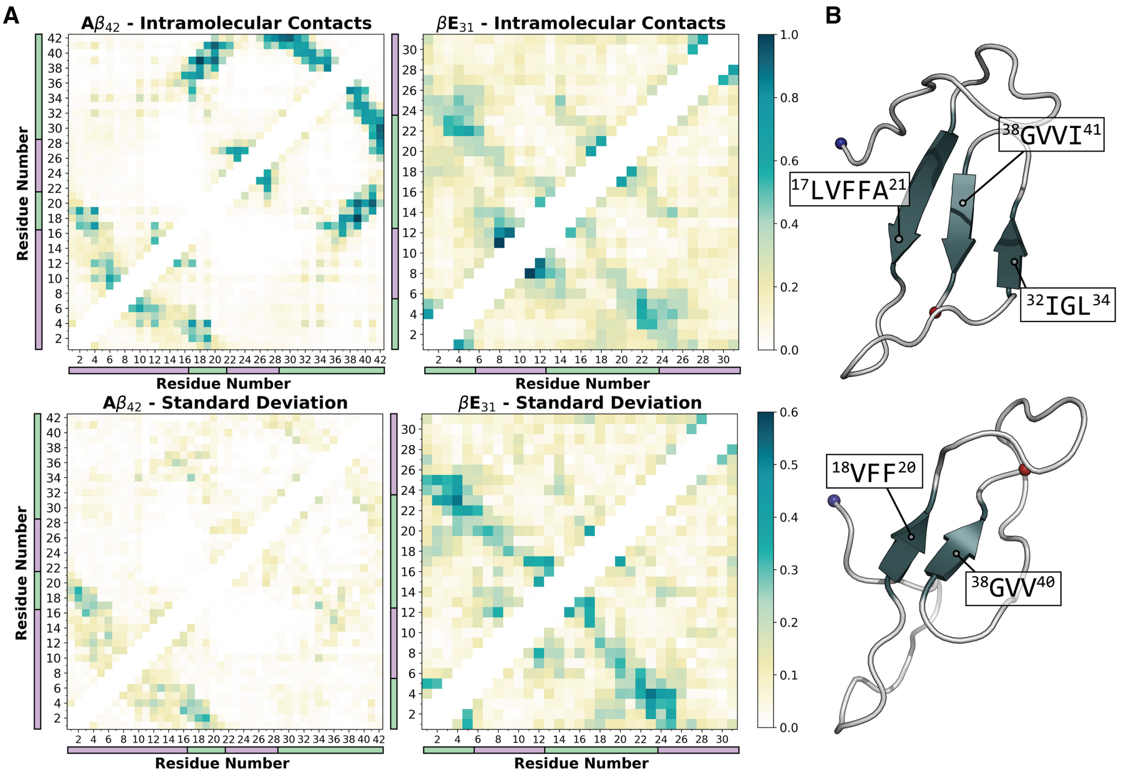

FIGURE 5 Intramolecular interaction probability heatmaps. (*A*) Intramolecular interaction probability heatmaps for (*top*) Aβ_{42} and (*bottom*) βE$_{31}$ hexamer simulations. Purple and green bars correspond to residue regions as labeled in Fig. 3, *A* and *B*, for Aβ_{42} and βE$_{31}$, respectively. Heatmaps represent a composite probability of all three replicates over the 2 μs simulation period. (*B*) Visualizations of predominant Aβ_{42} secondary structure motifs. Coils are shown as gray cartoon, and β strands are shown as dark blue cartoon. The N- and C-termini are shown as blue and red spheres, respectively.

βE$_{31}$ hexamers, like Aβ_{42}, each display a unique intermolecular interaction profile (Fig. S13). Replicate 1 exhibits interactions between the N-terminal polar region (^{6}TSEKSQT^{12}) and the C-terminal polar region (^{26}KNAYKKGE^{31}). This interaction is likely mediated by opposing charges present in either region. A salt bridge between Lys9 and Glu31 is sampled by two separate peptide chains, present for 12.7%–16.5% of the aggregated period (Table S3). This replicate also exhibited interactions between the N-terminus (^{1}YGGFM^{5}) and the 16–20 segment (^{17}TLFKN^{20}), mediated primarily by the stacking of Phe4 and Phe18 (Fig. S14). For replicate 2, interaction probabilities reflect the formation of a dimeric, parallel β strand along most of the peptide (Fig. 4 *B*). The four remaining peptides display interactions primarily between N-terminal segments, ^{1}YGGFM^{5}, and ^{8}EKSQTP^{13}. Replicate 3 displayed interactions between adjacent ^{19}KNAIIK^{24} regions, between adjacent ^{12}TPLVT^{16} regions, and between 19KNAIIK^{24} and ^{12}TPLVT^{16} regions. The propensity for π-stacking in replicate 1 and the high probability of interactions between small hydrophobic patches suggest that hydrophobic packing is also critical to the association of βE$_{31}$ peptides. The hydrophobic packing present in both Aβ_{42} and βE$_{31}$ oligomer formation is consistent with the burial of hydrophobic APRs.

To further assess the role of hydrophobic packing on hexamer formation, we assessed the SASA of each residue, normalized by residue size, over the oligomerized simulation period (0.3–2 μs) (Fig. S16 *A*). Aβ_{42} oligomers form such that solvent exposure of hydrophobic residues is minimized; among all replicates, Phe4, Leu17, Phe19, Val39, and Ile41 are the least solvent-exposed residues in all replicates (range: 0.015 $\pm$ 0.012–0.17 $\pm$ 0.05 nm^2 n$_{\text{atoms}}^{-1}$), indicating that the packing of these residues is involved in hexamer formation. Like Aβ_{42}, βE$_{31}$ also exhibited hydrophobic packing, where the SASAs of Gly3, Phe4, Thr6, Thr12, Val15, Ala21, and Gly30 were minimized (Fig. S16 *B*). The least solvent-exposed residue in all βE$_{31}$ hexamer simulations—Phe4 in replicate 2—exhibited a normalized SASA of 0.13 $\pm$ 0.05 nm^2 n$_{\text{atoms}}^{-1}$, indicating that βE$_{31}$ hexamers were overall more solvent accessible than Aβ_{42} hexamers, despite their smaller size. This suggests that hydrophobic packing exhibited by βE$_{31}$ hexamers is less efficient. To assess the stability of intermolecular interactions in the hexameric structures, interaction occupancies were calculated for every residue pair. Aβ_{42} appears to have a higher proportion of interaction occupancies that persist for >60% of the simulation, suggesting that intermolecular contacts formed by Aβ_{42} peptides are, to some degree, more stable (Fig. S17). Interestingly,

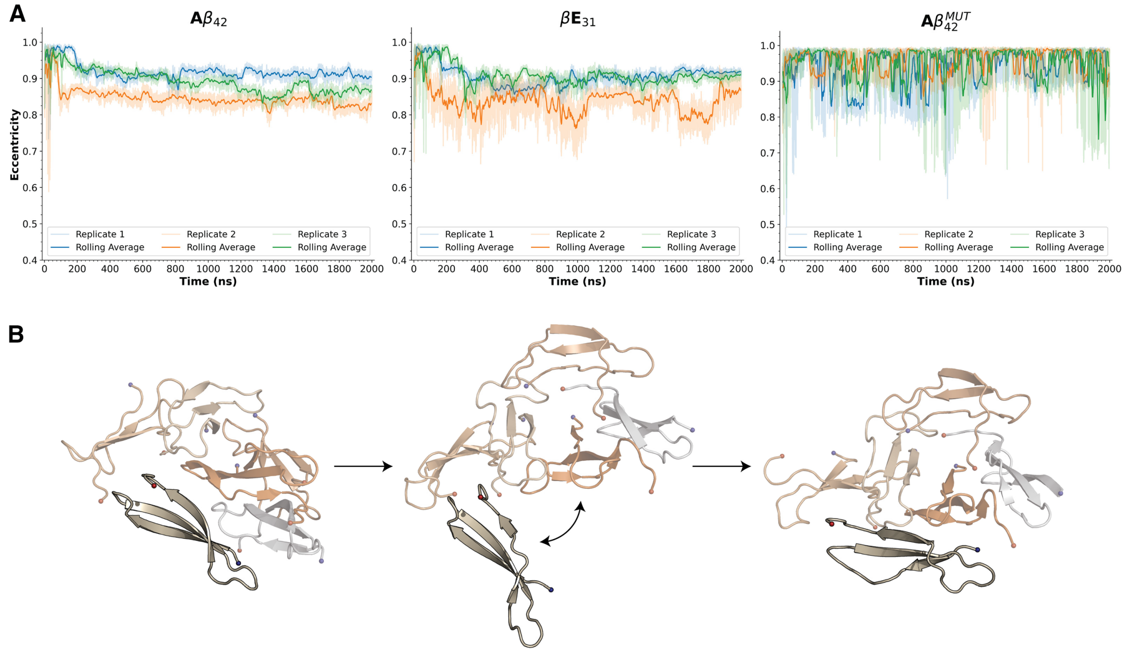

FIGURE 6 Eccentricity over time from hexamer simulations. (*A*) Eccentricity over time for Aβ_{42}, βE$_{31}$, and A$\beta_{42}{}^{MUT}$. Rolling average (window = 1000 frames) is shown as a dark line, and the raw eccentricity data are shown as a lighter fill. (*B*) Snapshots from the simulation of A$\beta_{42}{}^{MUT}$ demonstrating repeated association and disassociation of peptides from the core oligomer.

experimental studies of coaggregated Aβ_{42} and βE$_{31}$ indicate that βE$_{31}$ has a higher propensity for interacting with Aβ_{42} than with other βE$_{31}$ peptides, thus reducing Aβ_{42} aggregation and mitigating cytotoxicity (112). This suggests that the hydrophobicity of βE$_{31}$ APRs is not significant enough to drive disease-related oligomerization. Additionally, the aggregation of βE$_{31}$ is dependent on heparan sulfate (26). Together, these data support the role of hydrophobic APRs in Aβ_{42} oligomerization events (113,114) and may indicate that peptides lacking defined hydrophobic regions are less prone to aberrant aggregation events.

C-terminal mutations destabilize Aβ_{42} hexameric stability

Previous computational work has identified the hydrophobic core/C-terminal β-hairpin to be critical in the formation of Aβ oligomers (107–110,115). Furthermore, β-hairpins are the constituents of cytotoxic β-barrels formed from amyloidogenic segments (68,69,116). To further explore the impact of Aβ_{42} hydrophobic segmentation on oligomeric conformational dynamics, the formation of A$\beta_{42}{}^{MUT}$ hexamers was simulated for 2 μs in triplicate. Interestingly, A$\beta_{42}{}^{MUT}$ oligomers sampled higher β strand content overall, but ^{18}VF^{19}/^{38}GVV^{40} β strand content was significantly reduced in A$\beta_{42}{}^{MUT}$ oligomers relative to Aβ_{42} (A$\beta_{42}{}^{WT}$) oligomers (Fig. S18, *A* and *B*). Global increases to β strand content probabilities were observed for N-terminal residues 4–17 and amphipathic linker residues 21–24. In A$\beta_{42}{}^{WT}$ oligomers, residues ^{35}MVG^{37} have a high probability of serving as a linker between ^{32}IGL^{34} and ^{38}GVV^{40} β strand segments; these residues have an increased probability of participating in β sheets in A$\beta_{42}{}^{MUT}$ oligomers (Fig. S18 *C*). To probe the stability of β strand content sampled by the C-terminus in aggregate, the pairwise Hamming distance of secondary structure content in residues 28–42 was calculated over all frames and replicates for A$\beta_{42}{}^{WT}$ and A$\beta_{42}{}^{MUT}$ hexamers. On average, A$\beta_{42}{}^{MUT}$ exhibited higher variation relative to A$\beta_{42}{}^{WT}$ (4.7 $\pm$ 2.5 vs. 3.4 $\pm$ 1.9, respectively), suggesting that the amphipathic β sheets formed are less stable than those formed by hydrophobic sequences (Table S4).

WT Aβ_{42} oligomer morphologies primarily sampled two modes of eccentricity corresponding to globular and elongated morphologies, exhibiting relatively low variance across the simulation period (Figs. 6 *A* and S19). Similarly, βE$_{31}$ oligomers exhibited both high- and low-eccentricity states, albeit with slightly more variation, on average, relative to Aβ_{42}. In contrast, A$\beta_{42}{}^{MUT}$ exhibited rapid interconversions between globular and extended conformations. Upon examination of dominant morphologies from RMSD clustering, the large fluctuations in eccentricity can be attributed to repeating associations and disassociations of individual peptides or larger units (Figs. 6 *B* and S19). Hydrophobic SASA is significantly increased in A$\beta_{42}{}^{MUT}$ oligomers with respect to WT oligomers (61.42 $\pm$ 3.24 vs. 56.25 $\pm$ 2.56 nm^2, respectively), despite the overall maximum hydrophobic SASA having been reduced because of mutations (Fig. S20). This reflects a reduced propensity for hydrophobic packing, and suggests that the

intermolecular contacts formed, which largely shifted to favor polar contacts, are generally less favorable than those in Aβ_{42} and βE_{31} oligomers. Hexamers losing their structural integrity when polar mutations are inserted into the C-terminus further supports the role of C-terminal hydrophobicity in stabilizing oligomeric structures and the role of charge distribution in destabilizing amyloid oligomeric structures.

CONCLUSIONS

We have demonstrated that Aβ_{42} hexamers adopt highly stable intramolecular contacts and, although polymorphic, adopt predictable global architectures, partly influenced by the hydrophobic collapse of individual peptides. The functional amyloid βE_{31}, and the theoretical Aβ_{42}^{MUT}, in contrast, exhibit more varied conformational landscapes. Based on these findings, we propose potential connections to how these biophysical properties may influence the broader cytotoxic potential of Aβ_{42} oligomers. First, the Aβ_{42} sequence contains hydrophobic APRs. The ^{16}KLVFFAE^{22} segment of Aβ, a hydrophobic sequence flanked by charged residues, readily self-assembles into fibrils (117). Other small amyloidogenic segments, like ^{20}SNNFGAILSS^{29} of hIAPP, are capable of self-assembly, aggregating primarily via hydrophobic packing of Phe23 residues (64,118). These APRs could theoretically drive uncontrolled aggregation characteristic of disease states, whereas βE_{31} aggregation in vivo is assisted by glycosaminoglycans present in secretory granules (26).

A second important observation is that disease-related amyloids, like Aβ_{42}, can exert their cytotoxic effects via membrane perturbations (48,119–122). Two commonly proposed mechanisms of membrane perturbation include the formation of β-barrel pores (121,123), and via lipid extraction (122). The formation of β-barrel pores may be a thermodynamically spontaneous conformational state to reduce unfavorable hydrophobic interactions with solvent. Theoretical studies of computationally derived Aβ_{42} β-barrels consist of hydrophobic residues building the barrel core and N-terminal and amphipathic linker residues remaining disordered in solution (47). Predictive models (91) for transmembrane protein sequences have been developed using hydrophobicity scales (90,124) as parameters. Although hydrophobicity is not the only feature that controls transmembrane regions or membrane insertion, it is an important property that the Aβ_{42} satisfies. Finally, the hydrophobic collapse driven by the Aβ_{42} sequence is theoretically suited to act as a detergent or otherwise bind and sequester lipids in the environment. The partitioning of polar and hydrophobic regions in folded Aβ_{42} structures could accommodate simultaneous binding with hydrophobic lipid tails and zwitterionic polar heads. Experimental work has determined that free lipids present at critical concentrations alter amyloid oligomer morphologies and mechanisms of cytotoxicity (123), and lipids have been resolved as components of matured Aβ fibrils in vivo (125,126). The sequences of functional amyloids, like that of βE_{31}, may be less amenable to barrel formation or lipid binding due to more evenly dispersed charge density, lacking the hydrophobic surface area necessary for such conformations to be feasible.

In this work, we have identified the hydrophobic segmentation of Aβ_{42} as a critical component in stabilizing the oligomeric structures sampled in this work. Monomeric and hexameric Aβ_{42} adopt a β strand motif driven by hydrophobic collapse of the hydrophobic core and C-terminus (Figs. 2 *B* and 3 *B*). The charge-dense, functional βE_{31} exhibits a broader landscape of hexameric conformations relative to Aβ_{42} hexamers (Fig. 3 *A*). The mutation of Aβ_{42} introducing charged and polar residues to the hydrophobic C-terminus destabilizes hexamer formation, inhibits hydrophobic packing (Fig. 4 *B*), and creates a broader free energy landscape for oligomeric conformations (Fig. 3 *A*). As such, we postulate that hydrophobic collapse is a determining factor in the continuum of amyloid functionality and cytotoxicity. An important limitation of this study to note is the scope; only including Aβ_{42} and βE_{31} does not consider the vast, diverse amyloidogenic landscape beyond these two peptides, ranging in cytotoxicity and functionality. Furthermore, amyloidogenic peptides can be both functional and cytotoxic, depending on aggregation environments, such as the hormone-peptide hIAPP (127). A larger-scale study including more peptides across the spectrum of functionality would be more robust for gleaning understanding of how sequence effects simulated oligomer structure dynamics and architectures. However, we believe that this work provides an argument to further probe the sequence effects that govern amyloid oligomer cytotoxicity.

ACKNOWLEDGMENTS

The authors thank Advanced Research Computing at Virginia Tech for access to computing resources. This work was supported by the National Science Foundation CAREER Award (2237521) to A.M.B.: “CAREER: Resolving the Influence of Biologically Relevant Microenvironments on Amyloid Aggregation.”

AUTHOR CONTRIBUTIONS

A.M.B. and K.M.K. designed the research protocol. K.M.K. and H.Z. performed simulations. K.M.K., H.Z., and A.M.B. analyzed data. K.M.K. and A.M.B. prepared the manuscript.

DECLARATION OF INTERESTS

The authors declare no competing interests.

SUPPORTING MATERIAL

Supporting material can be found online at https://doi.org/10.1016/j.bpj.2025.07.042.

REFERENCES

1. Levine, Z. A., L. Larini, …, J.-E. Shea. 2015. Regulation and aggregation of intrinsically disordered peptides. *Proc. Natl. Acad. Sci. USA.* 112:2758–2763.
2. Gallardo, R., N. A. Ranson, and S. E. Radford. 2020. Amyloid structures: much more than just a cross-β fold. *Curr. Opin. Struct. Biol.* 60:7–16.
3. Cawood, E. E., T. K. Karamanos, …, S. E. Radford. 2021. Visualizing and trapping transient oligomers in amyloid assembly pathways. *Biophys. Chem.* 268:106505.
4. Maury, C. P. J. 2018. Amyloid and the origin of life: self-replicating catalytic amyloids as prebiotic informational and protometabolic entities. *Cell. Mol. Life Sci.* 75:1499–1507.
5. Maji, S. K., M. H. Perrin, …, R. Riek. 2009. Functional Amyloids As Natural Storage of Peptide Hormones in Pituitary Secretory Granules. *Science.* 325:328–332.
6. Nespovitaya, N., J. Gath, …, R. Riek. 2016. Dynamic Assembly and Disassembly of Functional β-Endorphin Amyloid Fibrils. *J. Am. Chem. Soc.* 138:846–856.
7. Akbey, Ü., and M. Andreasen. 2022. Functional amyloids from bacterial biofilms - structural properties and interaction partners. *Chem. Sci.* 13:6457–6477.
8. Soscia, S. J., J. E. Kirby, …, R. D. Moir. 2010. The Alzheimer's disease-associated amyloid beta-protein is an antimicrobial peptide. *PLoS One.* 5:e9505.
9. Oh, J., J. G. Kim, …, I. Hwang. 2007. Amyloidogenesis of type III-dependent harpins from plant pathogenic bacteria. *J. Biol. Chem.* 282:13601–13609.
10. Maury, C. P. J. 2015. Origin of life. Primordial genetics: Information transfer in a pre-RNA world based on self-replicating beta-sheet amyloid conformers. *J. Theor. Biol.* 382:292–297.
11. Zaman, M., A. N. Khan, …, S. M. Zakariya. 2019. Protein misfolding, aggregation and mechanism of amyloid cytotoxicity: An overview and therapeutic strategies to inhibit aggregation. *Int. J. Biol. Macromol.* 134:1022–1037.
12. Cline, E. N., M. A. Bicca, …, W. L. Klein. 2018. The Amyloid-β Oligomer Hypothesis: Beginning of the Third Decade. *J. Alzheimers Dis.* 64:S567–S610.
13. Haataja, L., T. Gurlo, …, P. C. Butler. 2008. Islet Amyloid in Type 2 Diabetes, and the Toxic Oligomer Hypothesis. *Endocr. Rev.* 29:303–316.
14. Siddiqi, M. K., S. Malik, …, R. H. Khan. 2019. Cytotoxic species in amyloid-associated diseases: Oligomers or mature fibrils. *Adv. Protein Chem. Struct. Biol.* 118:333–369.
15. Murphy, S. L., K. D. Kochanek, …, E. Arias. 2024. Mortality in the United States, 2023. NCHS Data Brief, no 521. National Center for Health Statistics. https://dx.doi.org/10.15620/cdc/170564.
16. Deakin, J. F., J. O. Doströvsky, and D. G. Smyth. 1980. Influence of N-terminal acetylation and C-terminal proteolysis on the analgesic activity of beta-endorphin. *Biochem. J.* 189:501–506.
17. Nicolas, P., and C. H. Li. 1985. Beta-endorphin-(1-27) is a naturally occurring antagonist to etorphine-induced analgesia. *Proc. Natl. Acad. Sci. USA.* 82:3178–3181.
18. Hartwig, A. C. 1991. Peripheral beta-endorphin and pain modulation. *Anesth. Prog.* 38:75–78.
19. Veening, J. G., P. O. Gerrits, and H. P. Barendregt. 2012. Volume transmission of beta-endorphin via the cerebrospinal fluid; a review. *Fluids Barriers CNS.* 9:16.
20. Chakraborty, A. K., Y. Funasaka, …, M. Ichihashi. 1996. Production and release of proopiomelanocortin (POMC) derived peptides by human melanocytes and keratinocytes in culture: regulation by ultraviolet B. *Biochim. Biophys. Acta.* 1313:130–138.
21. Slominski, A. 1998. Identification of beta-endorphin, alpha-MSH and ACTH peptides in cultured human melanocytes, melanoma and squamous cell carcinoma cells by RP-HPLC. *Exp. Dermatol.* 7:213–216.
22. Kieffer, B. L., and C. Gavériaux-Ruff. 2002. Exploring the opioid system by gene knockout. *Prog. Neurobiol.* 66:285–306.
23. Vaanholt, L. M., F. W. Turek, and P. Meerlo. 2003. Beta-endorphin modulates the acute response to a social conflict in male mice but does not play a role in stress-induced changes in sleep. *Brain Res.* 978:169–176.
24. Zakarian, S., and D. G. Smyth. 1982. Distribution of beta-endorphin-related peptides in rat pituitary and brain. *Biochem. J.* 202:561–571.
25. Loh, H. H., L. F. Tseng, …, C. H. Li. 1976. beta-endorphin is a potent analgesic agent. *Proc. Natl. Acad. Sci. USA.* 73:2895–2898.
26. Seuring, C., J. Verasdonck, …, R. Riek. 2020. The three-dimensional structure of human β-endorphin amyloid fibrils. *Nat. Struct. Mol. Biol.* 27:1178–1184.
27. Dannies, P. S. 2001. Concentrating hormones into secretory granules: layers of control. *Mol. Cell. Endocrinol.* 177:87–93.
28. Nespovitaya, N., P. Mahou, …, C. F. Kaminski. 2017. Heparin acts as a structural component of β-endorphin amyloid fibrils rather than a simple aggregation promoter. *Chem. Commun.* 53:1273–1276.
29. Paroutis, P., N. Touret, and S. Grinstein. 2004. The pH of the Secretory Pathway: Measurement, Determinants, and Regulation. *Physiology.* 19:207–215.
30. Liu, Y., Y. Zhang, …, F. Ding. 2021. A buried glutamate in the cross-β core renders β-endorphin fibrils reversible. *Nanoscale.* 13:19593–19603.
31. Pilozzi, A., C. Carro, and X. Huang. 2020. Roles of β-Endorphin in Stress, Behavior, Neuroinflammation, and Brain Energy Metabolism. *Int. J. Mol. Sci.* 22:338.
32. Hampel, H., J. Hardy, …, A. Vergallo. 2021. The Amyloid-β Pathway in Alzheimer's Disease. *Mol. Psychiatr.* 26:5481–5503.
33. Nunan, J., and D. H. Small. 2000. Regulation of APP cleavage by alpha-beta- and gamma-secretases. *FEBS Lett.* 483:6–10.
34. Lambert, M. P., A. K. Barlow, …, W. L. Klein. 1998. Diffusible, nonfibrillar ligands derived from Abeta1-42 are potent central nervous system neurotoxins. *Proc. Natl. Acad. Sci. USA.* 95:6448–6453.
35. Ferrone, F. 1999. Analysis of protein aggregation kinetics. *Methods Enzymol.* 309:256–274.
36. Morris, A. M., M. A. Watzky, and R. G. Finke. 2009. Protein aggregation kinetics, mechanism, and curve-fitting: a review of the literature. *Biochim. Biophys. Acta.* 1794:375–397.
37. Almeida, Z. L., and R. M. M. Brito. 2020. Structure and Aggregation Mechanisms in Amyloids. *Molecules.* 25:1195.
38. Arosio, P., T. P. J. Knowles, and S. Linse. 2015. On the lag phase in amyloid fibril formation. *Phys. Chem. Chem. Phys.* 17:7606–7618.
39. Harper, J. D., and P. T. Lansbury, Jr. 1997. Models of amyloid seeding in Alzheimer's disease and scrapie: mechanistic truths and physiological consequences of the time-dependent solubility of amyloid proteins. *Annu. Rev. Biochem.* 66:385–407.
40. O'Nuallain, B., S. Shivaprasad, …, R. Wetzel. 2005. Thermodynamics of A beta(1-40) amyloid fibril elongation. *Biochemistry.* 44:12709–12718.
41. Hoshi, M., M. Sato, …, K. Sato. 2003. Spherical aggregates of beta-amyloid (amylospheroid) show high neurotoxicity and activate tau protein kinase I/glycogen synthase kinase-3beta. *Proc. Natl. Acad. Sci. USA.* 100:6370–6375.
42. Barghorn, S., V. Nimmrich, …, H. Hillen. 2005. Globular amyloid beta-peptide oligomer - a homogenous and stable neuropathological protein in Alzheimer's disease. *J. Neurochem.* 95:834–847.
43. Noguchi, A., S. Matsumura, …, M. Hoshi. 2009. Isolation and characterization of patient-derived, toxic, high mass amyloid beta-protein (Abeta) assembly from Alzheimer disease brains. *J. Biol. Chem.* 284:32895–32905.
44. Kayed, R., A. Pensalfini, …, C. Glabe. 2009. Annular protofibrils are a structurally and functionally distinct type of amyloid oligomer. *J. Biol. Chem.* 284:4230–4237.

45. Stroud, J. C., C. Liu, …, D. Eisenberg. 2012. Toxic fibrillar oligomers of amyloid-β have cross-β structure. *Proc. Natl. Acad. Sci. USA.* 109:7717–7722.

46. Wu, J. W., L. Breydo, …, C. Glabe. 2010. Fibrillar oligomers nucleate the oligomerization of monomeric amyloid beta but do not seed fibril formation. *J. Biol. Chem.* 285:6071–6079.

47. Österlund, N., R. Moons, …, A. Gräslund. 2019. Native Ion Mobility-Mass Spectrometry Reveals the Formation of β-Barrel Shaped Amyloid-β Hexamers in a Membrane-Mimicking Environment. *J. Am. Chem. Soc.* 141:10440–10450.

48. Ciudad, S., E. Puig, …, N. Carulla. 2020. Aβ(1-42) tetramer and octamer structures reveal edge conductivity pores as a mechanism for membrane damage. *Nat. Commun.* 11:3014.

49. Chen, S. W., S. Drakulic, …, N. Cremades. 2015. Structural characterization of toxic oligomers that are kinetically trapped during α--synuclein fibril formation. *Proc. Natl. Acad. Sci. USA.* 112:E1994–E2003.

50. Gallea, J. I., and M. S. Celej. 2014. Structural insights into amyloid oligomers of the Parkinson disease-related protein α-synuclein. *J. Biol. Chem.* 289:26733–26742.

51. Lorenzen, N., S. B. Nielsen, …, D. E. Otzen. 2014. The role of stable α-synuclein oligomers in the molecular events underlying amyloid formation. *J. Am. Chem. Soc.* 136:3859–3868.

52. Cremades, N., S. I. A. Cohen, …, D. Klenerman. 2012. Direct observation of the interconversion of normal and toxic forms of α--synuclein. *Cell.* 149:1048–1059.

53. Buchanan, L. E., E. B. Dunkelberger, …, M. T. Zanni. 2013. Mechanism of IAPP amyloid fibril formation involves an intermediate with a transient beta-sheet. *Proc. Natl. Acad. Sci. USA.* 110:19285–19290.

54. Rodriguez Camargo, D. C., K. J. Korshavn, …, A. Ramamoorthy. 2017. Stabilization and structural analysis of a membrane-associated hIAPP aggregation intermediate. *eLife.* 6:e31226.

55. Chien, P., and J. S. Weissman. 2001. Conformational diversity in a yeast prion dictates its seeding specificity. *Nature.* 410:223–227.

56. Serio, T. R., and S. L. Lindquist. 1999. [PSI+]: an epigenetic modulator of translation termination efficiency. *Annu. Rev. Cell Dev. Biol.* 15:661–703.

57. Fowler, D. M., A. V. Koulov, …, J. W. Kelly. 2006. Functional Amyloid Formation within Mammalian Tissue. *PLoS Biol.* 4:e6.

58. Hoashi, T., H. Watabe, …, V. J. Hearing. 2005. MART-1 is required for the function of the melanosomal matrix protein PMEL17/GP100 and the maturation of melanosomes. *J. Biol. Chem.* 280:14006–14016.

59. Westermark, P., A. Andersson, and G. T. Westermark. 2011. Islet amyloid polypeptide, islet amyloid, and diabetes mellitus. *Physiol. Rev.* 91:795–826.

60. Nguyen, P. H., A. Ramamoorthy, …, P. Derreumaux. 2021. Amyloid Oligomers: A Joint Experimental/Computational Perspective on Alzheimer's Disease, Parkinson's Disease, Type II Diabetes, and Amyotrophic Lateral Sclerosis. *Chem. Rev.* 121:2545–2647.

61. Okumura, H. 2023. Perspective for Molecular Dynamics Simulation Studies of Amyloid-β Aggregates. *J. Phys. Chem. B.* 127:10931–10940.

62. Fatafta, H., M. Khaled, …, B. Strodel. 2024. A brief history of amyloid aggregation simulations. *WIREs Comput. Mol. Sci.* 14:e1703.

63. Samantray, S., and B. Strodel. 2021. The Effects of Different Glycosaminoglycans on the Structure and Aggregation of the Amyloid-β (16–22) Peptide. *J. Phys. Chem. B.* 125:5511–5525.

64. Kawecki, G. E., K. M. King, …, A. M. Brown. 2022. Simulations of cross-amyloid aggregation of amyloid-β and islet amyloid polypeptide fragments. *Biophys. J.* 121:2002–2013.

65. Itoh, S. G., and H. Okumura. 2021. Promotion and Inhibition of Amyloid-β Peptide Aggregation: Molecular Dynamics Studies. *Int. J. Mol. Sci.* 22:1859.

66. Samantray, S., F. Yin, …, B. Strodel. 2020. Different Force Fields Give Rise to Different Amyloid Aggregation Pathways in Molecular Dynamics Simulations. *J. Chem. Inf. Model.* 60:6462–6475.

67. King, K. M., A. K. Sharp, …, J. A. Lemkul. 2021. Impact of Electronic Polarization on Preformed, β-Strand Rich Homogenous and Heterogeneous Amyloid Oligomers. *J. Comput. Biophys. Chem.* 21:449–460.

68. Laganowsky, A., C. Liu, …, D. Eisenberg. 2012. Atomic view of a toxic amyloid small oligomer. *Science.* 335:1228–1231.

69. McKnelly, K. J., A. G. Kreutzer, …, J. S. Nowick. 2022. Effects of Familial Alzheimer's Disease Mutations on the Assembly of a β--Hairpin Peptide Derived from Aβ16–36. *Biochemistry.* 61:446–454.

70. Brown, A. M., and D. R. Bevan. 2016. Molecular Dynamics Simulations of Amyloid β-Peptide (1-42): Tetramer Formation and Membrane Interactions. *Biophys. J.* 111:937–949.

71. Barz, B., Q. Liao, and B. Strodel. 2018. Pathways of Amyloid-β Aggregation Depend on Oligomer Shape. *J. Am. Chem. Soc.* 140:319–327.

72. Ladiwala, A. R. A., J. Litt, …, P. M. Tessier. 2012. Conformational differences between two amyloid β oligomers of similar size and dissimilar toxicity. *J. Biol. Chem.* 287:24765–24773.

73. Iglesias, V., J. Chilimoniuk, …, M. Burdukiewicz. 2024. Aggregating amyloid resources: A comprehensive review of databases on amyloid-like aggregation. *Comput. Struct. Biotechnol. J.* 23:4011–4018.

74. Graña-Montes, R., J. Pujols-Pujol, …, S. Ventura. 2017. Prediction of Protein Aggregation and Amyloid Formation. *In* From Protein Structure to Function with Bioinformatics. D. J. Rigden, ed Springer, pp. 205–263.

75. Ventura, S., J. Zurdo, …, L. Serrano. 2004. Short amino acid stretches can mediate amyloid formation in globular proteins: The Src homology 3 (SH3) case. *Proc. Natl. Acad. Sci. USA.* 101:7258–7263.

76. Van Der Spoel, D., E. Lindahl, …, H. J. C. Berendsen. 2005. GROMACS: Fast, flexible, and free. *J. Comput. Chem.* 26:1701–1718.

77. Abraham, M. J., T. Murtola, …, E. Lindahl. 2015. GROMACS: High performance molecular simulations through multi-level parallelism from laptops to supercomputers. *SoftwareX.* 1–2:19–25.

78. Oostenbrink, C., A. Villa, …, W. F. van Gunsteren. 2004. A biomolecular force field based on the free enthalpy of hydration and solvation: the GROMOS force-field parameter sets 53A5 and 53A6. *J. Comput. Chem.* 25:1656–1676.

79. Gerben, S. R., J. A. Lemkul, …, D. R. Bevan. 2014. Comparing atomistic molecular mechanics force fields for a difficult target: a case study on the Alzheimer's amyloid β-peptide. *J. Biomol. Struct. Dyn.* 32:1817–1832.

80. Berendsen, H., J. P. M. Postma, …, J. Hermans. 1981. Interaction Models for Water in Relation to Protein Hydration, 11, pp. 331–342.

81. Berendsen, H. J. C., J. P. M. Postma, J. R. Haak…, 1984. Molecular-dynamics with coupling to an external bath. *J. Chem. Phys.* 81:3684–3690.

82. Hoover, W. G. 1985. Canonical dynamics: Equilibrium phase-space distributions. *Phys. Rev.* 31:1695–1697.

83. Parrinello, M. R. A., and A. J. Rahman. 1982. Polymorphic Transitions in Single Crystals: A New Molecular Dynamics Method, 52, pp. 7182–7190.

84. Hess, B., H. Bekker, …, G. E. M. Fraaije. 1998. LINCS: A Linear Constraint Solver for Molecular Simulations, 18.

85. Darden, T., D. York, and L. Pedersen. 1993. Particle Mesh Ewald: An Nlog (N) Method for Ewald Sums in Large Systems, 98, pp. 10089–10092.

86. Essmann, U., L. Perera, …, L. G. Pedersen. 1995. A smooth particle mesh Ewald method. *J. Chem. Phys.* 103:8577–8593.

87. Crescenzi, O., S. Tomaselli, …, D. Picone. 2002. Solution structure of the Alzheimer amyloid β-peptide (1–42) in an apolar microenvironment. *Eur. J. Biochem.* 269:5642–5648.

88. The PyMOL Molecular Graphics System. Version 2.0 Schrödinger, LLC.

89. Brown, A. M., J. Briganti, …, A. D. Prendergast. 2025. Brown Lab, Public. OSF. https://doi.org/10.17605/OSF.IO/82N73.

90. Kyte, J., and R. F. Doolittle. 1982. A simple method for displaying the hydropathic character of a protein. *J. Mol. Biol.* 157:105–132.

91. Yu, B., and Y. Zhang. 2013. A simple method for predicting transmembrane proteins based on wavelet transform. *Int. J. Biol. Sci.* 9:22–33.

92. McGibbon, R. T., K. A. Beauchamp, …, V. S. Pande. 2015. MDTraj: A Modern Open Library for the Analysis of Molecular Dynamics Trajectories. *Biophys. J.* 109:1528–1532.

93. Kabsch, W., and C. Sander. 1983. Dictionary of protein secondary structure: pattern recognition of hydrogen-bonded and geometrical features. *Biopolymers.* 22:2577–2637.

94. Daura, X., W. F. van Gunsteren, and A. E. Mark. 1999. Folding–unfolding thermodynamics of a β-heptapeptide from equilibrium simulations. *Proteins.* 34:269–280.

95. Virtanen, P., R. Gommers, …, C. SciPy. 2020. SciPy 1.0: fundamental algorithms for scientific computing in Python. *Nat. Methods.* 17:261–272.

96. Larsen, P., J. L. Nielsen, …, P. H. Nielsen. 2007. Amyloid adhesins are abundant in natural biofilms. *Environ. Microbiol.* 9:3077–3090.

97. Garcia-Sherman, M. C., T. Lundberg, …, S. A. Klotz. 2015. A unique biofilm in human deep mycoses: fungal amyloid is bound by host serum amyloid P component. *NPJ Biofilms Microbiomes.* 1:15009.

98. Maury, C. P. J. 2009. The emerging concept of functional amyloid. *J. Intern. Med.* 265:329–334.

99. Scollo, F., C. Tempra, …, C. La Rosa. 2018. Phospholipids Critical Micellar Concentrations Trigger Different Mechanisms of Intrinsically Disordered Proteins Interaction with Model Membranes. *J. Phys. Chem. Lett.* 9:5125–5129.

100. Mukhopadhyay, S. 2020. The Dynamism of Intrinsically Disordered Proteins: Binding-Induced Folding, Amyloid Formation, and Phase Separation. *J. Phys. Chem. B.* 124:11541–11560.

101. Xie, H., S. Vucetic, …, Z. Obradovic. 2007. Functional anthology of intrinsic disorder. 1. Biological processes and functions of proteins with long disordered regions. *J. Proteome Res.* 6:1882–1898.

102. Hukushima, K., and K. Nemoto. 1996. Exchange Monte Carlo Method and Application to Spin Glass Simulations. *J. Physical Soc. Japan.* 65:1604–1608.

103. Sugita, Y., and Y. Okamoto. 1999. Replica-exchange molecular dynamics method for protein folding. *Chem. Phys. Lett.* 314:141–151.

104. Miao, Y., and J. A. McCammon. 2017. Chapter Six - Gaussian Accelerated Molecular Dynamics: Theory, Implementation, and Applications. *In* Annu. Rep. Comput. Chem., *13* D. A. Dixon, ed., pp. 231–278, Elsevier.

105. Gracia Carmona, O., and C. Oostenbrink. 2023. Flexible Gaussian Accelerated Molecular Dynamics to Enhance Biological Sampling. *J. Chem. Theor. Comput.* 19:6521–6531.

106. Sgourakis, N. G., Y. Yan, …, A. E. Garcia. 2007. The Alzheimer's peptides Abeta40 and 42 adopt distinct conformations in water: a combined MD/NMR study. *J. Mol. Biol.* 368:1448–1457.

107. Khaled, M., I. Rönnbäck, …, N. Österlund. 2023. A Hairpin Motif in the Amyloid-β Peptide Is Important for Formation of Disease-Related Oligomers. *J. Am. Chem. Soc.* 145:18340–18354.

108. Itoh, S. G., and H. Okumura. 2014. Dimerization Process of Amyloid-β(29–42) Studied by the Hamiltonian Replica-Permutation Molecular Dynamics Simulations. *J. Phys. Chem. B.* 118:11428–11436.

109. Abelein, A., J. P. Abrahams, …, S. K. T. S. Wärmländer. 2014. The hairpin conformation of the amyloid β peptide is an important structural motif along the aggregation pathway. *J. Biol. Inorg. Chem.* 19:623–634.

110. Maity, S., M. Hashemi, and Y. L. Lyubchenko. 2017. Nano-assembly of amyloid β peptide: role of the hairpin fold. *Sci. Rep.* 7:2344.

111. Mazargui, H., C. Lévêque, …, L. Perrone. 2012. A synthetic amino acid substitution of Tyr10 in Aβ peptide sequence yields a dominant negative variant in amyloidogenesis. *Aging Cell.* 11:530–541.

112. Sun, Y., N. Andrikopoulos, …, F. Ding. 2025. Formation of a β--Endorphin Corona Mitigates Alzheimer's Amyloidogenesis. *Small.* 21:2409392.

113. Kreutzer, A. G., I. L. Hamza, …, J. S. Nowick. 2016. X-ray Crystallographic Structures of a Trimer, Dodecamer, and Annular Pore Formed by an Aβ17–36 β-Hairpin. *J. Am. Chem. Soc.* 138:4634–4642.

114. Kawai, R., S. Chiba, …, Y. Okuno. 2020. Stabilization Mechanism for a Nonfibrillar Amyloid β Oligomer Based on Formation of a Hydrophobic Core Determined by Dissipative Particle Dynamics. *ACS Chem. Neurosci.* 11:385–394.

115. Itoh, S. G., M. Yagi-Utsumi, …, H. Okumura. 2022. Key Residue for Aggregation of Amyloid-β Peptides. *ACS Chem. Neurosci.* 13:3139–3151.

116. Gessel, M. M., S. Bernstein, …, M. T. Bowers. 2012. Familial Alzheimer's disease mutations differentially alter amyloid β-protein oligomerization. *ACS Chem. Neurosci.* 3:909–918.

117. Balbach, J. J., Y. Ishii, …, R. Tycko. 2000. Amyloid Fibril Formation by Aβ16-22, a Seven-Residue Fragment of the Alzheimer's β--Amyloid Peptide, and Structural Characterization by Solid State NMR. *Biochemistry.* 39:13748–13759.

118. King, K. M., D. R. Bevan, and A. M. Brown. 2022. Molecular Dynamics Simulations Indicate Aromaticity as a Key Factor in the Inhibition of IAPP(20–29) Aggregation. *ACS Chem. Neurosci.* 13:1615–1626.

119. Lorenzo, A., B. Razzaboni, …, B. A. Yankner. 1994. Pancreatic islet cell toxicity of amylin associated with type-2 diabetes mellitus. *Nature.* 368:756–760.

120. Lemkul, J. A., and D. R. Bevan. 2009. Perturbation of membranes by the amyloid beta-peptide–a molecular dynamics study. *FEBS J.* 276:3060–3075.

121. Serra-Batiste, M., M. Ninot-Pedrosa, …, N. Carulla. 2016. Aβ42 assembles into specific β-barrel pore-forming oligomers in membrane-mimicking environments. *Proc. Natl. Acad. Sci. USA.* 113:10866–10871.

122. Bode, D. C., M. Freeley, …, J. H. Viles. 2019. Amyloid-β oligomers have a profound detergent-like effect on lipid membrane bilayers, imaged by atomic force and electron microscopy. *J. Biol. Chem.* 294:7566–7572.

123. Sciacca, M. F., F. Lolicato, …, C. La Rosa. 2020. Lipid-Chaperone Hypothesis: A Common Molecular Mechanism of Membrane Disruption by Intrinsically Disordered Proteins. *ACS Chem. Neurosci.* 11:4336–4350.

124. Fauchere, J. L., and V. Pliska. 1983. Hydrophobic parameters pi of amino-acid side chains from the partitioning of N-acetyl-amino amides. *Eur. J. Med. Chem.* 4:369–375.

125. Kiskis, J., H. Fink, …, A. Enejder. 2015. Plaque-associated lipids in Alzheimer's diseased brain tissue visualized by nonlinear microscopy. *Sci. Rep.* 5:13489.

126. Liao, C. R., M. Rak, …, K. M. Gough. 2013. Synchrotron FTIR reveals lipid around and within amyloid plaques in transgenic mice and Alzheimer's disease brain. *Analyst.* 138:3991–3997.

127. Kanatsuka, A., S. Kou, and H. Makino. 2018. IAPP/amylin and β-cell failure: implication of the risk factors of type 2 diabetes. *Diabetol. Int.* 9:143–157.

Biophysical Journal, Volume *124*

Supplemental information

The role of hydrophobic collapse in cytotoxic and functional amyloid oligomerization

Kelsie M. King, Hajar Zaheer, and Anne M. Brown

Table S1. Secondary structure percentages for $A\beta_{42}$ and βE_{31} monomer simulations.

	$A\beta_{42}$ Monomer Secondary Structure (%)			βE_{31} Monomer Secondary Structure (%)		
Replicate	***Coil***	***β-Sheet***	***Helix***	***Coil***	***β-Sheet***	***Helix***
Replicate 1	59.1	40.9	0.02	65.4	34.5	0.07
Replicate 2	55.8	44.1	0.02	61.4	38.5	0.06
Replicate 3	65.8	34.2	0.07	73.4	26.5	0.16
All	60.2	39.7	0.04	66.7	33.2	0.09

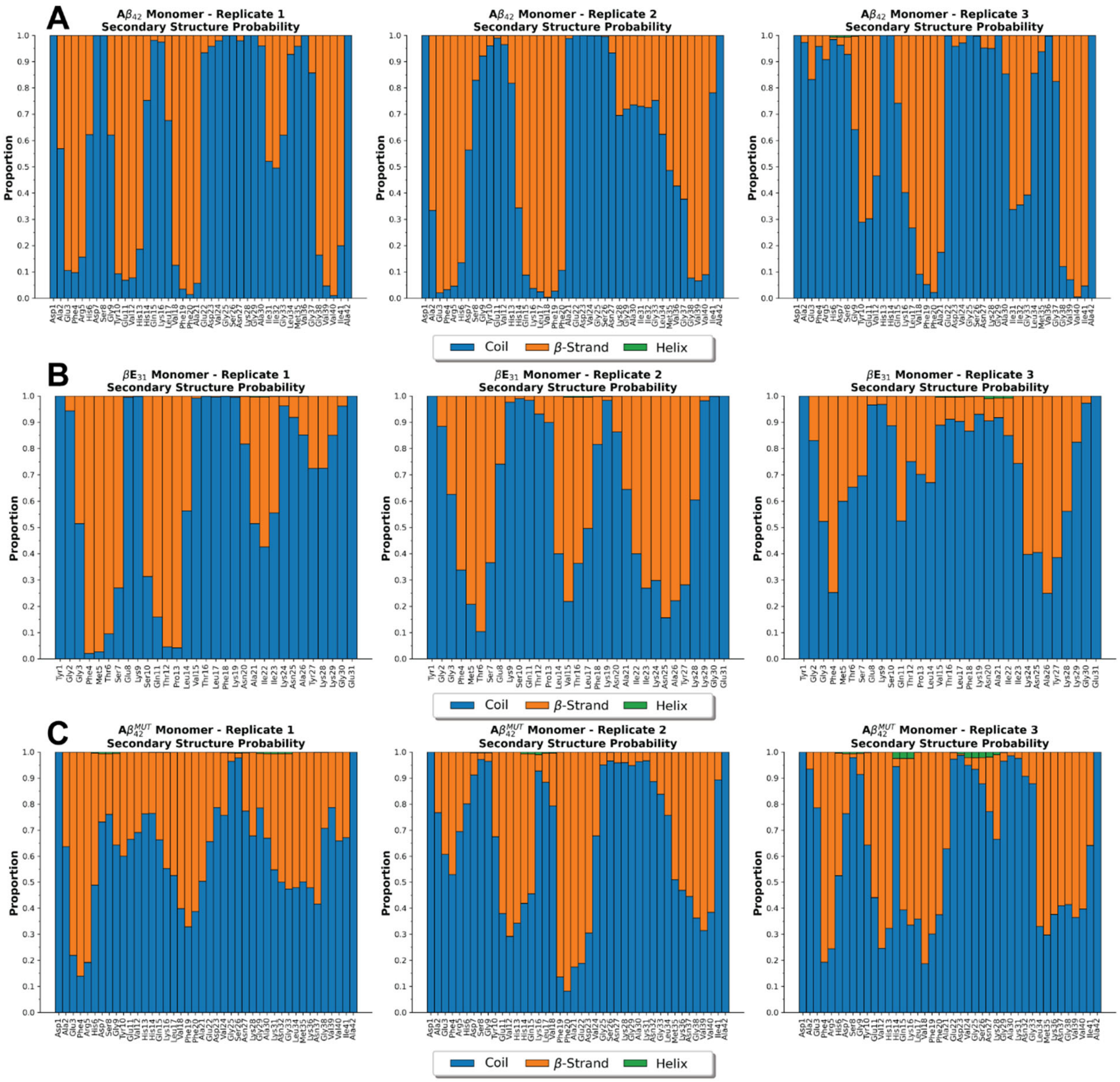

Figure S1. Secondary structure probability per residue from monomer simulations. Secondary structure probabilities for (**A**) $A\beta_{42}$, (**B**) βE_{31}, and (**C**) $A\beta_{42}^{MUT}$. Probabilities are colored as follows: coil (blue), β-strand (orange), helix (green). Probabilities calculated as percentage of frames a given residue adopts either β-strand, coil, or helical structure throughout the 2 μs simulation period for each replicate.

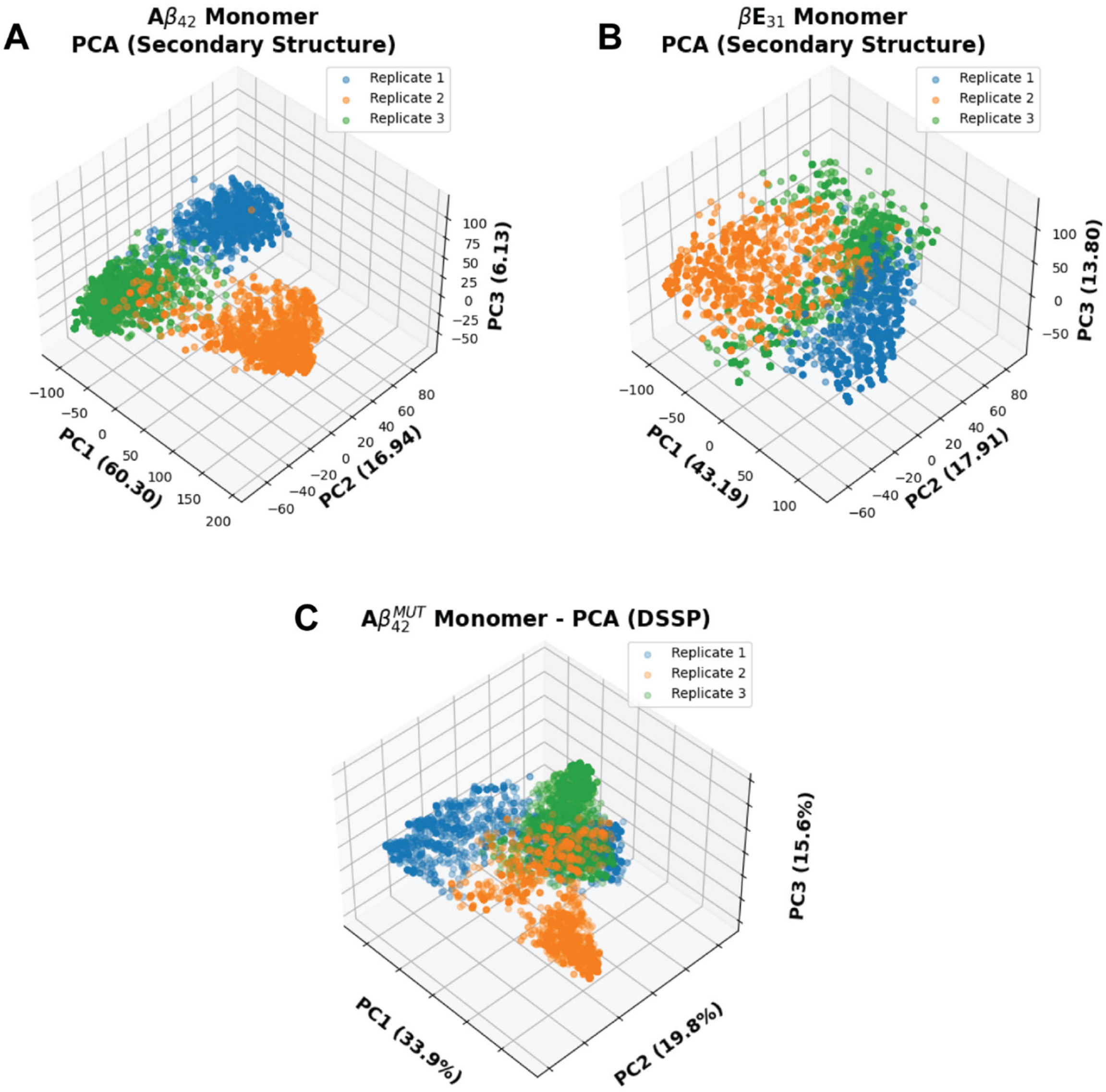

Figure S2. Principal-component analysis (PCA) of sampled secondary structure content from monomer simulations. DSSP data was down-sampled to include 2,000 frames/replicate (every 1 ns), for a total of 6,000 datapoints across all replicates, for (**A**) $A\beta_{42}$, (**B**) βE_{31}, and (**C**) $A\beta_{42}^{MUT}$.

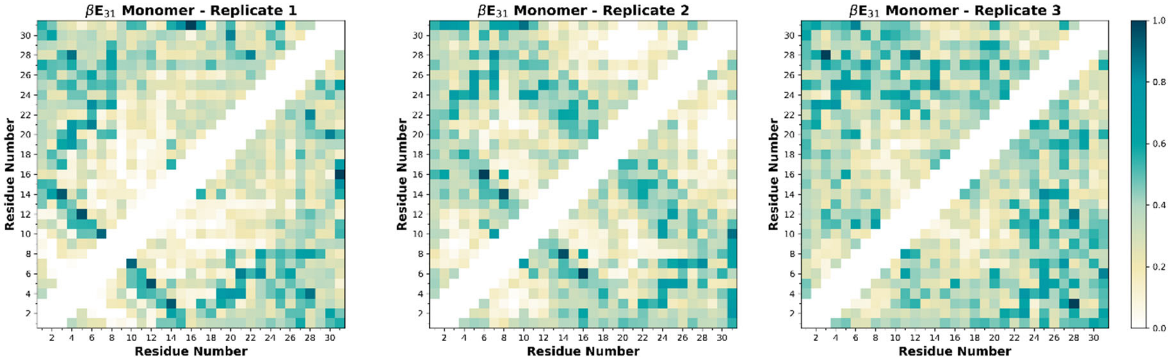

Figure S3. Intra-residue interaction probability heatmaps for βE_{31} monomer simulations. Probabilities calculated based on residue pair distance and fraction of frames where pair distance ≤ 0.6 nm, scaled between 0 and 1.

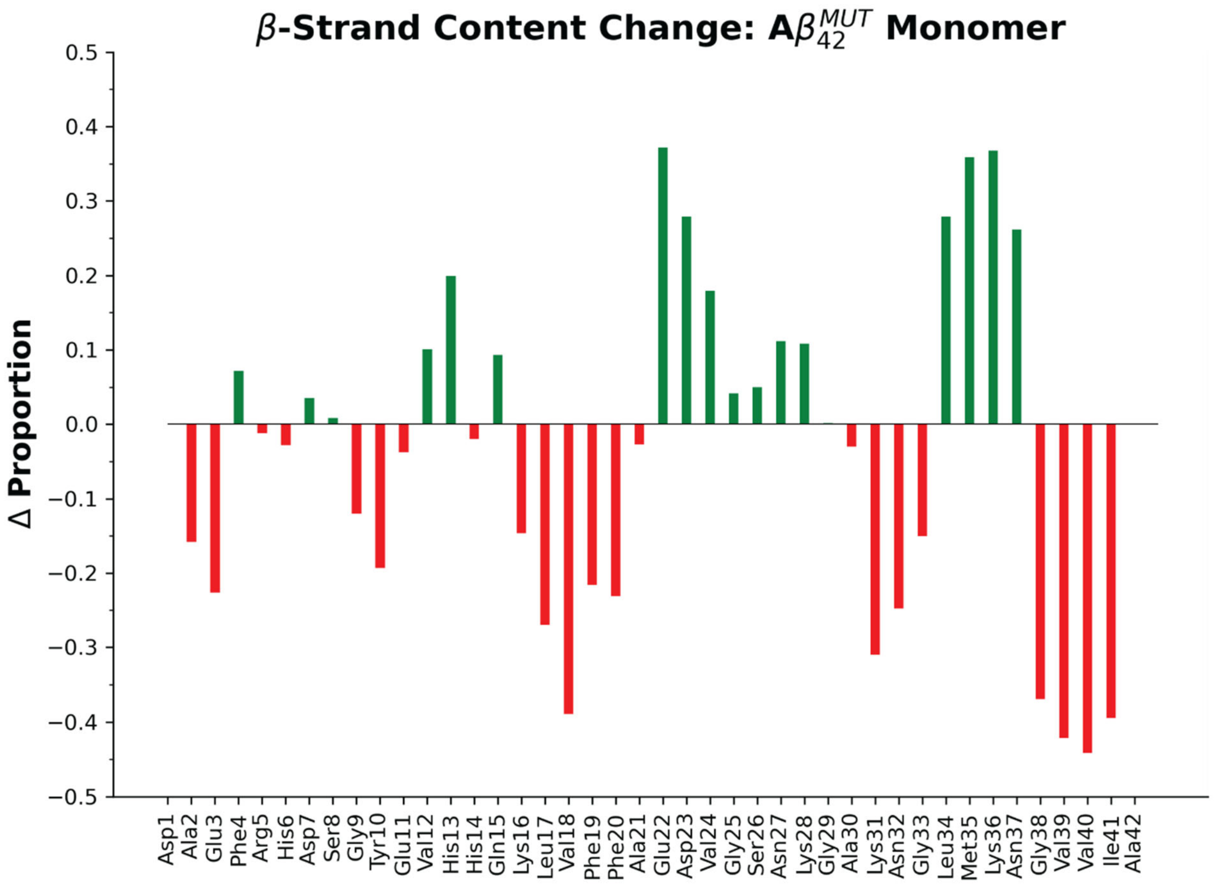

Figure S4. Change in β-strand content propensity from in monomeric $A\beta_{42}^{MUT}$ relative to monomeric $A\beta^{42}$. Green indicates an increase in β-strand probability in $A\beta_{42}^{MUT}$ relative to $A\beta_{42}$. red indicates a decrease in β-strand probability in $A\beta_{42}^{MUT}$ relative to $A\beta_{42}$. Initial probabilities calculated as percentage of frames a given residue adopts either β-strand, coil, or helical structure throughout the 2 μs simulation period over all replicates.

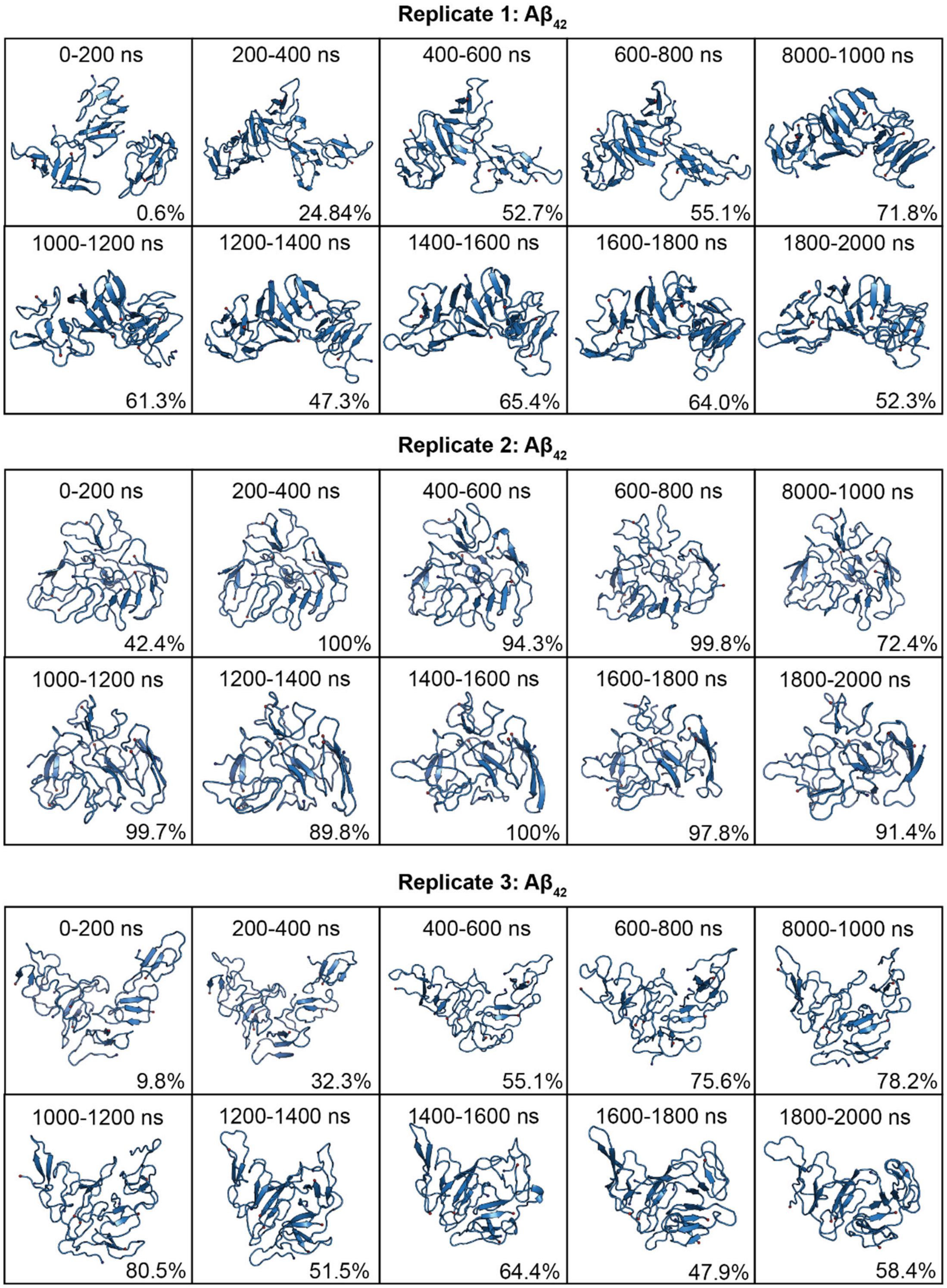

Figure S5. Representative structures of Aβ_{42} hexamers from RMSD clustering. RMSD clustering was performed over 200 ns intervals of the trajectory. Protein shown as blue cartoon. Percentages indicate the percentage of frames the structure represents over the indicated timeframe.

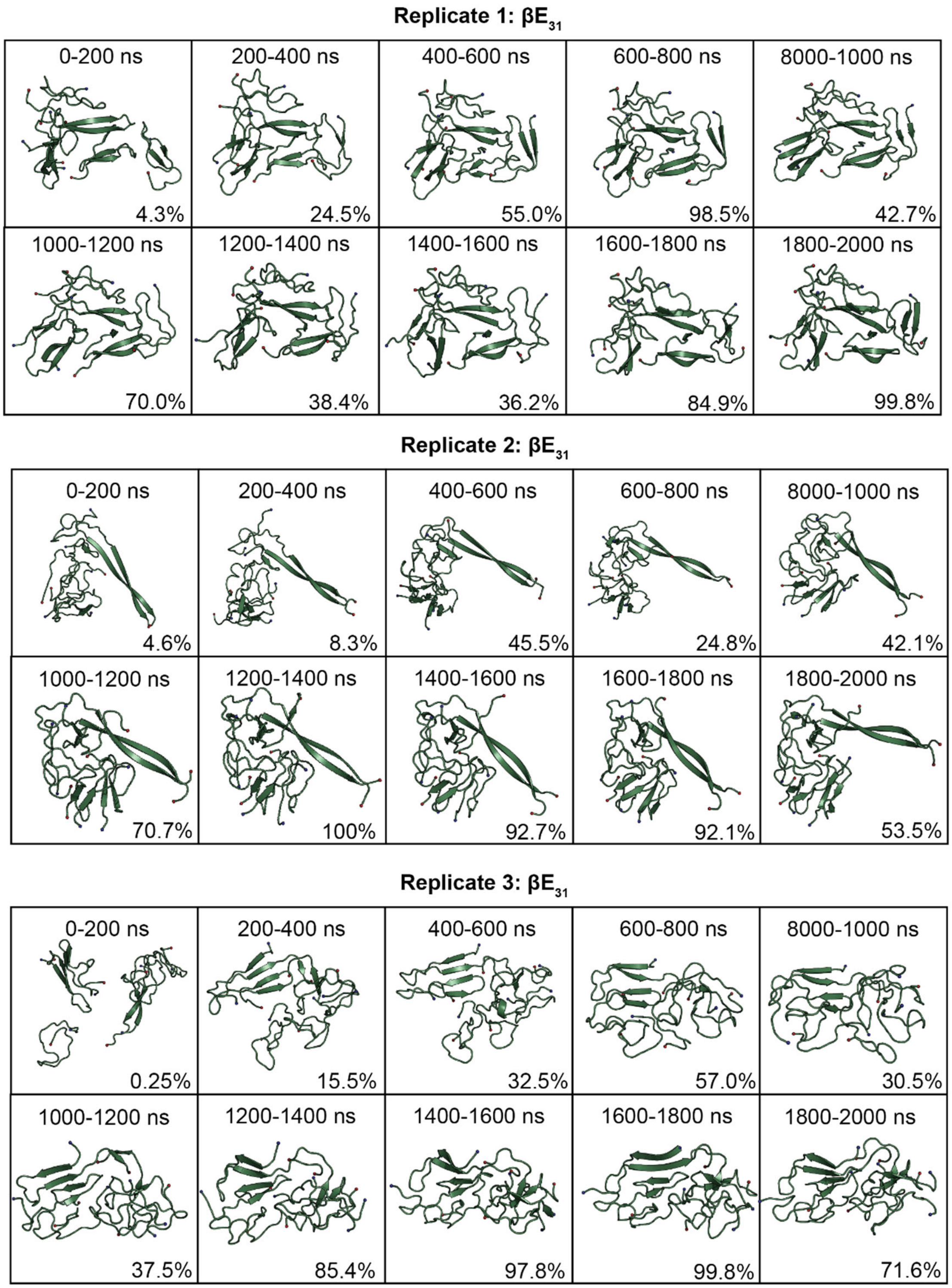

Figure S6. Representative structures of βE$_{31}$ hexamers from RMSD clustering. RMSD clustering was performed over 200 ns intervals of the trajectory. Protein shown as green cartoon. Percentages indicate the percentage of frames the structure represents over the indicated timeframe.

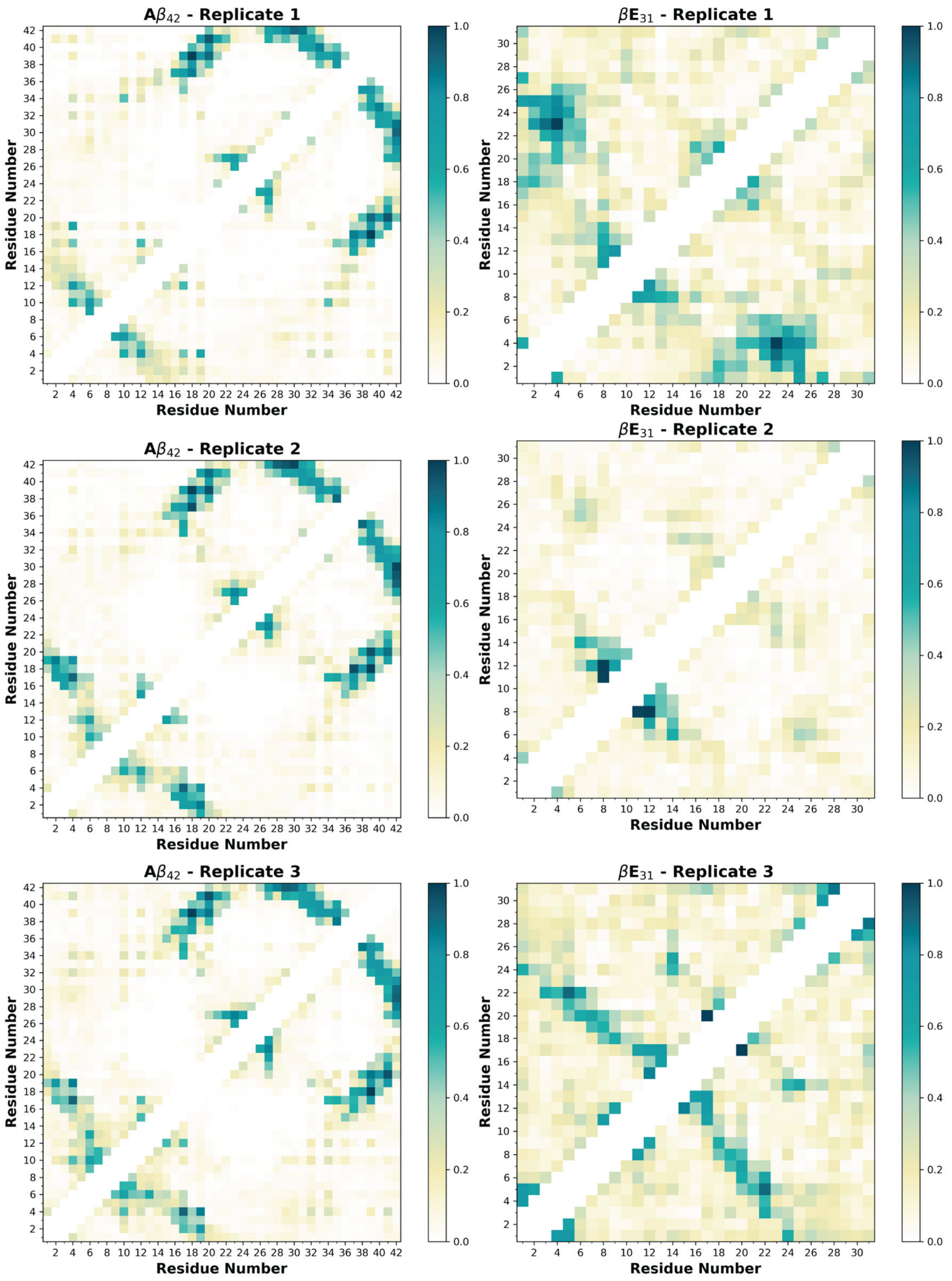

Figure S7. Intra-molecular interaction probability heatmaps by replicate Aβ$_{42}$ and βE$_{31}$ hexamer simulations. Represents weighted frequency of interaction over the 2 μs simulation period for residues on the same chain. Residue pairs within 2 residues are excluded.

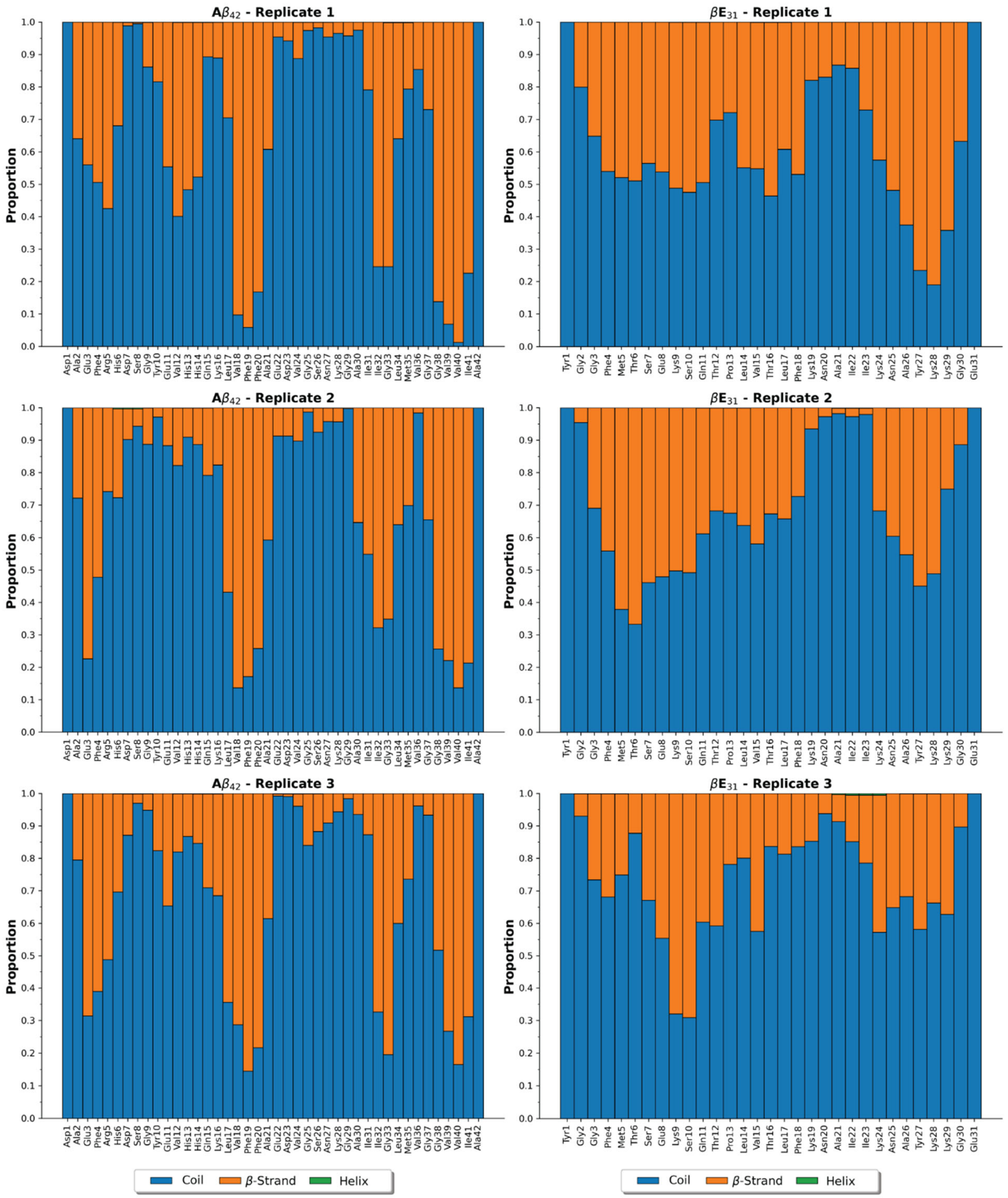

Figure S8. Secondary structure probability per residue from hexamer simulations. Secondary structure probabilities for (**left**) Aβ_{42} and (**right**) βE_{31}. Probabilities are colored as follows: coil (blue), β-strand (orange), helix (green). Probabilities calculated as percentage of frames a given residue adopts either β-strand, coil, or helical structure throughout the 2 μs simulation period for each replicate

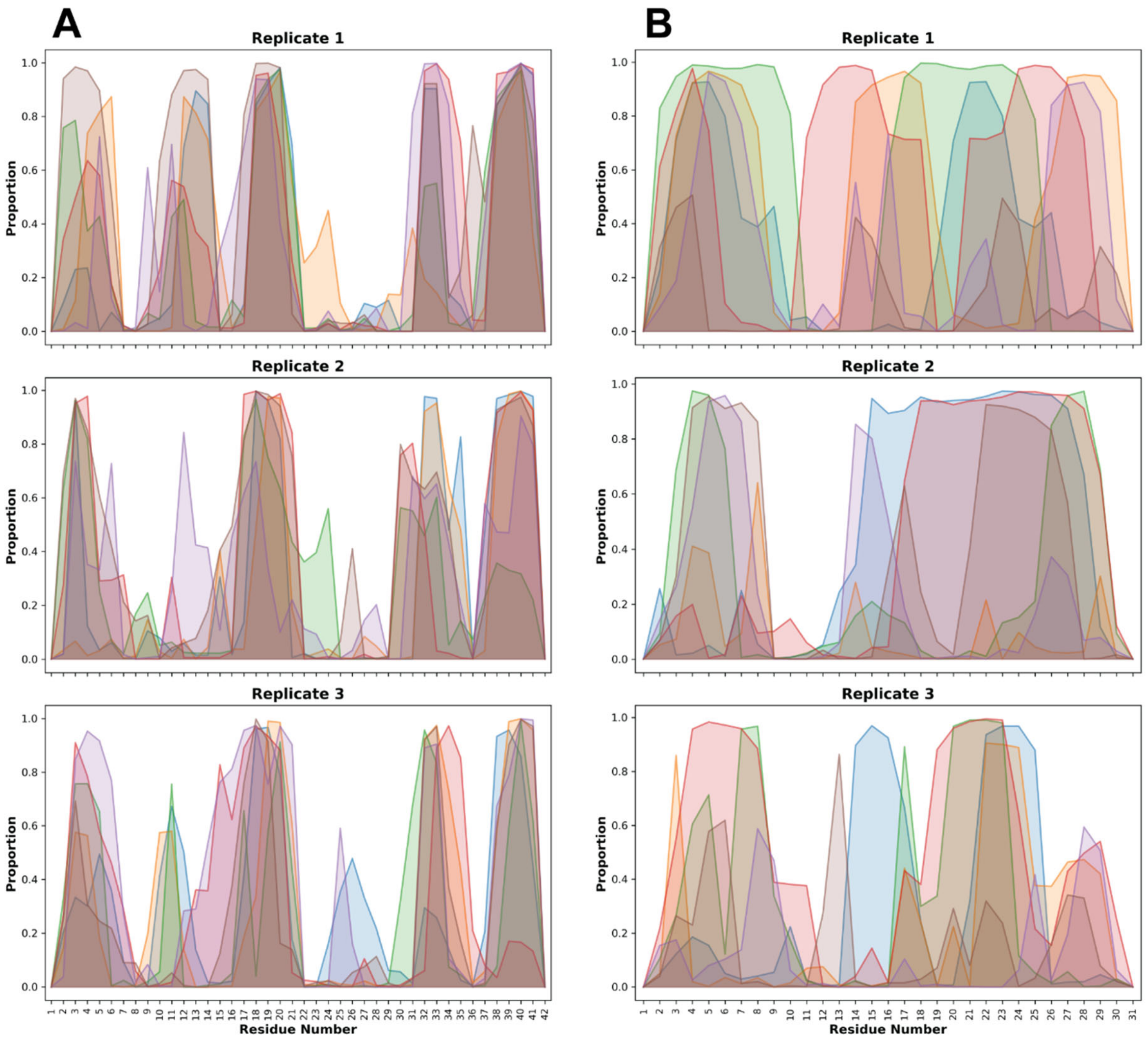

Figure S9. Proportion of frames β-strand is sampled per-residue and per-chain, for (A) Aβ_{42} and (B) βE_{31} hexamer simulations.

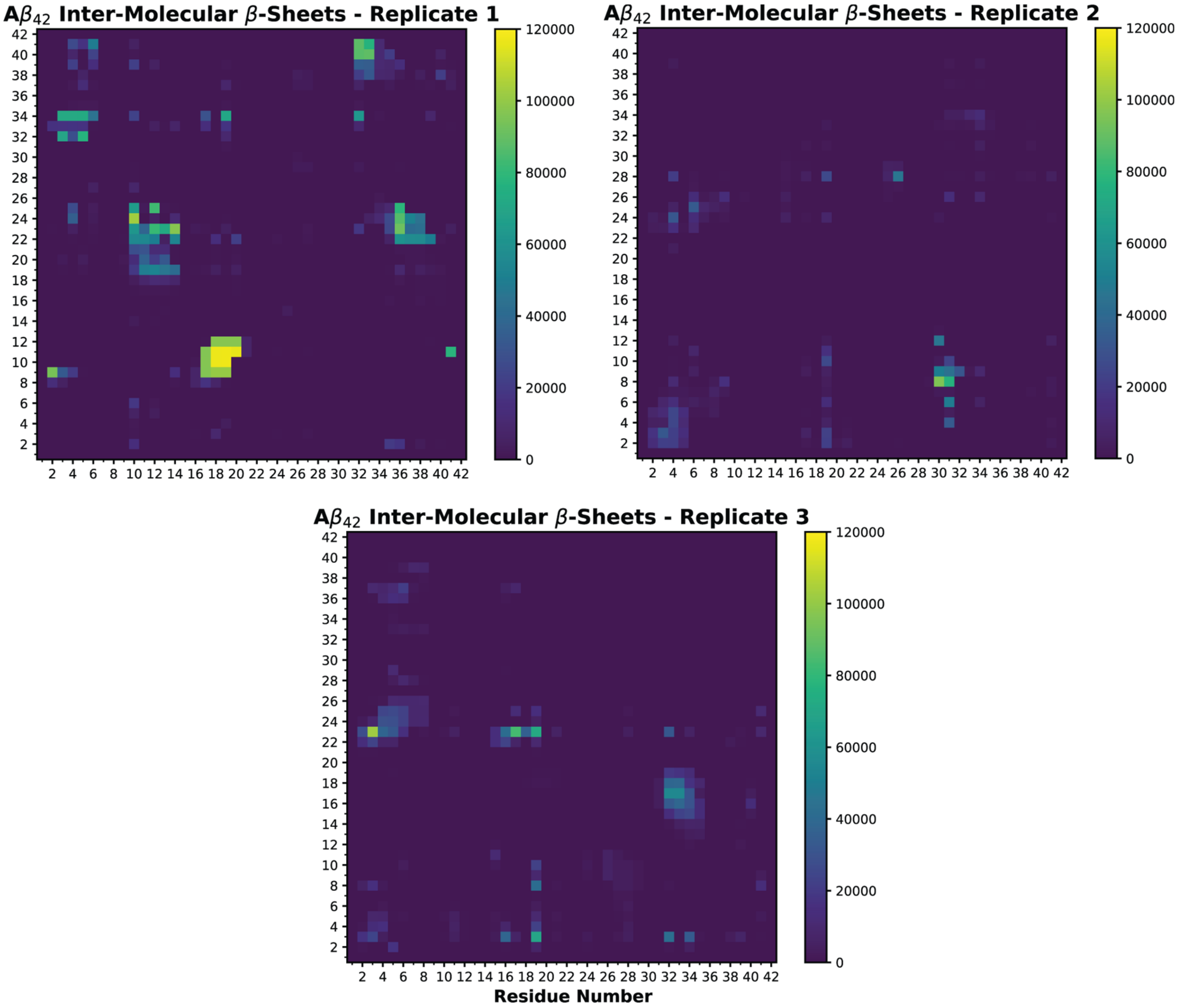

Figure S10. Intermolecular β-strand counts per residue for Aβ42 hexamer simulations. For each frame (downsampled every 10 ps), and for each residue pair, if an interaction was present ($d \leq 0.6$ nm), and both residues adopted β-strand content at that frame, the count was increased.

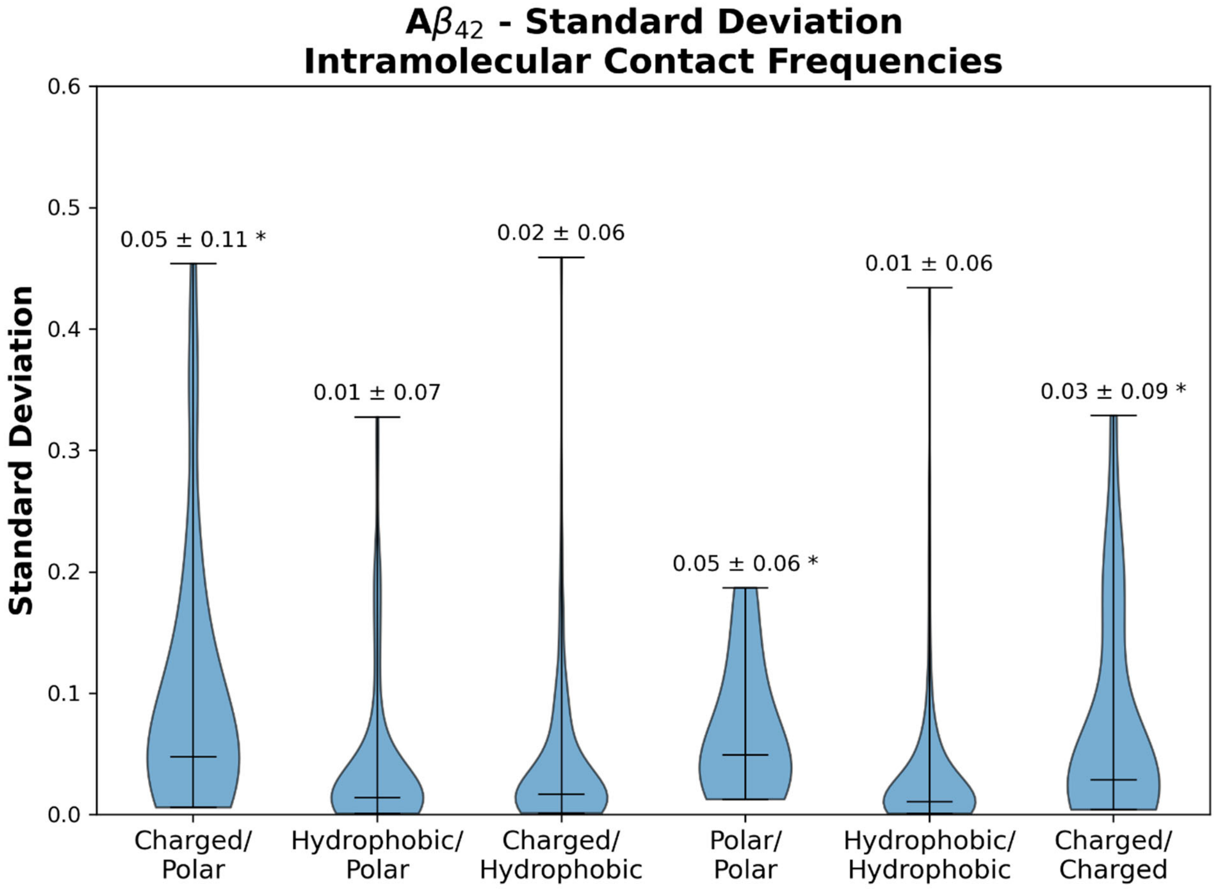

Figure S11. Standard deviations of intramolecular contact frequencies for Aβ_{42} by residue sidechain property. Asterisk (*) indicates statistically significant difference in mean to other groups ($p \leq 0.5$).

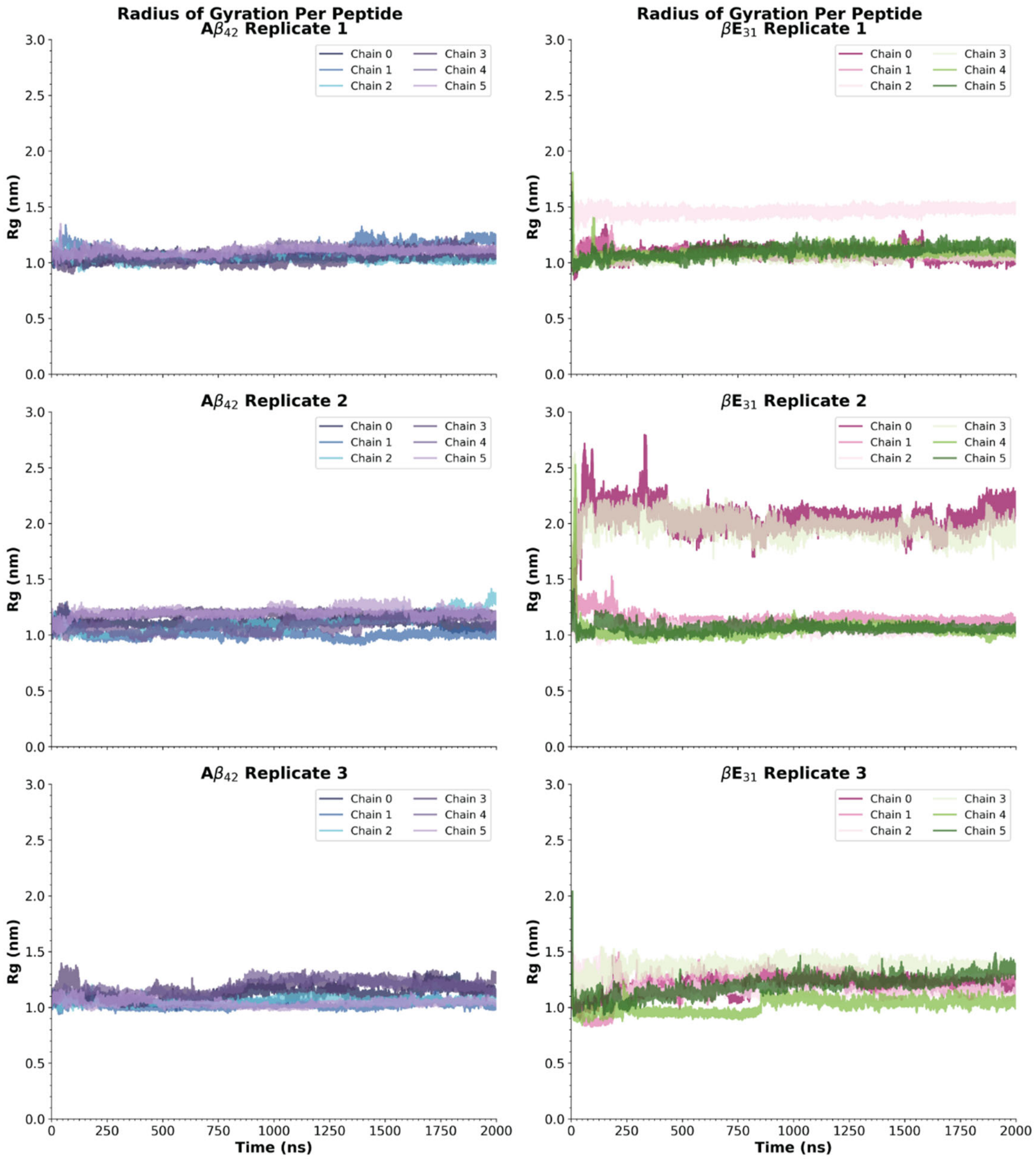

Figure S12. Radius of gyration over time per peptide for $A\beta_{42}$ and βE_{31} hexamers.

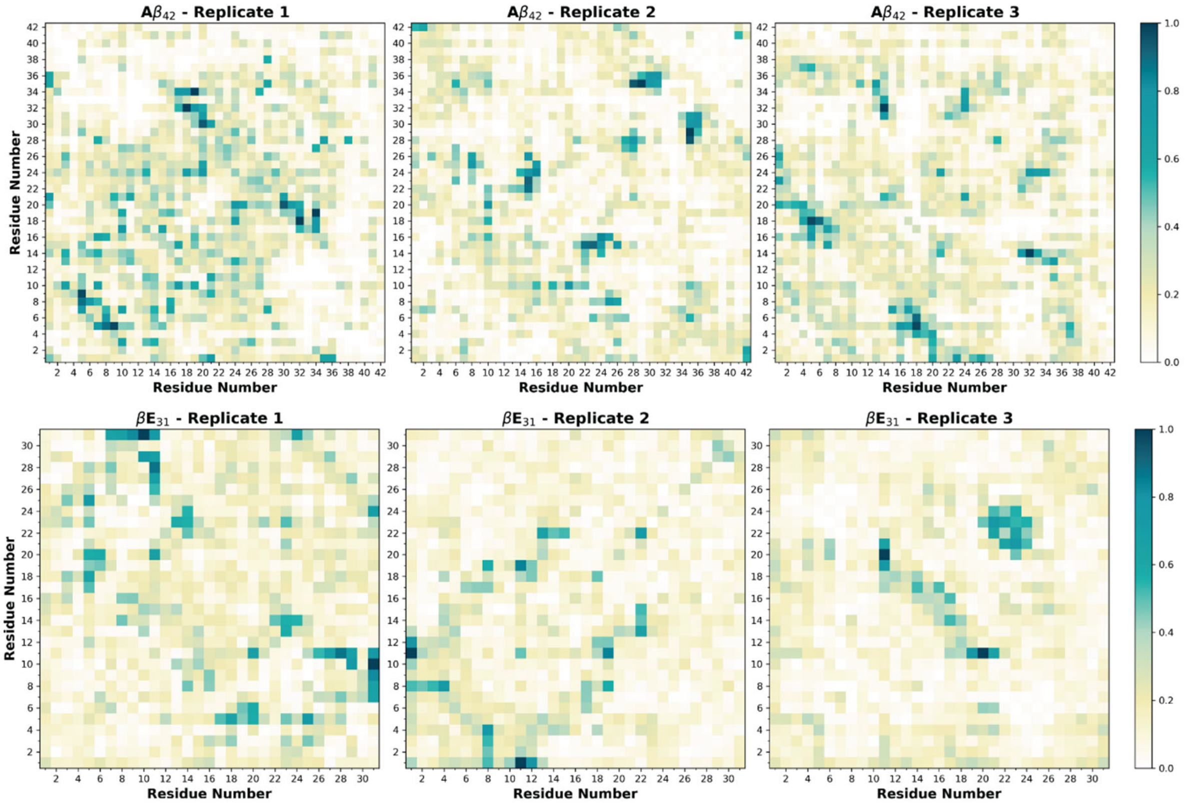

Figure S13. Inter-molecular interaction probability heatmaps by replicate Aβ_{42} and βE_{31} hexamer simulations. Represents weighted frequency of interaction over the 2 μs simulation period for residues that are not located on the same chain.

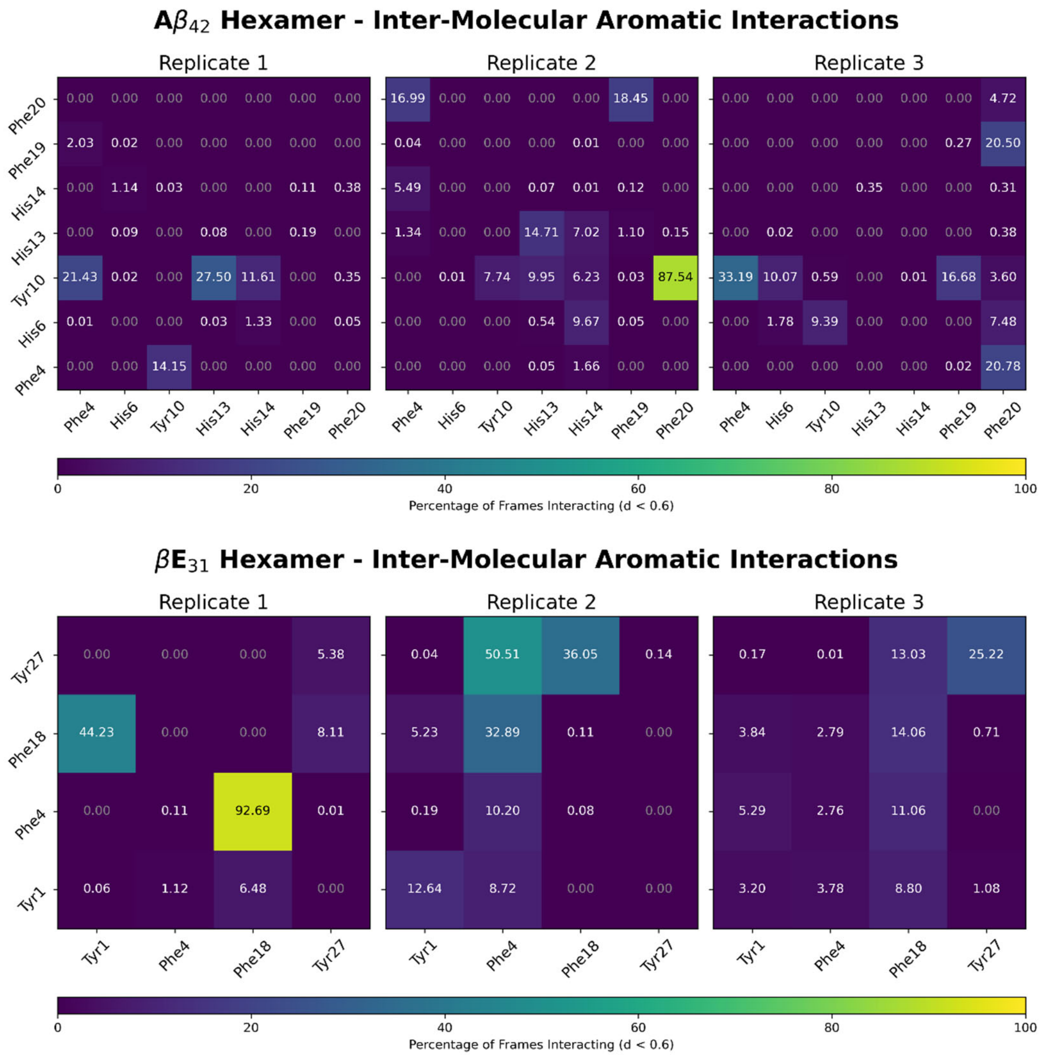

Figure S14. Inter-molecular π-stacking interaction heatmaps by replicate for (top) $A\beta_{42}$ and (bottom) βE_{31} hexamer simulations. Represents the maximum percentage of interaction between aromatic residues not located on the same chain.

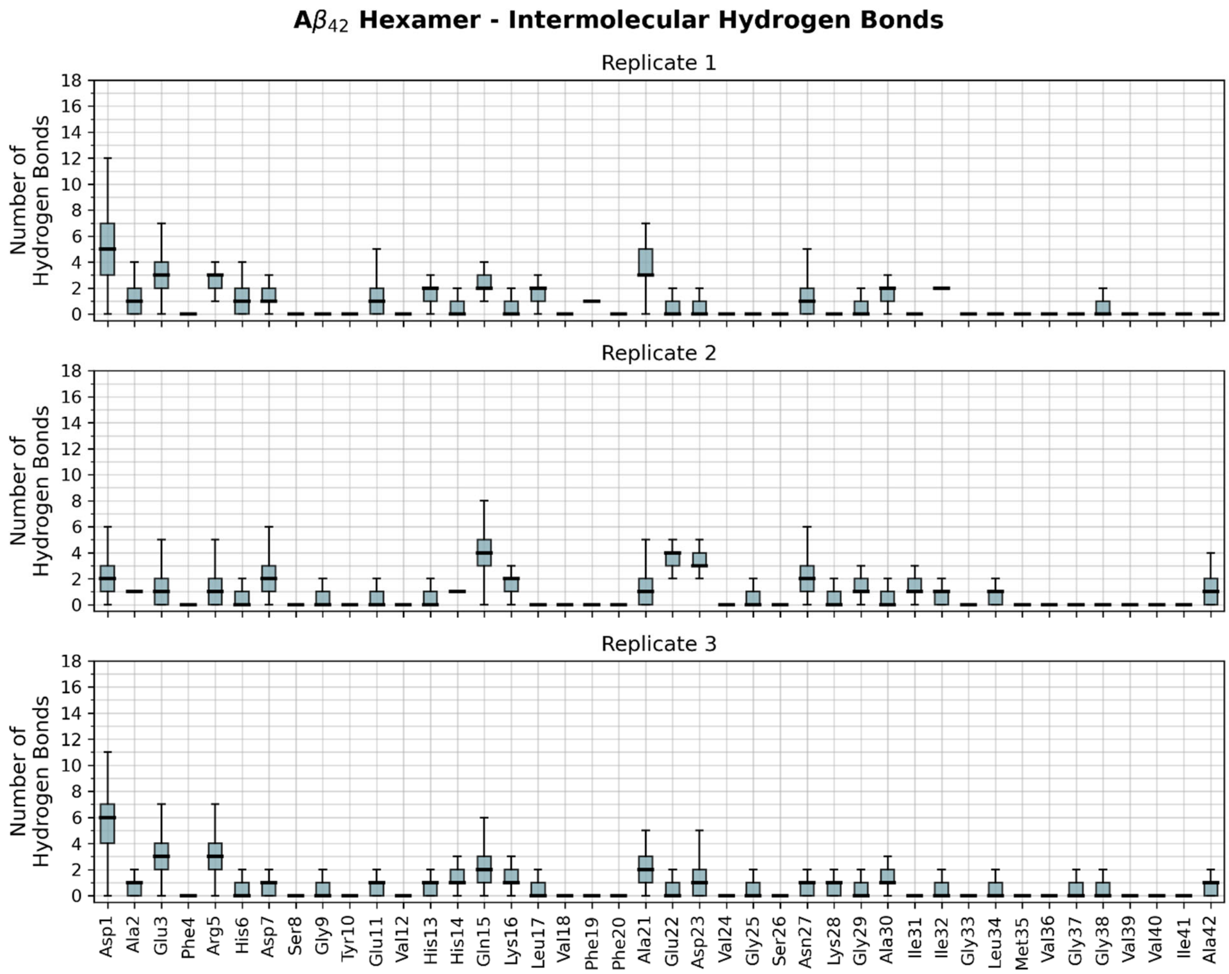

Figure S15. Inter-molecular hydrogen bonds by replicate for Aβ$_{42}$ hexamer simulations. Boxplots represent the number of hydrogens bonds each residue participates in over the 2 μs simulation period. Outliers not shown for clarity.

Table S2. Inter-molecular salt bridge interactions present in Aβ_{42} hexamer simulations.

Replicate	*Negative Residue*	*Positive Residue*	*Occupancy (%, from 0.3–2 μs)*
**1**	Asp7	Asp1 (N-term)	33.3
	Asp1	Asp1 (N-term)	32.8
**2**	Asp1	Lys28	38.5
	Ala42 (C-term)	Lys16	33.5
	Glu3	Asp1 (N-term)	15.4
	Asp7	Asp1 (N-term)	11.9
	Glu22	Asp1 (N-term)	10.2
**3**	Asp23	Asp1 (N-term)	74.3
	Asp1	Lys16	34.3
	Glu22	Asp1 (N-term)	17.8
	Glu22	Lys16	14.3
	Asp7	Lys16	12.1

Salt bridge interaction was defined as a distance ≤ 0.3 between charged atoms of charged residues not located on the same chain. Occupancy calculated as a percentage of frames present in the aggregated period of simulation (0.3-2 μs). Only salt bridges present for ≥10% of the sampled period are shown.

Table S3. Inter-molecular salt bridge interactions present in βE_{31} hexamer simulations.

Replicate	*Negative Residue*	*Positive Residue*	*Occupancy (%, from 0.3–2 μs)*
**1**	Glu31	Lys9	12.75, 16.5
	Glu31	Lys24	14.8
	Glu31	Lys28	10.7
	Glu8	Lys9	10.0
**2**	Glu8	Lys19	34.0, 44.2
	Glu31	Lys28	25.8
	Glu31	Lys29	19.2
**3**	Glu8	Lys19	19.8
	Glu31	Lys28	19.6
	Glu31	Lys24	16.5

Salt bridge interaction was defined as a distance ≤ 0.3 between charged atoms of charged residues not located on the same chain. Occupancy calculated as a percentage of frames present in the aggregated period of simulation (0.3-2 μs). Only salt bridges present for ≥10% of the sampled period are shown.

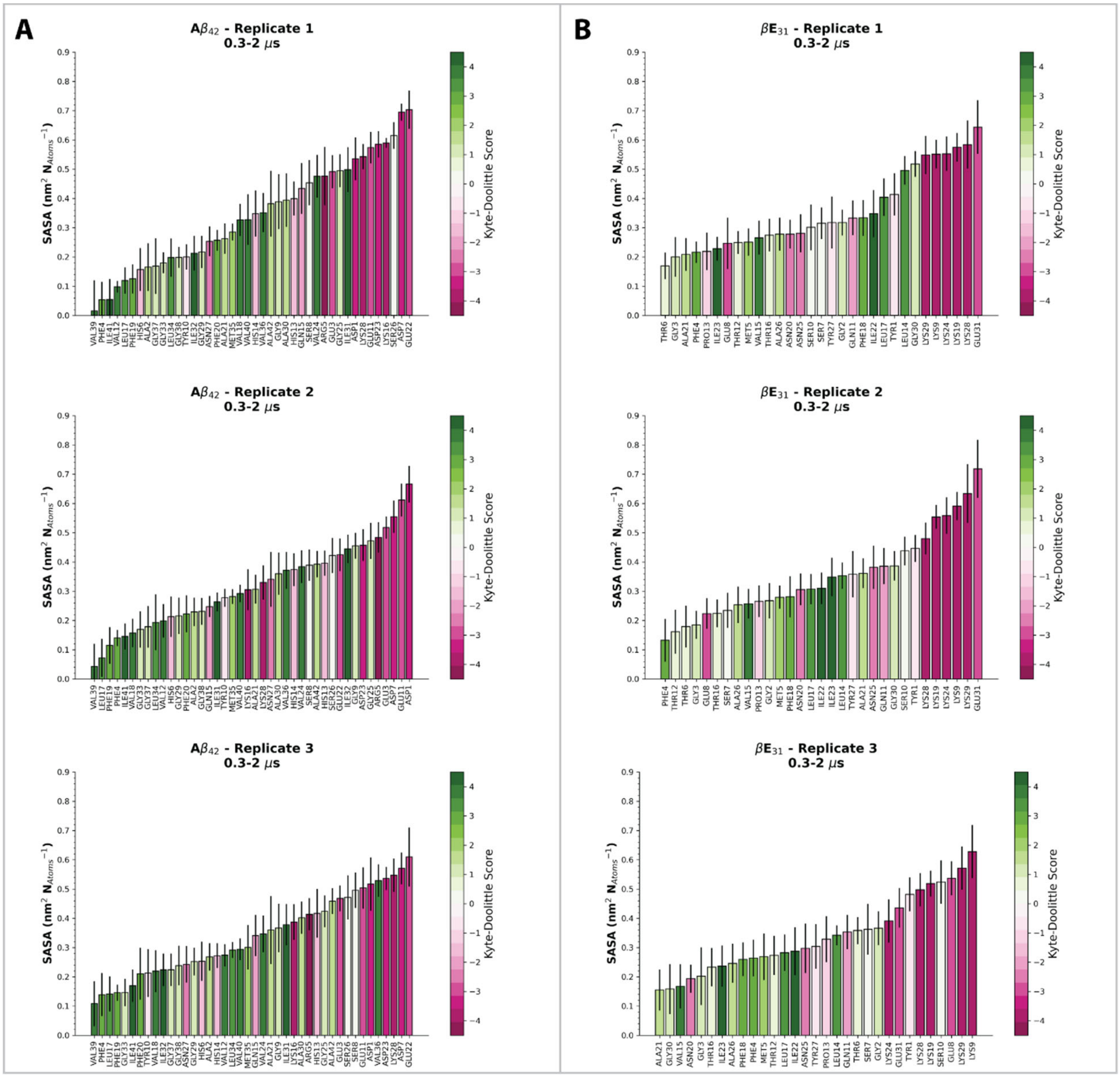

Figure S16. Inter-residue interaction occupancies for (A) Aβ$_{42}$ and (B) βE$_{31}$ hexamers. Distances between every possible residue pair were calculated and occupancy was defined as the fraction of frames a residue pair was within 0.6 nm cutoff. Left shows interaction occupancies greater than 10% of simulation time. This is shown separately to highlight differences, as low occupancies are common (right).

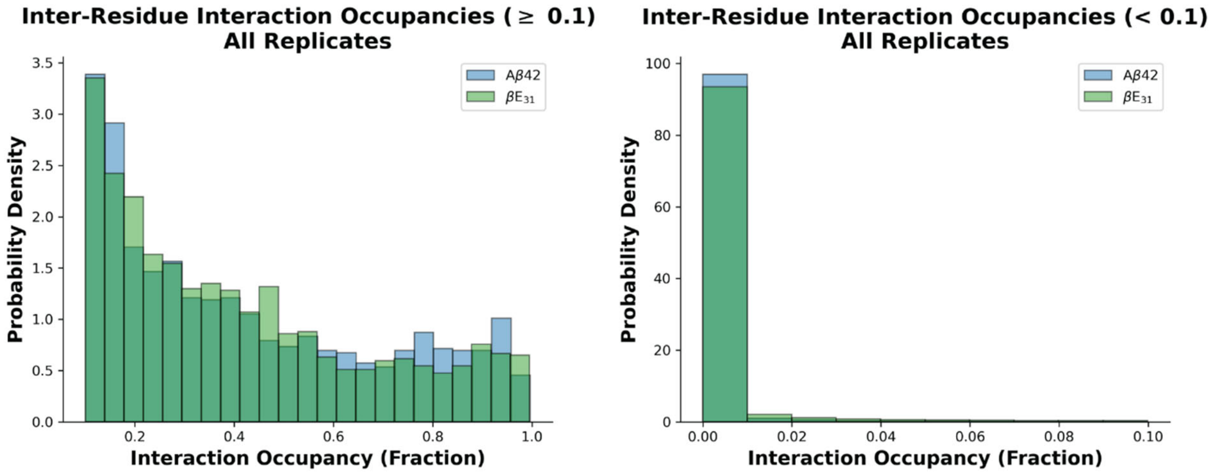

Figure S17. Inter-residue interaction occupancies for Aβ_{42} **and** βE_{31}**.** Distances between every possible residue pair were calculated and occupancy was defined as the fraction of frames a residue pair was within 0.6 nm cutoff. Left shows interaction occupancies greater than 10% of simulation time. This is shown separately to highlight differences, as low occupancies are common (right).

Table S4. The mean of pairwise Hamming distances of secondary structure content of residues 28-42 in $A\beta_{42}$ and $A\beta_{42}^{MUT}$ hexamer simulations.

	**$A\beta_{42}$**	**$A\beta_{42}^{MUT}$**
Replicate 1	2.7 ± 1.5	5.9 ± 3.0
Replicate 2	4.7 ± 2.6	4.1 ± 2.2
Replicate 3	2.7 ± 1.5	4.2 ± 2.4
**Mean**	**3.4 ± 1.9**	**4.7 ± 2.5**

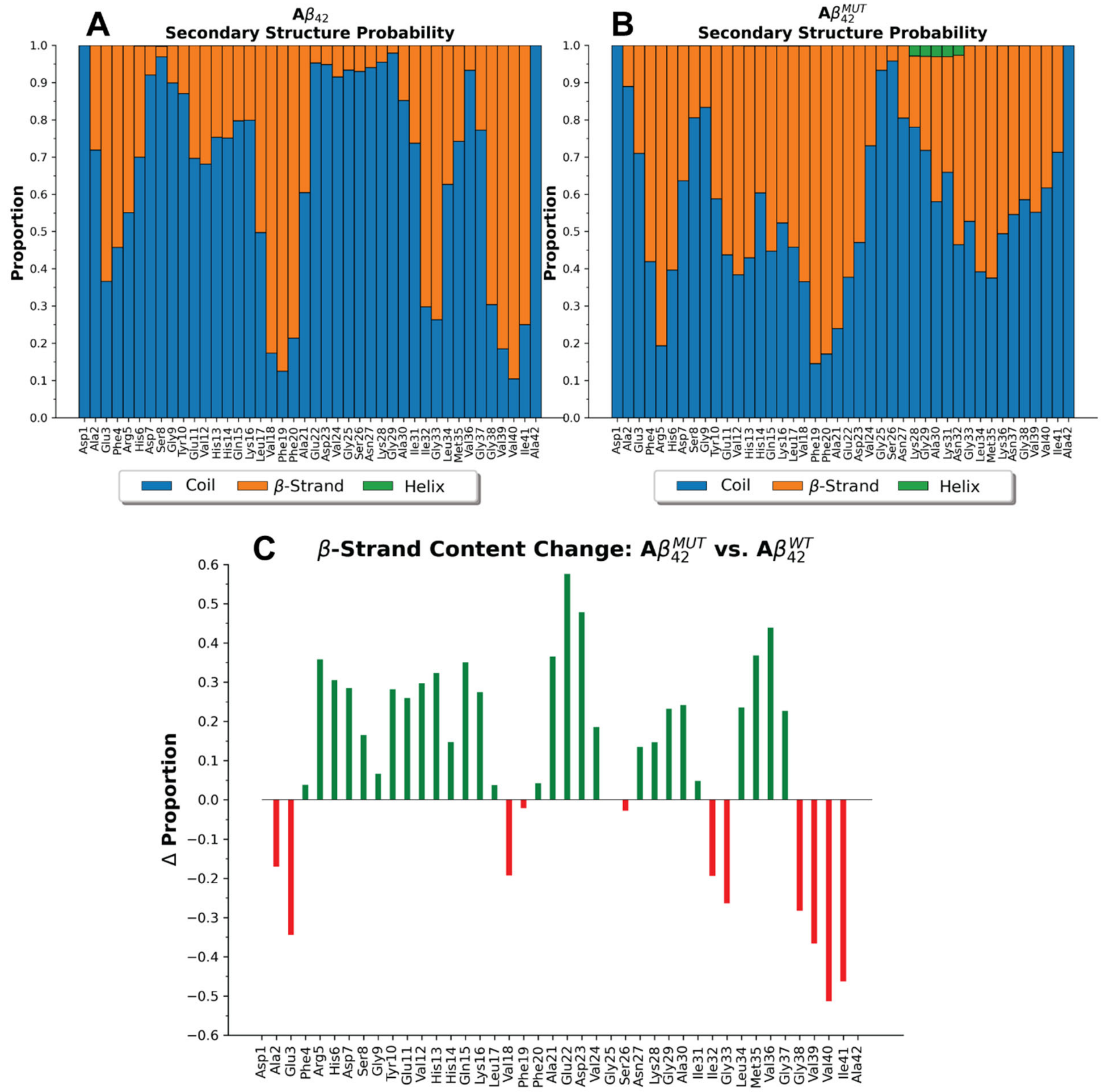

Figure S18. Secondary structure probabilities and changes for Aβ$_{42}$ and Aβ$_{42}$^{MUT}. Secondary structure probabilities over all replicates and over the entire 2 μs simulation for **(A)** Aβ$_{42}$ and **(B)** Aβ$_{42}$^{MUT} hexamers. **(C)** Change in β-strand content propensity from in hexameric Aβ$_{42}$^{MUT} relative to hexameric Aβ$_{42}$. Green indicates an increase in β-strand probability in hexameric Aβ$_{42}$^{MUT} relative to hexameric Aβ$_{42}$, and red indicates a decrease.

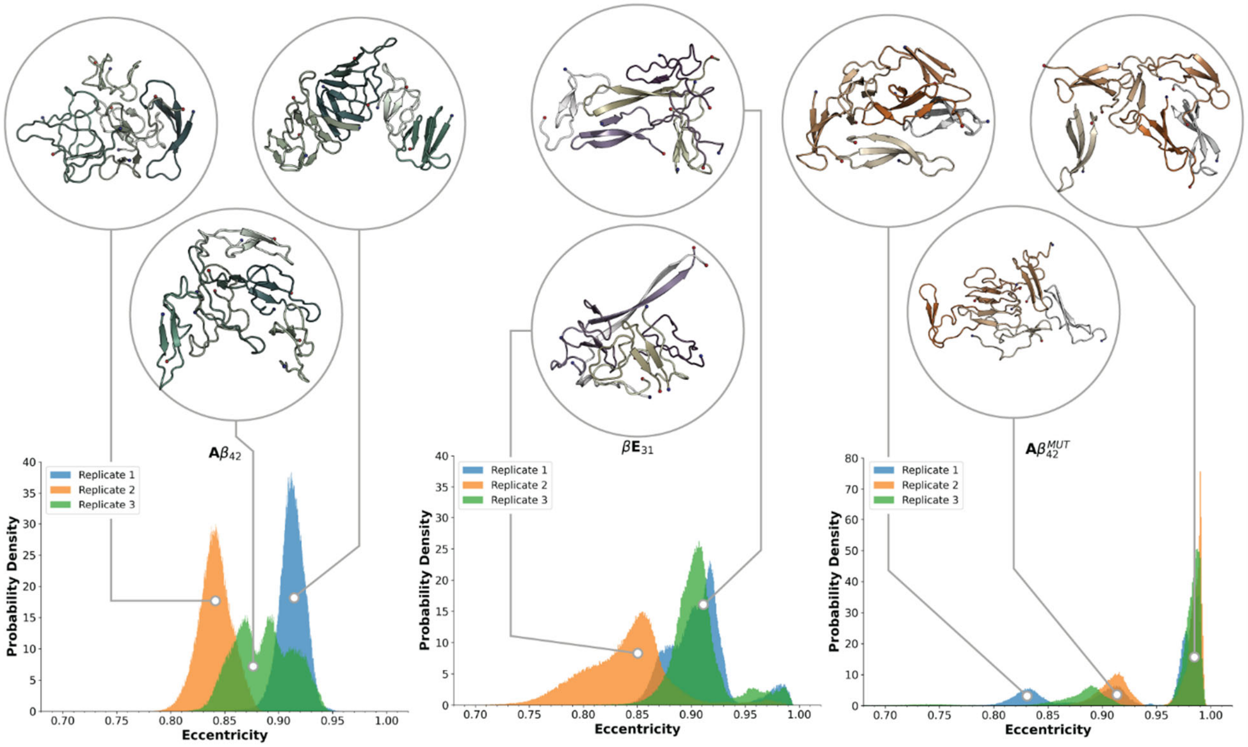

Figure S19. Distributions of eccentricity for (left) Aβ$_{42}$, (middle) βE$_{31}$, and (right) Aβ$_{42}^{MUT}$ hexamer simulations. Distributions represent eccentricity values sampled in the aggregated simulation period (0.3-2 μs) Snapshots show cluster structures corresponding to the area of the distribution.

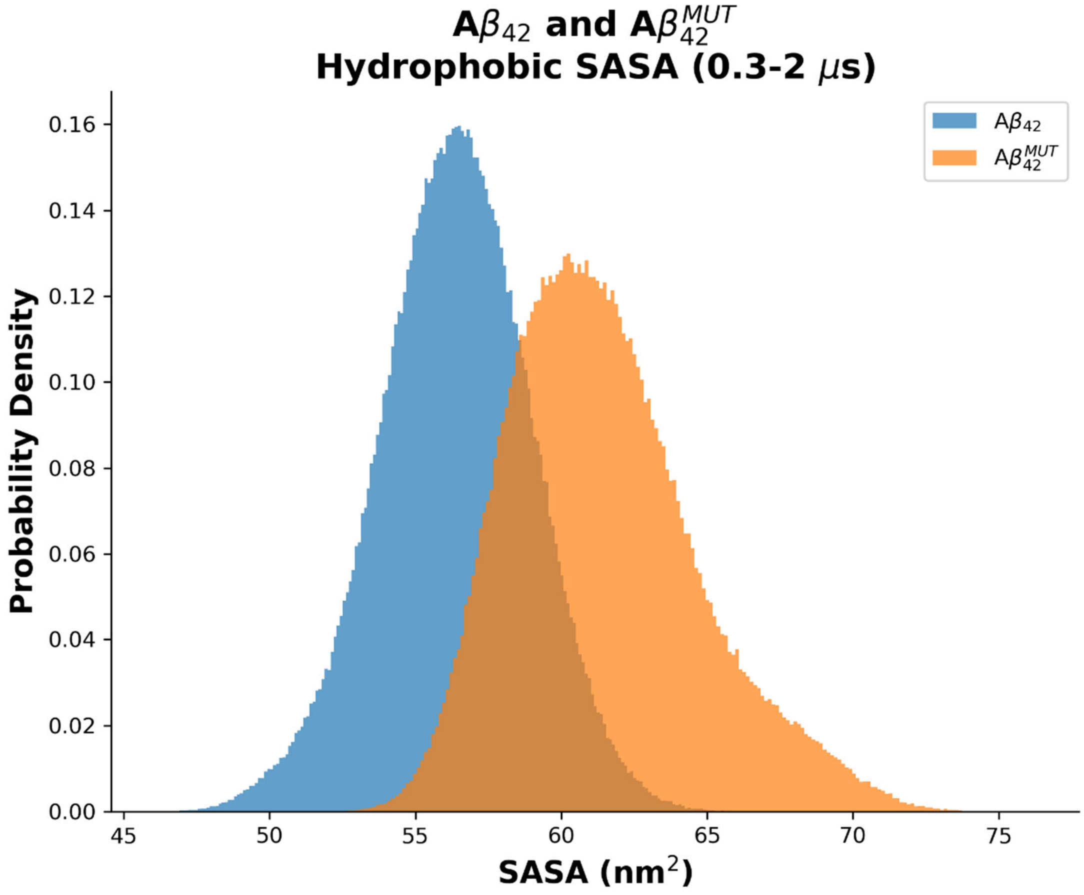

Figure 20. Distribution of hydrophobic solvent-accessible surface area (SASA) sampled for Aβ_{42} and Aβ_{42}^{MUT} hexamers. Distributions show SASA over the aggregated simulation period (0.3-2 μs). Distributions include data from all replicates.

A

B

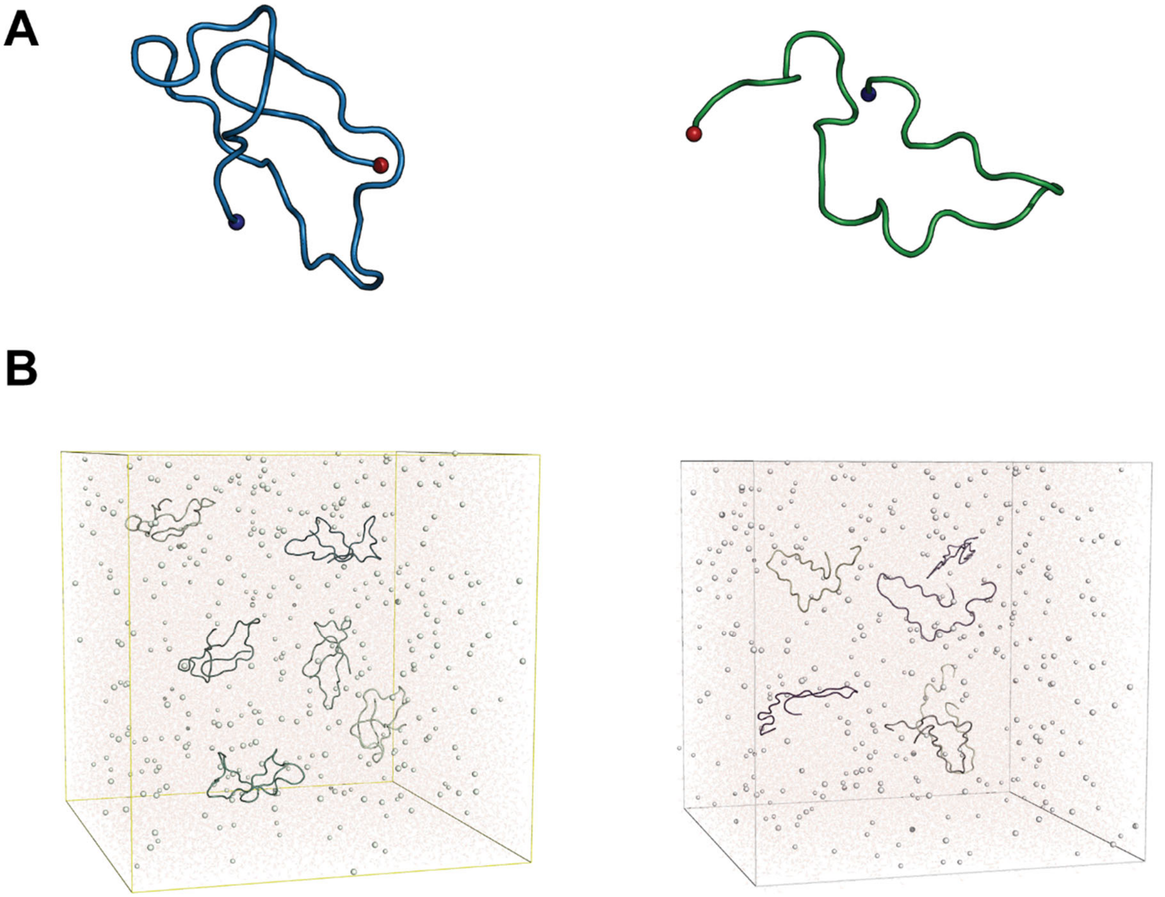

Figure S21. Starting structures for Aβ_{42} and βE_{31} simulations. (A) monomeric simulation starting structures, **(B)** hexameric simulation starting structures.

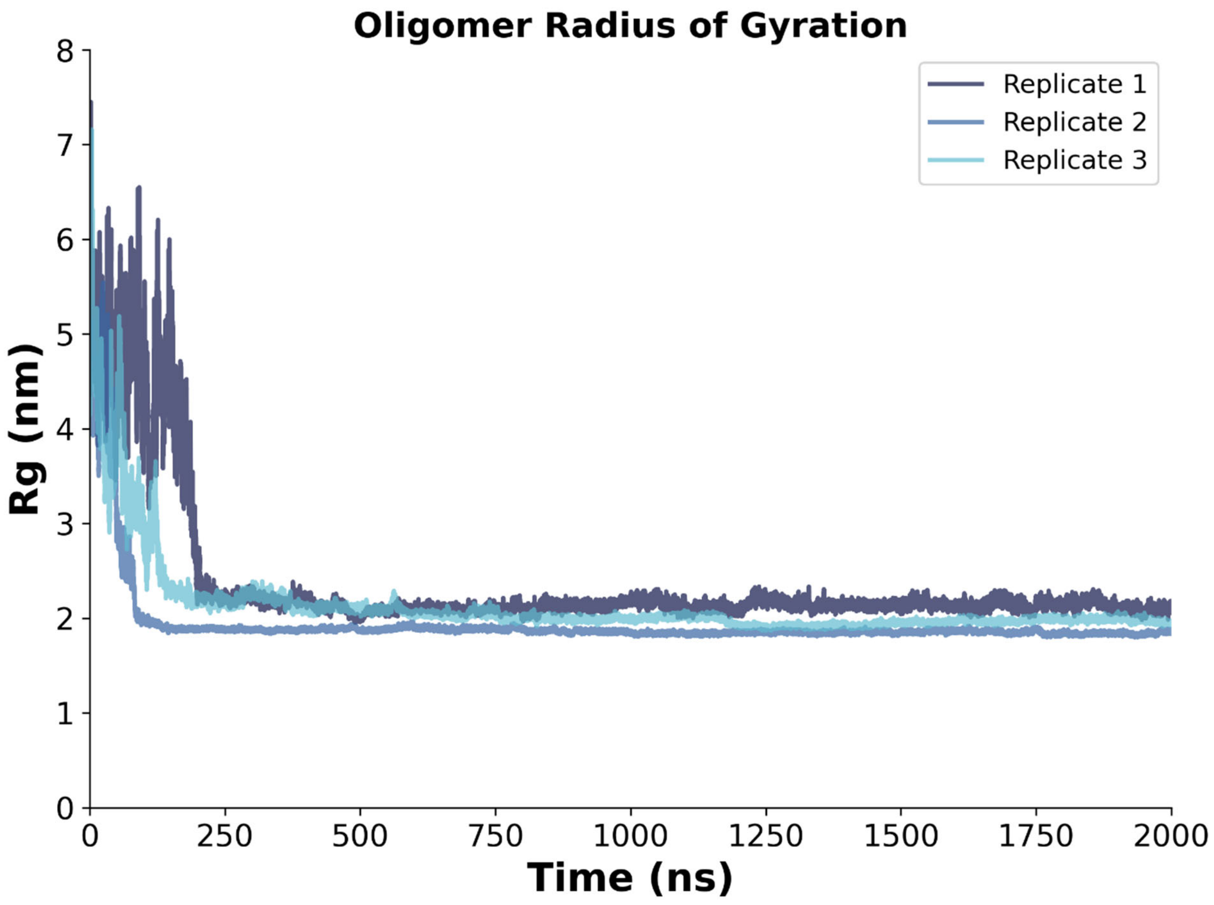

Figure S22. Radius of gyration over time for the Aβ_{42} hexamer, by replicate.

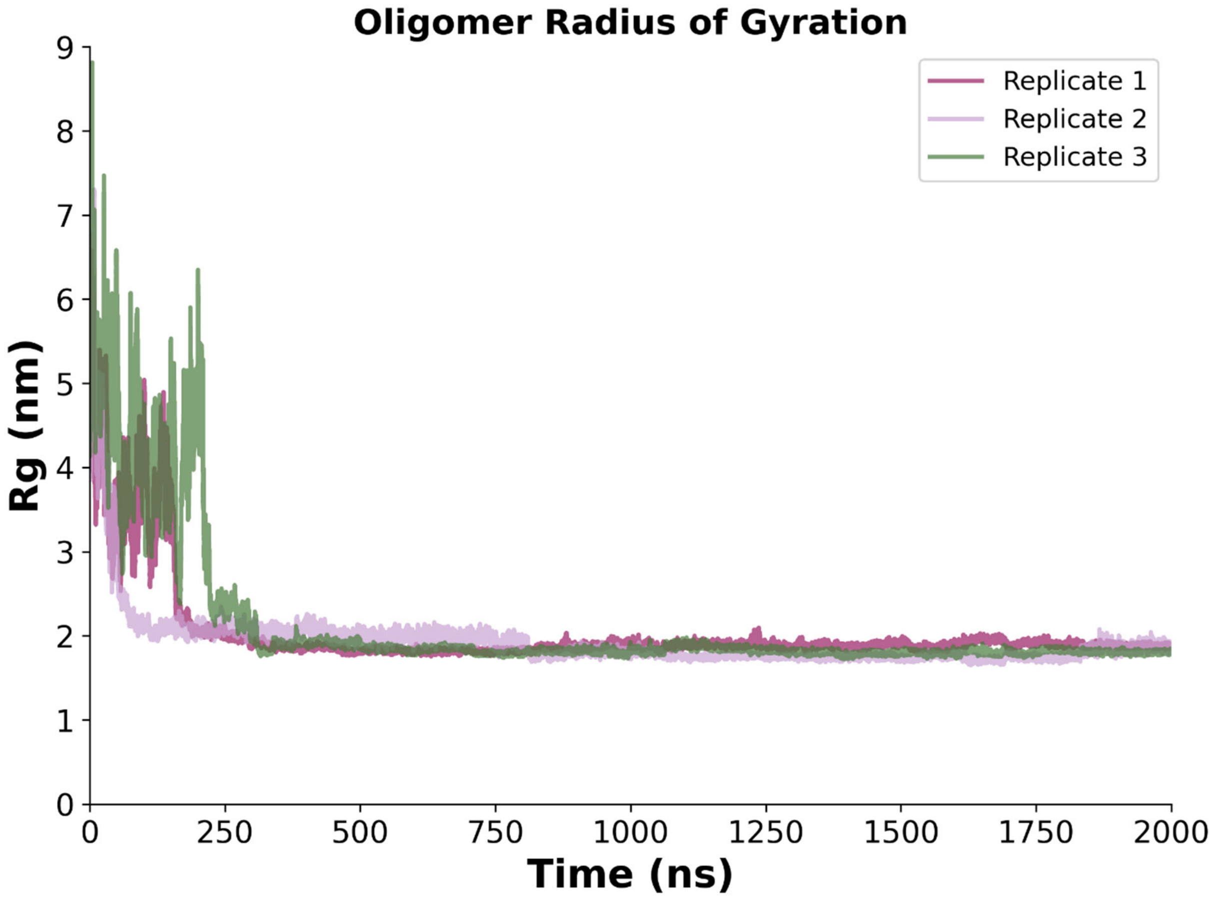

Figure S23. Radius of gyration over time for the βE_{31} hexamer, by replicate.
